# Meta-Profiles of Gene Expression during Aging: Limited Similarities between Mouse and Human and an Unexpectedly Decreased Inflammatory Signature

**DOI:** 10.1371/journal.pone.0033204

**Published:** 2012-03-07

**Authors:** William R. Swindell, Andrew Johnston, Liou Sun, Xianying Xing, Gary J. Fisher, Martha L. Bulyk, James T. Elder, Johann E. Gudjonsson

**Affiliations:** 1 Department of Genetics, Harvard Medical School, Boston, Massachusetts, United States of America; 2 Department of Dermatology, University of Michigan Medical School, Ann Arbor, Michigan, United States of America; 3 Department of Pathology, University of Michigan Medical School, Ann Arbor, Michigan, United States of America; 4 Division of Genetics, Department of Medicine, Brigham and Women's Hospital and Harvard Medical School, Boston, Massachusetts, United States of America; 5 Department of Pathology, Brigham and Women's Hospital and Harvard Medical School, Boston, Massachusetts, United States of America; 6 Division of Health Sciences and Technology, Harvard Medical School, Boston, Massachusetts, United States of America; University of Birmingham, United Kingdom

## Abstract

**Background:**

Skin aging is associated with intrinsic processes that compromise the structure of the extracellular matrix while promoting loss of functional and regenerative capacity. These processes are accompanied by a large-scale shift in gene expression, but underlying mechanisms are not understood and conservation of these mechanisms between humans and mice is uncertain.

**Results:**

We used genome-wide expression profiling to investigate the aging skin transcriptome. In humans, age-related shifts in gene expression were sex-specific. In females, aging increased expression of transcripts associated with T-cells, B-cells and dendritic cells, and decreased expression of genes in regions with elevated Zeb1, AP-2 and YY1 motif density. In males, however, these effects were contrasting or absent. When age-associated gene expression patterns in human skin were compared to those in tail skin from CB6F1 mice, overall human-mouse correspondence was weak. Moreover, inflammatory gene expression patterns were not induced with aging of mouse tail skin, and well-known aging biomarkers were in fact decreased (e.g., *Clec7a*, *Lyz1* and *Lyz2*). These unexpected patterns and weak human-mouse correspondence may be due to decreased abundance of antigen presenting cells in mouse tail skin with age.

**Conclusions:**

Aging is generally associated with a pro-inflammatory state, but we have identified an exception to this pattern with aging of CB6F1 mouse tail skin. Aging therefore does not uniformly heighten inflammatory status across all mouse tissues. Furthermore, we identified both intercellular and intracellular mechanisms of transcriptome aging, including those that are sex- and species-specific.

## Introduction

Intrinsic skin aging is characterized by the progressive accumulation of senescent cells, thinning of the epidermis, decline in cell-to-cell adherence, reduction of collagen content, formation of fine wrinkles, and gradual loss of elastic material [1], [2]. These features of normal skin aging can compromise health and well-being at older ages. Skin from older individuals, for instance, can lack the regenerative capacity required for wound healing and may provide a weakened immune barrier against pathogens, while also exhibiting an increased burden of somatic mutations that heightens susceptibility to melanomas and squamous cell carcinoma [1]–[5]. Senescent decline in skin function is simultaneous with broader systemic effects of normal aging, which involve diminished capacity to maintain homeostasis and overall loss of physiological reserve. The inter- and intra-cellular events associated with intrinsic skin aging parallel this broader aging process in certain respects, providing a model system to study mechanisms of aging [6]. In older individuals, for instance, the area occupied by elastic fibers in stained tissue sections is correlated between the reticular dermis and temporal artery, representing an extra-cellular feature of aging that is shared between the integumentary and vascular systems [7].

Health and appearance of human skin with age is closely linked to the synthesis of collagen and mechanisms that maintain the integrity of this abundant protein throughout the lifespan. With increasing age, abundance of both transforming growth factor β (TGF-β) and connective tissue growth factor (CTGF) declines in dermal fibroblasts, which contributes to reduced collagen synthesis and secretion [8]. Additionally, type I collagen fibrils become increasingly fragmented due to a self-perpetuating cycle in which increased oxidant levels promote an AP-1 and α2β1 integrin-dependent elevation of matrix metalloproteinase 1 (MMP-1) expression, and in turn MMP-1 cleaves extracellular collagen, resulting in loss of mechanical tension in dermal fibroblasts [9]. This loss of mechanical tension leads to greater production of intracellular oxidants within fibroblasts, which further heightens MMP-1 activity and reinforces the cycle by which extracellular matrix is degraded in aging skin [9]. The importance of these processes to aging is not restricted to skin alone. In heart, for instance, heighted TGF-β activity and excess collagen deposition are factors that promote fibrotic disease, which can decrease elasticity of ventricular walls and compromise contractile function [10]. In mice, shifts in the expression of procollagen-encoding genes (*Col1a2* and *Col3a1*) with age are among the most well-supported and widespread of all age-associated gene expression patterns [11]. These alterations in procollagen gene expression may, in part, reflect age-related attenuation of estrogen, growth hormone and/or insulin-like growth factor endocrine pathways [6], [12]–[16].

Genome-wide expression profiling provides broad insights that can assist development of more comprehensive skin aging models [17]–[20]. Such investigations have identified unexpected and previously unknown effects of intrinsic skin aging, but require extension in several ways. First, expression profiling studies have primarily focused on one sex (males) [17], [18], but effects of aging on gene expression in humans can be sex-specific [21], [22]. Second, while the influence of aging among mammalian cell types and organ systems is partly overlapping and partly tissue-specific [11], [23], [24], it is unclear which aspects of skin aging are among the core senescent processes that influence other organ systems, which might be broadly targeted by therapeutic compounds that prevent or delay onset of age-related conditions [25], [26]. Third, although large-scale shifts in gene expression occur with intrinsic skin aging, mechanistic underpinnings of these transcriptional changes remain unclear. On the one hand, the aging skin transcriptome is shaped by intracellular events and the activation or inhibition of transcription factors, such as ETS1, ETS2, AP-1, p53, E2F1 and NF-κB [8], [19], [27], [28]. At the same time, extracellular features of skin aging also influence the composition of RNA species within aging skin. These features include degradation of the extracellular matrix [9], decline in the average diameter of blood vessels [29], and inflammatory events with infiltration of aging tissues by, for example, macrophages, T-cells, B-cells, natural killer cells, and neutrophils [30]. A complete mechanistic model must tie together the major intracellular and intercellular features of skin aging, and a key challenge is development of analytical pipelines that can exploit large-scale genomic datasets for this purpose.

We used Affymetrix high density oligonucleotide arrays to provide a comprehensive analysis of transcriptome aging in sun-protected skin obtained from female and male human subjects, as well as in tail skin from CB6F1 mice of both sexes. We investigate broad correspondence between effects of natural aging in human and mouse skin. By analyzing transcriptome data within a meta-analysis framework, we highlight intrinsic features of skin aging that are closely tied to basic aging mechanisms, with the aim of establishing skin aging biomarkers that are best supported by existing data. *In silico* approaches are also used to identify both intercellular (inflammatory) and intracellular (cis-regulatory) mechanisms that underlie expression changes associated with advancing age, and our findings provide support for mechanisms that are sex-specific and/or species-specific.

## Results

### *In silico* inflammation profiling uncovers female-specific elevation of lymphocyte signatures with intrinsic aging of human skin

Genes for which expression is altered by aging in sun-protected skin (buttock and upper thigh) were identified from analysis of 31 female subjects (ages 18–75) and 31 male subjects (ages 18–60) (Table S1). Expression signatures of aging in female and male skin were compared with those generated from 25 other datasets, where each dataset included expression measurements from a combination of young and old subjects (e.g., airway epithelia, trachea, muscle, blood and central nervous system; all data were generated using the same Affymetrix Human Genome U133 Plus 2.0 platform; total of 573 arrays; Figure S1 and Table S1). From this integrative analysis, a partially overlapping set of gene expression responses to aging in skin and other organ systems could be discerned (Figure S1). For instance, among the top 50 genes increased by aging most frequently across tissues, 32 were also increased in female and/or male skin (e.g., *CLU*, *DSE*, *MT2A*; see Figure S1A). Likewise, among the top 50 genes most often decreased by aging, 24 were correspondingly decreased in female and/or male skin (e.g., *CA11*, *CHST2*, *DCAF11*; see Figure S1B). However, based upon analysis of overrepresented Gene Ontology (GO) terms, it was also clear that many effects of aging in human skin were sex-specific. In females, for instance, aging decreased expression of genes associated with diverse metabolic processes (e.g., ATP, acetyl-CoA and alkaloid metabolism), but such genes were not correspondingly decreased in males (Figure S2B).

**Figure 1:**
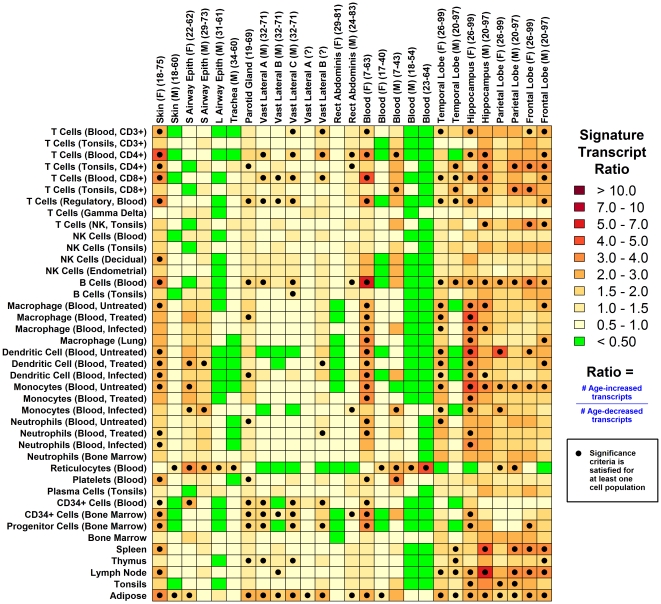
*In silico* inflammation profiles of aging in human tissues: Sex-specific lymphocyte signatures in skin. An algorithm for calculation of inflammation profiles based on microarray data was used to establish a gene expression-based mapping of inflammation events associated with aging in human skin and other tissue types (see Methods section and Figure 2 from Swindell et al. [31]). Colors reflect age-associated gene expression patterns among “signature transcripts” that exhibit elevated expression in different cell populations (listed in left margin). Dark red colors indicate cases in which signature transcripts for a given cell population increase with age (i.e., the ratio of age-increased to age-decreased signature transcripts is greater than one). Filled black circles indicate significant bias towards age-increased expression among signature transcripts (FDR-adjusted P<0.05; Fisher's exact test), suggesting increased abundance or infiltration of that cell population with aging. The opposite pattern, in which signature transcripts are disproportionately decreased with age, is indicated by green squares within the chart (indicative of decreased cell type abundance with age). For most cell population categories (rows), data from multiple cell populations was available, which permitted calculation of several signature transcript ratios (*r*_1_, *r*_2_…*r_n_*). In these cases, the maximum ratio is displayed in the chart if the median value of *r*_1_, *r*_2_… *r_n_* is greater than 0.50 (since, in this case, most evidence suggests bias towards age-increased expression among the *n* replicate sets of signature transcripts). The minimum ratio is displayed if the median value of *r*_1_, *r*_2_… *r_n_* is less than 0.50 (since, in this case, most evidence suggests bias towards age-decreased expression among the *n* replicate sets of signature transcripts).

**Figure 2:**
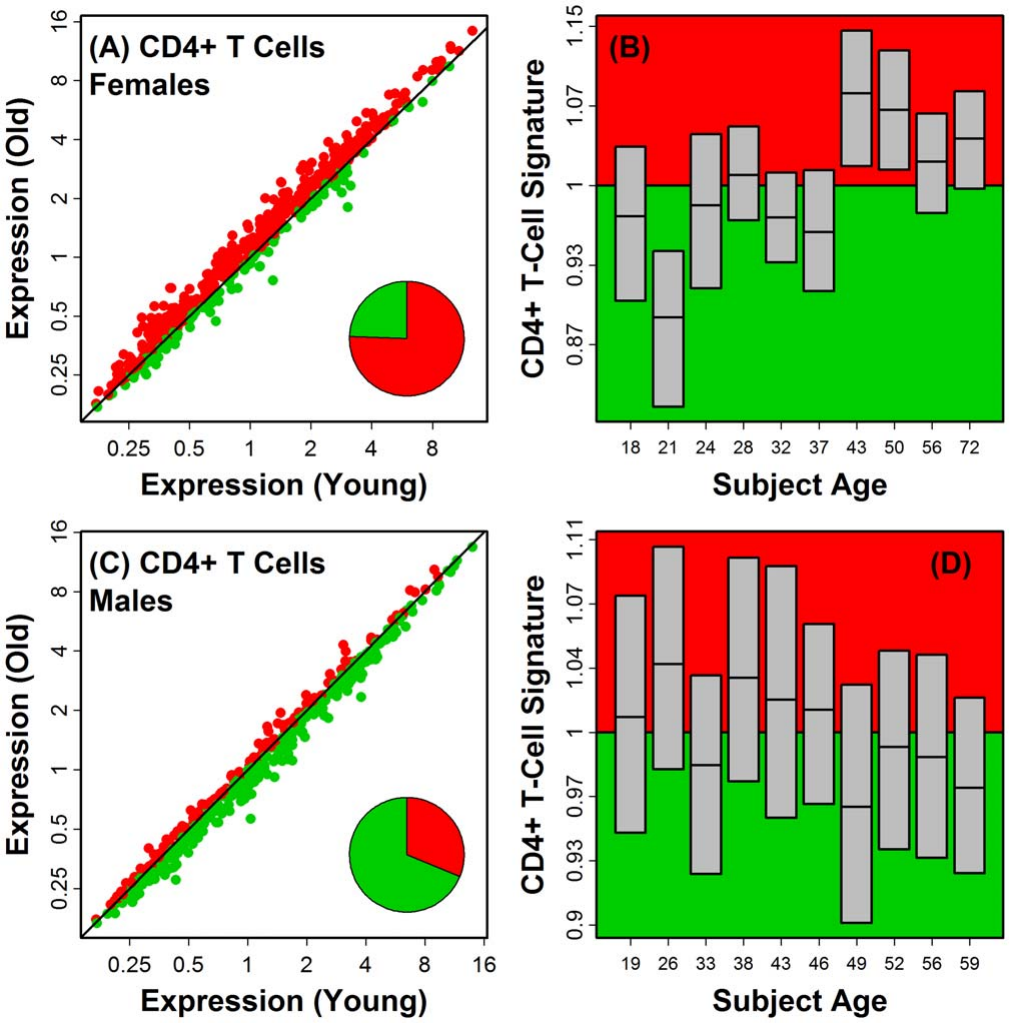
Signature transcripts of CD4+ T-cells exhibit sex-specific shifts in gene expression with age in human skin. We identified 500 probe sets (from the Affymetrix Human Genome U133 Plus 2.0 array) representing signature transcripts that exhibit high expression in CD4+ T-cells isolated from peripheral blood (based on data obtained under GEO series accession GSE14278). Scatterplots (A) and (C) display expression of these transcripts in skin samples from young and old human subjects. Red symbols represent probe sets with higher expression in old subjects, and green symbols represent probe sets with higher expression in young subjects. The relative proportion of these two probe set groups is indicated by the pie chart shown in each figure. In (A) (females), young subjects were between 18 and 22 years of age (*n* = 5) and old subjects were between 57 and 75 years of age (*n* = 5). In (C) (males), young subjects were between 18 and 27 years of age (*n* = 5) and old subjects were between 57 and 60 years of age (*n* = 5). Among all T-cell-associated probe sets, the average increase in old subjects was 11% in females, but in males, T-cell-associated probe sets decreased by 5.3% on average (P<0.001; two-sample t-test). Across all probe sets, moreover, fold-change differences between old and young mice were negatively correlated between the sexes (*r* = −0.13, P<0.001). In parts (B) and (D), subjects were divided into different age groups (*n* = 2–3 subjects per group). 40-year fold-change estimates (old/young) were then averaged across subjects belonging to the same age group for each of the 500 probe sets associated with the CD4+ T-cell signature. Grey boxes denote the median and inter-quartile range for the 500 fold-change estimates in each age group. The red background region denotes an average fold-change larger than one (i.e., signature transcripts are age-increased on average), while the green background region denotes an average fold-change less than one (i.e., signature transcripts are age-decreased on average).

In males, but not females, aging decreased expression of genes associated with immune-related GO terms (e.g., immune response, cellular defense, antigen processing and presentation; Figure S2D). This pattern could be due to sex differences in inflammatory events and localized shifts in lymphocyte abundance with advancing age [30]. To evaluate this possibility, we applied an algorithm for generating “inflammation profiles” based on microarray data, which focuses on “signature transcripts” highly expressed in immunocyte populations (or other cell types) in order to gauge whether there exists significant evidence for shifts in cell type abundance between two groups (e.g., young and old) [31]. This approach detects directional bias in the effects of aging among signature transcripts associated with specific cell types, and uses this information to infer shifts in cell type abundance with increased age (see Methods and Figure 2 from Swindell et al. [31]). For both female and male skin, transcripts with high expression in adipose tissue increased with age, supporting expansion of the subcutaneous adipose tissue compartment in both sexes (Figure 1). However, divergent inflammatory patterns in females relative to males were identified (Figures 1 and 2). In females, aging increased expression of transcripts associated with CD3+ T-cells, CD4+ T-cells, CD8+ T-cells, B-cells, macrophages, dendritic cells, monocytes and neutrophils (Figures 1 and 2). In males, these patterns were absent, and in contrast, there was evidence for decreased expression of transcripts associated with CD3+ T-cells, CD4+ T-cells, CD8+ T-cells and B-cells (Figures 1 and 2). More broadly, the female signature closely paralleled those observed in skeletal muscle and central nervous system regions, while that of males was more similar to airway epithelia, trachea and whole blood (Figure 1).

### Zeb1, AP-2 and YY1 motifs have female-specific associations with the expression profile of intrinsic human skin aging

The intracellular expression response to aging is coordinated, in part, by the activation or repression of transcription by sequence-specific transcription factors [8], [19], [27]. To identify candidate transcriptional regulators in aging skin, we assembled a dictionary of 541 DNA binding site motifs for mammalian transcription factors from the UniPROBE and Jaspar databases [32]–[34], and investigated whether abundance of each individual motif within 2 kb upstream and 200 bp downstream of transcription start sites was associated with increased or decreased expression with age (Figures S3 and S4). Two binding site motifs (Foxl1 and Cebpa) were associated with age-increased expression in both sexes (Figures S3A, S3C, S4A and S4C). However, among top-ranked motif-expression associations, these were the only patterns consistent between sexes (Figures S3 and S4). One top-scoring motif – for the TF Nfe2l1 – was associated with age-*decreased* expression in females but in contrast was associated with age-*increased* expression in males (see magenta labels in left margin of Figures S3B, S3C, S4B and S4C). Thus, sex-dependence was noted with respect to both inflammatory and putative cis-regulatory mechanisms in humans (Figures 1, S3 and S4).

We further investigated the associations between gene expression and motif density observed for putative Zeb1 (zinc finger E-box binding homeobox 1), AP-2 (transcription factor AP-2 alpha) and YY1 (Yin and Yang 1) DNA binding site motifs (Figures S3B and S4B). In each case, a larger number of motif occurrences near the transcription start site (2000 BP upstream to 200 BP downstream) was associated with decreased expression in aging female skin, but trends were attenuated or contrasting in male skin (Figure S5). These female-specific motif associations were further supported based upon analysis of intergenic sequences, intronic sequences and TSS-proximal sequences conserved among vertebrate species (see Methods; results for AP-2 are shown in Figure S6). With respect to intronic regions, associations between Zeb1, AP-2 and YY1 motifs and age-related expression diverged sharply between sexes, with significant but opposite patterns in females relative to males (Figures S6E–S6H).

### Lack of global correspondence between aging effects in human sun-protected skin and tail skin from CB6F1 mice in both sexes

**Figure 3:**
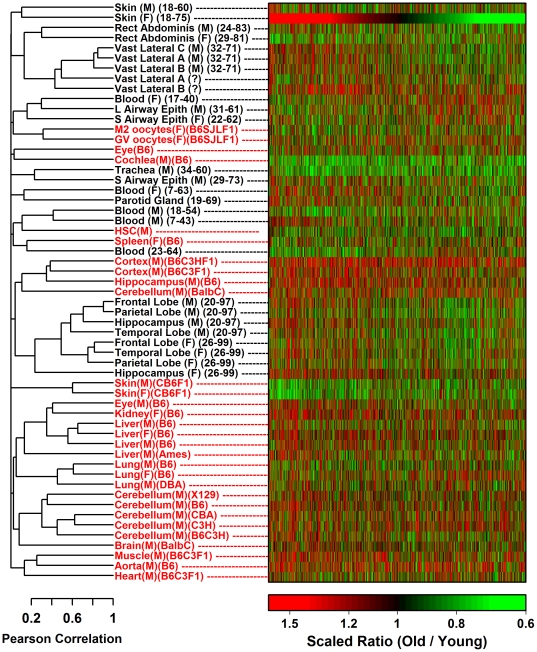
Unsupervised clustering of gene expression signatures from humans and mice reveals that aging effects are predominantly species-specific. We generated gene expression profiles for aging in human and mouse skin and compared these with 25 aging profiles from human tissues and 28 aging profiles from mouse tissues. All human profiles (black labels) were generated based on data from the Affymetrix Human Genome U133 Plus 2.0 array platform and all mouse profiles (red labels) were generated based on data from the Affymetrix Mouse Genome 430 2.0 array platform (Tables S1 and S2). The cluster analysis is based upon a total of 3785 human-mouse transcript pairs, which collectively, were associated with 3646 unique human genes and 3580 unique mouse genes. All human-mouse transcript pairs were significantly altered by aging with respect to at least three separate human profiles or at least three separate mouse profiles (FDR-adjusted P<0.05). Heat map colors denote an adjusted fold-change ratio (old/young) in units of standard deviations to permit visual and quantitative comparison across tissues and species. In humans, adjusted ratios were obtained by calculating the estimated 40-year fold change (old/young) for each transcript and dividing this ratio by the standard deviation of all 3785 ratios calculated in a given profile (i.e., each row of the heatmap). Similarly, in mice, adjusted ratios were obtained by calculating the 2-year fold change (old/young) for each transcript and dividing this ratio by the standard deviation of all 3785 ratios calculated in a given profile (i.e., each row of the heatmap). Further details on the experimental datasets used to generate human and mouse aging profiles is provided in the Methods section, Table S1 and Table S2.

**Figure 4:**
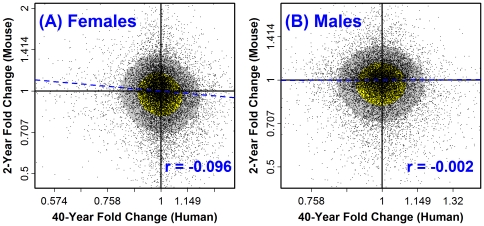
Weak association between aging effects in sun-protected human skin and tail skin from CB6F1 mice. The expression fold-change (old/young) over 40 years (human) or 2 years (mouse) was calculated for 15,203 transcripts associated with orthologous human and mouse genes. The scatterplot shows the human-mouse association between fold change estimates in (A) female human subjects versus female mice and (B) male human subjects versus male mice. The dashed blue line represents the least squares regression estimate, and the estimated Pearson correlation (*r*) is shown in the lower right quadrant (blue font). The yellow region in (A) and (B) outlines the central 50% of data points closest to the bivariate median (based upon Mahalanobis distance). The grey region outlines the central 80% of data points closest to the bivariate median (based upon Mahalanobis distance). A bivariate association, if present, would be indicated by an oblong (non-circular) shape of the yellow or grey region. In (A), the estimated correlation coefficient is significantly negative (*r* = −0.096; P<0.001). In (B), the estimated correlation coefficient is non-significant (*r* = −0.002; P = 0.77). Absence of significant correspondence between human and mouse aging effects was further supported based upon three other statistical methods for comparing gene expression profiles (see Figures S7, S8 and S9).

A previous investigation of aging in brain, muscle and kidney found limited correspondence between age-related expression profiles in humans and inbred C57BL/6 mice [24]. We thus evaluated gene expression in tail skin of young (5 month) and old (30 month) CB6F1 hybrid mice of both sexes (*n* = 5 per age-sex combination; Affymetrix Mouse Genome 430 2.0 Array platform). Inspection of aging signatures, including mouse and human skin combined with other mammalian tissues, highlighted a general pattern by which aging patterns in human tissues clustered together with each other and apart from those of mouse tissues (Figure 3). The human and mouse fold-change estimates (old/young) were negatively correlated or uncorrelated (Pearson *r* = −0.096 in females, P<0.001; *r* = −0.002 in males, P = 0.77; see Figure 4). This lack of significant similarity was confirmed based upon three other analytical methods, including examination of differential expression signature overlap [11] (Figure S7), rank-based AUC statistics [35] (P≥0.136; Figure S8) and gene set enrichment analysis [36] (P≥0.51; Figure S9). Global effects of aging on gene expression in CB6F1 mouse tail skin therefore do not have significant similarity to those in sun-protected human skin.

### Aging does not increase expression of *Lyz1*, *Lyz2*, *Clec7a*, *Cd48*, *Tyrobp* and *Mpeg1* in tail skin of male and female CB6F1 mice

**Figure 5:**
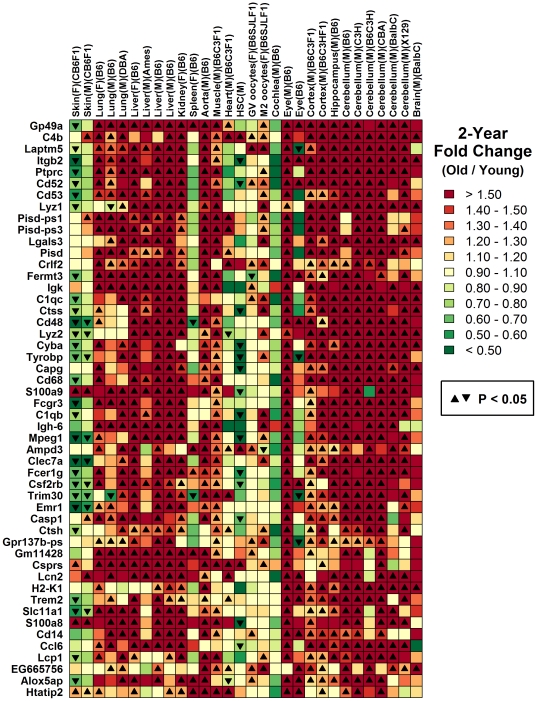
Aging in mouse tail skin leads to an aberrantly weak inflammatory signature with decreased expression of *Lyz1*, *Lyz2*, *Clec7a*, *Cd48*, *Tyrobp* and *Mpeg1*. Gene expression profiles associated with aging in mouse tail skin (first two columns in chart) were aligned with 28 other aging profiles from a panel of mouse tissues. All profiles were generated from datasets that included young and old mice, where the same Affymetrix Mouse Genome 430 2.0 array platform was used to generate each dataset (see Methods and Table S2 for further detail). The chart lists in rank-order the top 50 genes increased by aging across all profiles (based upon the total number of significant (age-increased) results among all profiles for a given gene). Colors within the chart denote the estimated 2-year fold change (old/young) for a given gene (row) and profile (column) (see color scale), while up and down-triangles denote significant aging effects (comparison-wise P<0.05). Several alternative versions of this chart with different gene-ranking schemes are shown in Figure S10.

**Figure 6:**
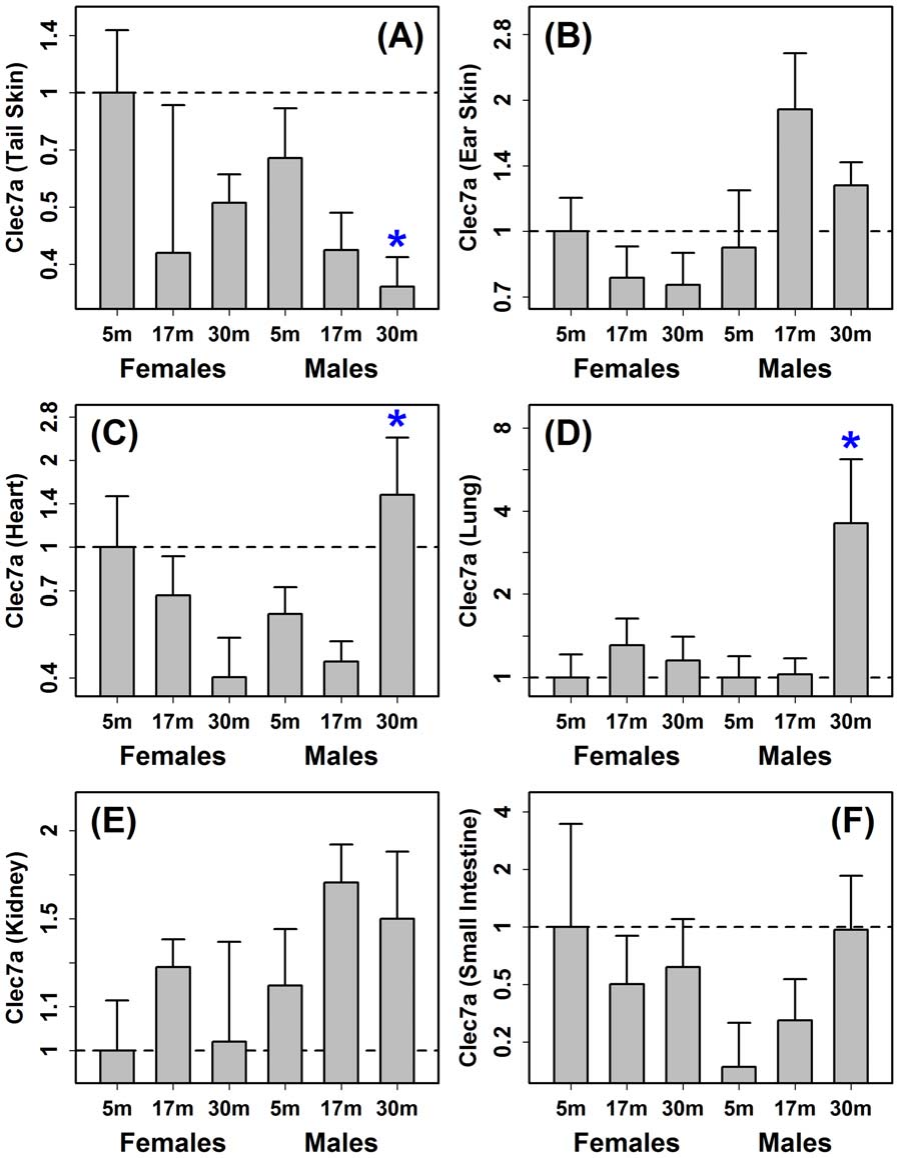
*Clec7a* expression is decreased with age in tail skin but increased by age in heart and lung from CB6F1 mice. RT-PCR was used to evaluate the expression of *Clec7a* in mice from three age groups (5 months, 17 months and 30 months) and both sexes (*n* = 5–6 mice for each sex/age combination). The expression of 18S ribosomal RNA (Rn18s) was used as an internal control gene for calculation of relative expression in each sample. Plots (A)–(F) show mean expression levels in each group (± one standard error), which have been normalized to the average expression level of the young (5 month) female group. In each plot, and for each sex, the average expression of middle-aged (17 month) and old groups (30 months) was compared to that of the young group (5 months). A blue star is used to indicate a significant difference based upon a two-sample non-parametric statistical test (P<0.05; either Wilcoxon or Kruskal-Wallis rank sum test).

A robust and striking effect of aging in laboratory mice is elevated expression of genes associated with immune activation, including cathepsin S (*Ctss*), complement components (e.g., *C1qa*), cluster of differentiation antigens (*Cd48*) and lysozyme (*Lyz1* and *Lyz2*) (see Figure 6 from [11]). We compared genes regulated by aging in mouse tail skin with age-regulated genes identified in 28 other datasets with young and old samples from a diverse panel of mouse tissues (e.g., lung, liver, kidney, spleen, aorta, muscle, heart, cochlea, eye and central nervous system; all studies used the same Affymetrix Mouse Genome 430 2.0 platform; total of 295 arrays; Figures 5 and S10; Table S2). As expected, the most consistent effect of aging in mouse tissues was elevated expression of membrane-associated immune response genes, including glycoprotein 49 A (*Gp49a*), lysosomal-associated protein transmembrane 5 (*Laptm5*), CD52 antigen (*Cd52*), CD53 antigen (*Cd53*), C-type lectin domain family 7 member a (Clec7a), lysozyme 1 (*Lyz1*) and lysozyme 2 (*Lyz2*) (see Figure 5). Strikingly, however, in mouse tail skin, expression of these genes was decreased, in both sexes, but particularly in females (Figure 5). This aberrant pattern was further illustrated by inspection of genes associated with specific immune-associated GO terms (e.g., activation of immune response, positive regulation of defense response, acute inflammatory response), which were decreased with age in CB6F1 tail skin, but increased in most other mouse tissues (see Figure S11B). We confirmed by quantitative RT-PCR that expression of *Clec7a*, *Lyz1* and *Lyz2* was decreased with age in mouse tail skin (Figures 6, S12 and S13). Further evaluation indicated that the pattern was specific to tail skin and not, for instance, an unusual aging effect in CB6F1 mice. In particular, one or more of the evaluated genes (*Clec7a*, *Lyz1* and *Lyz2*) was significantly elevated with age in ear skin, heart, lung, kidney and small intestine of CB6F1 mice (Figures 6, S12 and S13).

### Aging of tail skin in CB6F1 mice promotes decreased expression of transcripts associated with antigen presenting cells in both sexes

**Figure 7:**
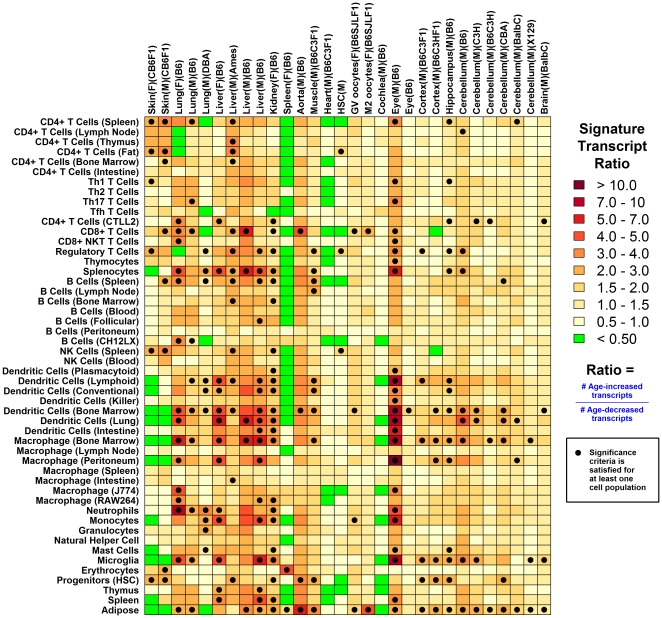
*In silico* inflammation profiles of aging in mouse skin and other tissues: Tail skin aging leads to decreased expression of genes associated with antigen-presenting cells. An algorithm for calculation of inflammation profiles based on microarray data was used to establish a gene expression-based mapping of inflammation events associated with aging in mouse tail skin (CB6F1 strain) and other tissue types [31] (see Methods). This figure can be interpreted as described in the Figure 1 legend and is based upon the influence of aging on the expression of “signature transcripts” associated with different cell populations (listed in left margin). Red colors in the chart denote evidence for an age-related expansion or infiltration of the cell population type listed in each row (sees color scale and Figure 1 legend). The opposite trend is denoted by green colors (see color scale and Figure 1 legend).

C-type lectin domain family 7 (*Clec7a*/*Dectin-1*) was unexpectedly decreased by aging in mouse tail skin (Figures 5 and 6) and is expressed at high levels in leukocyte subsets, including dendritic cells, macrophages, monocytes and neutrophils [37]. We thus hypothesized that mouse tail skin would exhibit concerted declines in the expression of transcripts associated with specific lymphocyte subsets. Consistent with this, inflammation profiles indicated decreased expression of transcripts associated with antigen presenting cells (APC; DC and macrophages) in old mouse tail skin as compared to young skin (both sexes), along with weak elevation of transcripts associated with CD4+ T-cells (Figures 7 and S14). This decline contrasted with a general trend observed across mouse tissues, in which APC-associated transcripts were frequently elevated with age (Figure 7). Moreover, although aging decreased expression of genes expressed in the T- and B-cell subsets in human males, an age-related decrease in the expression of transcripts associated with APC was not noted in either female or male human skin (Figure 1). Thus, differential age-related declines in DC and macrophage-associated expression contribute to divergent effects of transcriptome aging in human skin and CB6F1 tail skin (Figures 3 and 4).

### Zeb1 and Prrx2 DNA binding site motifs are associated with gene expression changes with aging of CB6F1 mouse tail skin

**Figure 8:**
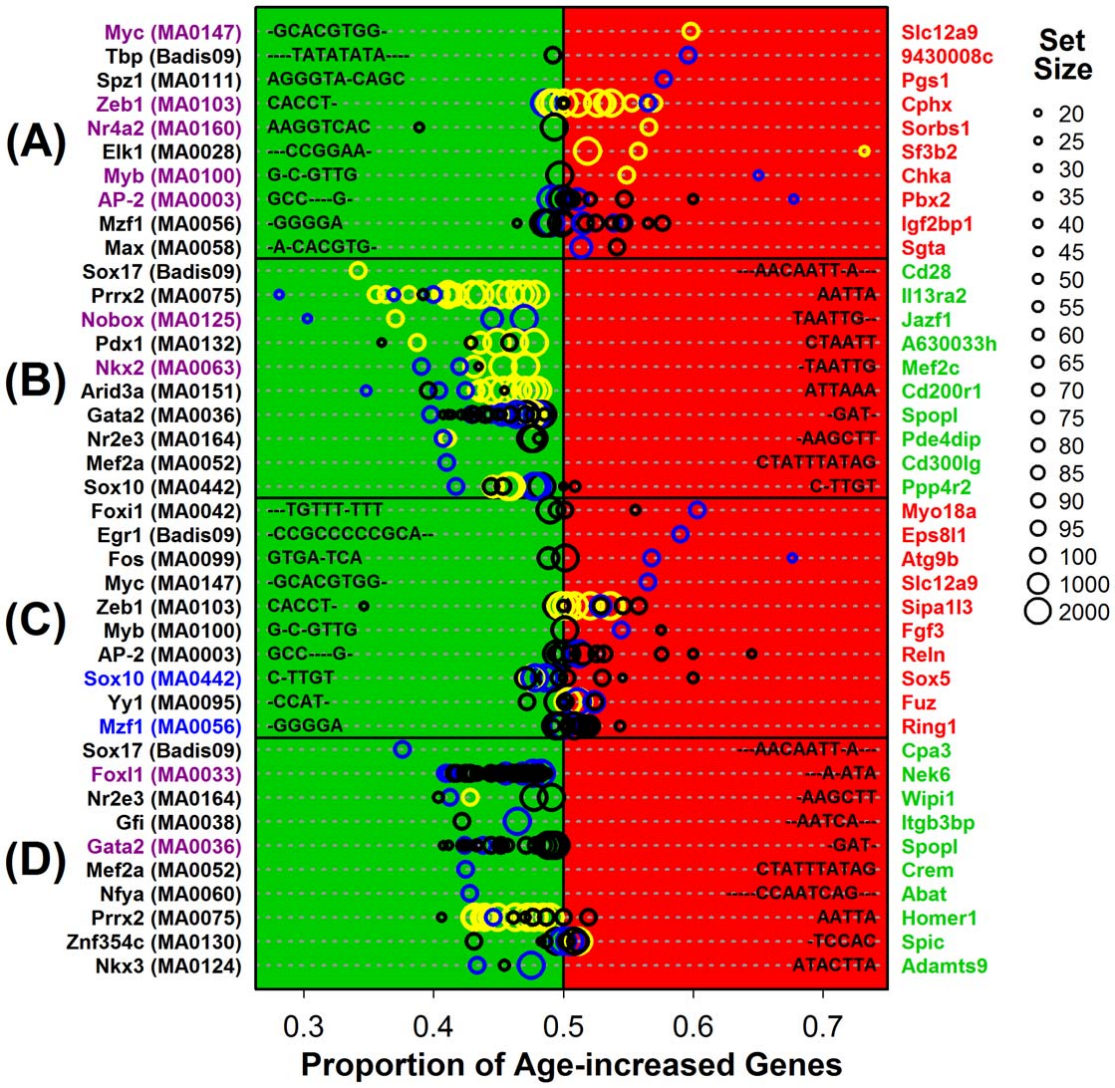
Top-ranked motifs associated with aging effects in mouse tail skin (CB6F1 strain): Overlap with top-ranked human motifs but dissimilar associations with aging. The chart lists the top-scoring motifs associated with genes (A) increased by aging of tail skin in female mice, (B) decreased by aging of tail skin in female mice, (C) increased by aging of tail skin in males, and (D) decreased by aging of tail skin in males. For each motif (left margin), gene sets associated with a varying number of motif occurrences (in the region 2000 BP upstream/200 BP downstream of the transcription start site) were derived. For any one motif, gene sets with fewer motif occurrences were always larger than those sets containing genes with more motif occurrences (see legend in right margin). Thus, larger symbols correspond to larger gene sets with few motif occurrences while smaller symbols correspond to smaller gene sets with a larger number of motif occurrences (see legend). For each set, the proportion of age-increased to age-decreased genes was calculated (open symbols and horizontal axis; see Figure S3 and S4 legends). A motif-expression association is indicated by deviation of this proportion from 0.50, with blue symbols corresponding to gene sets for which the estimated proportion differs significantly from 0.50 (comparison-wise P<0.05; Fisher's exact test), and yellow symbols corresponding to sets significant at a more stringent threshold (FDR-adjusted P<0.05; Bejamini-Hochberg correction). We noted two motif-aging associations that, for the same sex, were consistent in direction with those identified in humans (blue font; compare with Figures S3 and S4). However, we noted nine associations that, for the same sex, were contrasting in direction compared to those identified in humans (magenta font; compare with Figures S3 and S4). For each motif listed, candidate target genes are listed in the right margin (red labels for age-increased genes; green labels for age-decreased genes; see Figure S3 and S4 legends).

We screened 541 mammalian transcription factor DNA binding site motifs for association with gene expression changes in tail skin from aging mice (Figures 8 and S15). As compared to humans (Figure S3 and S4), consistent trends between species included age-increased expression of genes associated with elevated myeloid zinc finger 1 (Mzf1) and SRY-box 10 (Sox10) binding site density in males (Figures 8C and S15C; blue labels). Notably, however, we identified five motifs associated with age-increased expression in tail skin from female CB6F1 mice, but age-*decreased* expression in human females (i.e., AP-2, Myb, Myc, Nr4a2 and Zeb1; Figures 8A and S15A), along with two motifs associated with age-decreased expression in female mice but age-*increased* expression in human females (i.e., Nkx2 and Nobox; Figures 8B and S15B). Moreover, in males, two motifs were linked with age-decreased expression in mice but age-*increased* expression in humans (i.e., Foxl1 and Gata2; Figures 8D and S15D). Thus, in line with the lack of correspondence between age-associated gene expression patterns in humans and mice (Figures 3 and 4), as well as disparate shifts in inflammatory gene expression patterns with aging in each system (Figures 1 and 7), we identified contrasting motif-expression associations with aging of human and mouse skin.

In mice, motifs strongly associated with tail skin aging included Zeb1 (zinc finger E-box binding homeobox 1) and Prrx2 (paired related homeobox 2) (Figures 8 and S15). These motif-expression associations were consistent between mouse sexes, with the Zeb1 motif linked to increased expression with age, and the Prrx2 motif linked to decreased expression with age (Figure S16). In each case, moreover, a similar motif-expression association was discernible in intergenic and intronic regions of mouse genes, as well as in TSS-proximal sequences conserved among vertebrate species (see Methods; results for Zeb1 are shown in Figure S17).

### Clusterin (*Clu*) expression is elevated with age in CB6F1 mice and decreased in long-lived *Pit1*(*dw*/*dw*) mice

**Figure 9:**
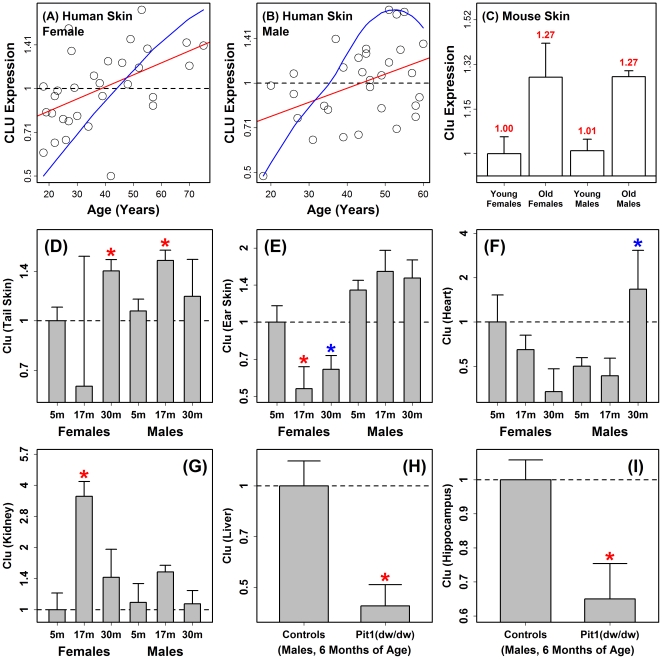
Clusterin (*Clu*) expression is altered by aging in human and mouse organs and *Clu* expression is decreased in long-lived *Pit1*(*dw*/*dw*) mice (liver and hippocampus). Plots (A) and (B) display the expression of human clusterin (*CLU*) relative to age, where each open circle indicates the microarray-based expression estimate in sun-protected skin from an individual female or male. Red lines correspond to the least squares estimate for the regression of expression versus subject age, and blue lines represent the same regression but obtained using the Loess procedure. Panel (C) displays mean microarray-based expression levels of mouse *Clu* (±1 standard error) in tail skin from young (5 month) and old (30 months) CB6F1 mice (*n* = 5 per group). Panels (D)–(G) display average expression of mouse *Clu* in mice from three age groups (5 months, 17 months and 30 months) and from both sexes (*n* = 5–6 mice for each sex/age combination; RT-PCR with 18S ribosomal RNA used as an internal control gene for calculation of relative expression). Each panel (D)–(G) displays the mean expression levels in each group (± one standard error) normalized to the average expression level of the young (5 month) female group. For each sex, the average expression of middle-aged (17 month) and old groups (30 months) was compared to that of the young group (5 months). A red star indicates a significant difference relative to the youngest group of the same sex (P<0.05; two-sided two-sample t-test). A blue star is used to indicate a significant difference based upon a two-sample non-parametric statistical test (P<0.05; either Wilcoxon or Kruskal-Wallis rank sum test). Panels (H) and (I) display average expression of mouse clusterin (*Clu*) in long-lived *Pit1*(*dw*/*dw*) dwarf mice as compared to littermate controls with normal phenotypes (*n* = 6 per genotype; males; six months of age; *Actb* was used as an internal reference for calculation of relative expression).

We found that aging has a unique effect in mouse tail skin, as compared to other tissues, with an unexpectedly weak inflammatory gene expression signature (Figures 5 and S11B). Nevertheless, we expected that some aspects of tail skin aging would be closely tied to basic aging mechanisms in other tissues, both in mice and humans. To identify such responses, we aligned expression signatures of aging from human and mouse skin (both sexes), in combination with signatures from a diverse panel of human and mouse tissues (Figure S18; Tables S1 and S2). Inspection of these meta-profiles uncovered expression responses to aging that were similar across many human and mouse tissues (e.g., *Clu*, *Erbb2ip* and *C5ORF13*/*D0H4S114*; see Figure S18). The protein-damage biomarker *Clu*, for instance, has been linked to Alzheimer's disease in genetic association studies [38]–[41], and was previously identified as part of a common signature of genes with elevated expression in aging mammalian tissues [23]. In agreement, our meta-profiles indicated that *Clu* expression increased with age in 13 of 27 human aging profiles and 14 of 30 mouse aging profiles (Figures 9 and S18A). This is a better-supported aging effect than, for instance, elevated expression of *Cdkn2a* (*p16INK4a*) [42], which was increased by aging in 10 of 27 human profiles but was likewise elevated in only 4 of 30 mouse profiles (data not shown).

Further evaluation by RT-PCR revealed that *Clu* is elevated with age in cardiac tissue (males) and kidney (females) of CB6F1 mice, but surprisingly, decreased with age in ear skin (Figures 9D–9G). One application of well-supported gene expression biomarkers of aging is to judge whether intrinsic aspects of the aging process have been influenced by genetic or dietary interventions (e.g., GH/IGF-1 mutations or calorie restriction). As a test of this concept, we evaluated expression of *Clu* in GH-deficient *Pit1*(*dw*/*dw*) mice, which exhibit increased longevity and are viewed as a mouse model of healthy aging [43]. In both liver and hypothalamus, expression of *Clu* was diminished in *Pit1*(*dw*/*dw*) mice at 6 months of age, consistent with a “delayed aging” phenotype of *Pit1*(*dw*/*dw*) mice (P = 0.005; Figures 9H and 9I). Expression of *Clu* is thus sensitive to key endocrine pathways that modulate development of age-related pathology in mice.

## Discussion

Senescent decline of skin function provides a model for understanding basic biological mechanisms of aging that operate in mammalian organ systems and cell types. We have used genome-wide expression analysis to identify intercellular (inflammatory) and intracellular (cis-regulatory) mechanisms that account for the large-scale shifts in gene expression observed with intrinsic aging of human and mouse skin. In female subjects, inflammatory gene expression was heightened with age, with decreased expression of genes near regions of high Zeb1, AP-2 and YY-1 motif density. In male subjects, however, these effects were contrasting and absent, respectively. Such sex disparities were not observed in mouse tail skin, and at a broad level, there was little human-mouse correspondence between aging effects. Comparison of aging signatures across multiple datasets revealed that, surprisingly, aging in tail skin does not increase expression of immune response genes typically elevated with age (e.g., *Clec7a*, *Lyz1*, *Lyz2*, *Cd48* and *Tyrobp*). We thus present a counter-example to what is the most robust expression signature of aging known to occur in mice. This unexpected result is consistent with a decline in the abundance of antigen presenting cells (APCs) with aging of tail skin in CB6F1 mice, and we propose that this is one factor that contributes to the observed disparity of aging effects between human sun-protected skin and mouse tail skin.

Genome-wide expression profiling studies have repeatedly observed that transcripts elevated with age are linked to inflammation and immune response [11], [23], [44]–[47]. These expression patterns are probable indicators of inflammation and infiltration of aging tissues by leukocyte populations [30]. In this study, we have generated *in silico* profiles that characterize such shifts in lymphocyte abundance with aging in both humans and mice (Figures 1 and 7) [31]. In human skin, females exhibit an age-related increase in the expression of transcripts associated with T-cells, macrophages, DC, monocytes and neutrophils, while males exhibit decreased expression of transcripts associated with T- and B-cells (Figures 1 and 2). This sexually dimorphic pattern parallels that observed in the human central nervous system, with heightened inflammatory gene expression with aging in females as compared to males [21]. It is possible that anti-inflammatory properties of female sex hormones, such as estrogen, contribute to this sex disparity in skin and the central nervous system [48]–[50]. In females, estrogen levels decline with age, but more precipitously following menopause, while in males, dominance of estrogen relative to testosterone increases with age in a more continuous fashion [51]. These temporal dynamics are consistent with the gene expression signature associated with CD4+ T-cells, which was age-independent in females prior to the age of 40, and subsequently exhibited its strongest elevation with respect to the early 40's age group (approximately 40–45; see Figure 2B). In contrast, for male subjects, the CD4+ T-cell signature exhibited a slight and continuous decline with advancing age (Figure 2D).

Previous work has identified transcription factors associated with skin aging, including ETS1, ETS2, AP-1, p53, E2F1 and NF-κB [8], [19], [27], [28]. In this study, we identified motif-expression associations that include transcription factors not previously implicated in skin aging mechanisms (e.g., Zeb1, AP-2 and YY1 motifs in female human subjects, see Figures S3 and S4; Zeb1 and Prrx2 in female and male mice, see Figures 8 and S15). These results provide data-driven hypotheses for future work, although we expect that further experimental evaluation will be necessary to understand the potential regulatory role of these transcription factors in young versus old skin tissue. Importantly, however, our findings give emphasis to the sex- and species-specific character of these motif-expression associations, suggesting that sex and species must be carefully accounted for both in the analysis of genomic data and in mechanistic studies of such factors and their contribution to skin aging. The Zinc finger E-box binding homeobox 1 (Zeb1) transcription factor provides an illustrative example. This transcription factor was associated with age-decreased expression among (human) female subjects, but not males (Figures S5A and S5B). In mice, however, Zeb1 motif abundance was linked to age-*increased* expression in both males and females, contrasting strongly with the result obtained in human females (Figures S16A and S16B). Age-related shifts in estrogen dominance might contribute to the sex-specific Zeb1-expression association observed in humans. For instance, the gene encoding Zeb1 (*Tcf8*) is induced by estrogen and/or progesterone in chick oviduct, mouse uterus, human smooth muscle cells, and an osteosarcoma cell line, although these effects vary among estrogen-sensitive cell lines [52]–[54]. These Zeb1-mediated estrogen effects could impact skin aging phenotypes, since Zeb1 represses cellular senescence in mouse embryonic fibroblasts [55] and also represses the synthesis of *Col1a1* in osteoblasts [56], *Col1a2* in vascular smooth muscle cells [57], and both *Col2a1* and *Col11a2* in cartilage [58], [59].

Mechanistic correspondence between senescent processes in humans and mice is an important issue in aging biology, since functional studies of individual genes and their influence on aging frequently use mice, and because mice are used to screen drugs and identify interventions that might increase lifespan and healthspan in humans [25], [26]. Several of our findings are consistent with a previous study, which reported little broad correlation between age-associated gene expression patterns in brain, muscle and kidney tissue from humans and inbred C57BL/6 mice [24]. One explanation of these results is that aging processes differ fundamentally between humans and laboratory mice, possibly due to known effects of laboratory adaptation on the GH/IGF-1 endocrine pathway in mice [60], or to species differences in the accumulation of senescent cells and/or oxidative stress burden with aging [61], [62]. However, the following three points warrant consideration. (i) Transcriptional effects of aging in skin may depend upon the skin region that is sampled (e.g., keratinized versus weakly-keratinized regions) [20]. We cannot exclude the possibility, therefore, that stronger human-mouse correspondence would have been observed if other skin regions, with different histological properties, had been examined from either human subjects or mice [20]. Indeed, our findings show that expression of the lysozyme genes (*Lyz1* and *Lyz2*) were decreased with age in tail skin, but increased with age in ear skin (Figures S12 and S13). (ii) Despite the lack of broad correspondence between human and mouse aging effects, we could identify individual genes and gene sets with relatively species-convergent or species-divergent aging patterns (see Figures S18 and S19). For instance, points of convergence include negative regulation of epithelial cell proliferation, peptide cross-linking, collagen fibril organization and tumor necrosis factor cytokine production (Figure S19), while divergent points include positive regulation of αβ T-cell activation, leukocyte cell-cell adhesion, positive regulation of NF-kappaB transcription factor activity, and autophagy (Figure S19). (iii) Human-mouse similarity of transcriptional aging effects may be strengthened if human aging effects are estimated from a cohort that includes very old individuals. For example, we observed that (central nervous system) transcriptional aging signatures estimated from cohorts that include very old subjects (e.g., above 95 years old) did in fact cluster together with transcriptional aging signatures derived from mouse brain tissues (see Figure 3). On the other hand, in the context of experimental aging research, investigators have argued the counter-point, and have suggested that very old subjects do not, in fact, provide good models for understanding basic aging mechanisms [63].

Many datasets have now been generated for characterizing the aging transcriptomes of humans and mice [11], [23], [61]. It is important to emphasize, however, that both technical and biological factors limit consistency between studies, such as noise in genome-wide expression assays, idiosyncratic characteristics of mouse strains, disparities in sample processing methods, and stochastic aspects of aging processes. We estimate, for instance, that overlap among results from microarray-based gene expression studies of aging in human and mouse tissues is typically less than 20% at a comparison-wise type I error rate of 0.05 (Figure S20). From this perspective, the necessity of meta-analysis for establishing a strong foundation for future work is apparent, and we and others have emphasized this approach [11], [23], [61], [64]. While no individual gene exhibits a completely consistent pattern across datasets, we were able to identify well-supported single-gene patterns associated with aging in multiple tissues from humans and mice [11], [23], [64] (see Figure S18). Our results show, for instance, that clusterin (*Clu*) expression is elevated with age in multiple organs of CB6F1 mice (tail skin, heart and kidney) and that expression is also diminished in liver and hippocampus of “slow aging” Snell mice that lack circulating levels of GH and IGF-1 [38]–[41] (Figure 9). This result underscores a link between a well-supported age-associated expression pattern and an endocrine mutation known to alter aging trajectory in mammalian systems. We anticipate that meta-analyses will, in coming years, further orient our focus towards high-confidence aging effects with connections to basic aging mechanisms, and that this may lead to a more precise operational definition of aging.

Mammalian aging has been linked to a broad range of pathological processes at the cellular and histological levels, including inflammation, accumulation of oxidative stress damage, formation of advanced glycation end products, and diminished protein homeostasis [65]. In skin, these basic mechanisms of aging interact with declines in collagen synthesis and collagen degradation, with progressive declines in the integrity of the extracellular matrix [8], [9]. The transcriptome shifts that occur with aging can yield new hypotheses for mechanisms that participate in these processes. Here, we have provided evidence for concerted shifts in the expression patterns of genes associated with T-cells, B-cells and dendritic cells with increased age, along with directional shifts in the expression of genes associated with key transcription factors, such as Zeb1 in both humans and mice. Interestingly, these trends had sex- and species-specific characteristics, and in mice, were coupled to decreased expression of *Clec7a*, *Lyz1* and *Lyz2* with increased age. These findings provide data-driven insight into the mechanisms that shape the aging transcriptome, which, in turn, can provide guideposts towards development of more comprehensive conceptual models of skin aging. Future studies of these mechanisms may facilitate development of therapeutics that improve the likelihood of healthy aging outcomes in mammals.

## Methods

### Ethics statement

This study was conducted in compliance with good clinical practice and according to the Declaration of Helsinki principles. Informed written consent was obtained from all human subjects, under protocols approved by the institutional review board of the University of Michigan (HUM00037994). All animal protocols were approved by the University of Michigan committee on the use and care of animals (018 ARF 5614).

### Microarray analysis of sun-protected human skin

The population cohort and sample processing methods for human microarray data has been described in a recent report [66]. In brief, 62 healthy individuals were recruited from areas surrounding Detroit, Michigan between October 2001 and November 2002 (*n* = 31 women 18–75 years of age; *n* = 31 men 18–60 years of age). Skin biopsies were obtained from the buttock or upper thigh region, and throughout we have assumed that these skin regions are relatively sun-protected and thus predominantly influenced by intrinsic aging rather than the extrinsic “aging” associated with chronic UV light exposure. Processed samples from each subject were hybridized to Affymetrix Human Genome U133 Plus 2.0 arrays, which include probe sets corresponding to 54675 human transcripts. Raw microarray data has been deposited in the NCBI Gene Expression Omnibus database and is accessible through accession number GSE13355. Human Genome U133 Plus 2.0 array annotation was obtained from release 31 of the NetAffx database [67].

### Microarray analysis of mouse tail skin (CB6F1 strain)

CB6F1 hybrid mice were obtained from NIA (*n* = 5 males at 5 months of age; *n* = 5 females at 5 months of age; *n* = 5 males at 17 months of age; *n* = 5 females at 17 months of age; *n* = 6 males at 30 months of age; *n* = 6 females at 30 months of age). For these treatments, “young” mice correspond to those at 5 months of age, and “old” mice correspond to those at 30 months of age. Median lifespan for this hybrid mouse strain is reported to be 28.5 months [68]. Mice were fasted for 6–8 hours and then sacrificed by CO_2_ inhalation. Upon sacrifice, ear, back and tail skin was collected along with internal organs, and samples were stored in RNAlater (Qiagen cat. no. 76106) and placed at 4°C for 24 hours before transitioning to −20°C. For microarray analyses, we have focused on mouse tail skin because this region has relatively low density of hair follicles in correspondence with the more interfollicular nature of human skin. Additionally, mouse tail skin compares favorably with human skin due to its relative enrichment in epidermis and attenuation of the panniculus carnosus, a subcutaneous muscle layer prominent in dorsal regions of mice but nearly absent in humans [69]. All samples were disrupted using a rotor-stator homogenizer and RNA extractions were performed using the Qiagen RNeasy Fibrous Tissue kit (Qiagen cat. no. 74704), with on-column DNAase digestion (Qiagen cat. no. 79254). Extracted RNA was quantified using the NanoDrop spectrophotometer and RNA quality was evaluated using the Agilent Bioanalyzer. Processed samples from young and old mice were hybridized to Affymetrix Mouse Genome 430 2.0 oligonucleotide arrays, which include probe sets for more than 39,000 transcripts selected from GenBank, dbEST and RefSeq. Mouse Genome 430 2.0 array annotation was obtained from release 31 of the NetAffx database [67]. Raw data have been deposited in the NCBI Gene Expression Omnibus database and are accessible through accession number GSE35322.

### Statistical analysis of aging effects in human and mouse skin

Quality of array hybridizations was evaluated according to standard metrics, including degradation scores based upon 3′ to 5′ trends in expression measurements, average background and the percentage of transcripts with detectable expression levels [70]. Expression scores for all arrays were calculated using robust multichip average (RMA) [70], and both human and mouse skin datasets were adjusted for batch effects using Empirical Bayes methods [71]. In both mice and humans, functions from the Limma linear modeling package were used to estimate model coefficients associated with aging effects [72], and FDR-adjusted p-values were calculated using the Benjamini-Hochberg method [73]. For mouse tail skin data, aging effects were evaluated by a two-sample treatment comparison between young and old mice [72]. With respect to human sun-protected skin, expression values were available across a continuous span of ages. The effects of aging on gene expression were thus evaluated using iteratively reweighted least squares robust regression [74]. This approach estimates model coefficients by iteratively minimizing a loss function that assigns reduced weight to larger residuals, thereby attenuating the influence of outlying observations [74]. To implement this method, we used the “robust” option in the “lmFit” function from the Limma linear modeling package (available from Bioconductor) [75], which provides an interface to the MASS package “rlm” function with default settings [76]. Default settings for the “rlm” function initialize the procedure by obtaining ordinary least squares coefficient estimates, with subsequent refitting and reweighting of residuals using the “Huber function” with coefficient value of 1.345 [74]. Weights for individual observations were not provided during the initiation step, such that all observations were weighted equally when obtaining the initial least square coefficient estimates [76].

Both human and mouse analyses were performed using RMA-derived scores in which gene expression is quantified on a log_2_ rather than absolute scale. In human regression analyses, we expected that expression estimates on the log_2_ scale would have a linear association with individual age. For each of the 54,675 probe sets represented on the Human Genome U133 Plus 2.0 array, we evaluated whether including a quadratic term in our models led to a significant decrease in model deviance (i.e., F-test for lack of fit) [74], but did not obtain significant evidence in favor of this type of non-linear association between log_2_-based gene expression and individual age (FDR-adjusted P≥0.99 for both sexes). This supported adequacy of the linear regression approach in this context, and we expected that any opposing trends for some genes would be mitigated by our use of robust regression analysis [74], [76]. We note, however, that we have estimated 40-year fold-change in humans and 2-year fold-change in mice on an absolute scale (e.g., see Figures S1 and S10). For these fold-change estimates, we have assumed that gene expression changes with age are linear on an absolute scale. Since this assumption may not be valid for all genes, our fold-change estimates should be interpreted as a summary measure, which is accurate in cases for which gene expression on an absolute scale changes in linear fashion with age, but represents only the average rate of change across the lifespan in cases for which gene expression on an absolute scale changes in non-linear fashion with age.

Overrepresentation of gene ontology (GO) terms within specific sets of genes was evaluated using a conditional version of Fisher's exact test [70], [77]. With this approach, significance of a GO term is evaluated based upon a filtered set of associated genes, which excludes any gene associated with both that term and a more specific child term that is already significantly overrepresented. The main advantage of this approach is that results are decorrelated with less redundancy within a set of GO terms identified as significantly overrepresented with respect to genes increased or decreased by aging. We note, however, that significant GO terms are identified based upon p-values haven't been corrected for multiple hypothesis testing, since an appropriate p-value correction has not yet been established in this context [70]. In all GO analyses (Figures S2 and S11), overrepresentation of terms among age-regulated genes was evaluated with respect to a background of genes with detectable expression in the majority of human or mouse skin samples (12,500 human genes; 14,681 mouse genes). Presence or absence of transcripts in human or mouse skin samples was evaluated based upon p-values generated from the Wilcoxon signed rank test as implemented in the MAS 5.0 algorithm [70], [78].

### Meta-analysis of age-associated gene expression patterns

Meta-analysis of age-associated gene expression patterns was carried out using data available from either the Gene Expression Omnibus or ArrayExpress databases (accession numbers for human data: GSE876, GSE5086, GSE9419, GSE9103, GSE11906, GSE11375, GSE11882, GSE16028, GSE19743; accession numbers for mouse data: GSE3150, GSE3253, GSE4332, GSE4786, GSE6591, GSE8150, GSE10000, GSE10965, GSE11291, GSE11667, GSE13799, GSE21716, GSE22317, E-MEXP-839, E-MEXP-1504; Tables S1 and S2). Additionally, we have utilized a complete dataset provided as supplemental material from a previous study [45]. All datasets used the same Affymetrix oligonucleotide array platforms that we have utilized to generate expression data in human and mouse skin (i.e., the Human Genome U133 Plus 2.0 and Mouse Genome 430 2.0 array platforms). Statistical analysis of aging effects was carried out using two-sample comparisons (young versus old) or robust linear regression with age as a single predictor variable (see above). We have not incorporated cDNA array data from the AGEMAP project [24] into our analysis because the AGEMAP cDNA platform provides only partial coverage of the mouse genome (i.e., 8932 mouse genes), because AGEMAP cDNA array data have already been meta-analyzed in a previous study [11], and because differences between Affymetrix oligonucleotide and cDNA array platforms render direct comparisons difficult to interpret [79]–[81].

### Calculation of human and mouse inflammation profiles

Procedures followed for calculation of inflammation profiles have been described in a previous publication [31]. To generate human profiles (Figure 1), a database of 1382 Affymetrix Human Genome U133 Plus 2.0 array hybridizations was manually assembled using annotated CEL files downloaded from Gene Expression Omnibus. In total, the 1382 CEL files belonged to 309 cell population treatments, where each treatment contained two or more replicate CEL files associated with a given cell population (average of 1382/309 = 4.47 replicates per treatment). Signature transcripts associated with each treatment were identified by comparison to an independent reference set of 61 samples (representing treatment *A* in Figure 2 from [31]), consisting of array hybridizations involving a heterogeneous collection of human tissues (e.g., cerebellum, skin, colon, pancreas, see GEO accession number GSE7307). Likewise, to generate mouse profiles (Figure 7), a database of 871 Affymetrix Mouse Genome 430 2.0 array hybridizations was manually assembled using annotated CEL files downloaded from Gene Expression Omnibus. In total, the 871 CEL files belonged to 317 groups, where each group contained two or more replicate CEL files associated with a given cell population (average of 871/317 = 2.75 replicates per group). Signature transcripts associated with each treatment were identified by comparison to an independent reference set of 47 samples (representing treatment *A* in Figure 2 from [31]), consisting of array hybridizations that collectively involved a heterogeneous collection of mouse tissues (e.g., hippocampus, heart, kidney; see GEO accession number GSE10246). Given these procedures, signature transcripts associated with a given cell population treatment (either mouse or human) represent transcripts with high expression in that treatment, as compared to a diverse panel of other tissue types. Signature transcripts were identified using two-sample comparisons (cell population versus reference) and linear modeling methods implemented in the Limma package [31], [72]. For each cell population treatment, the ratio *n*_1_/*n*_2_ was calculated, where *n*_1_ represents the number of signature transcripts increased by age, and *n*_2_ represents the number of signature transcripts decreased by age. Additionally, an adjusted ratio *n*_1*_/*n*_2*_ was also calculated, which is identical to *n*_1_/*n*_2_, except only a subset of signature transcripts are utilized in the calculation [31]. This subset included only those signature transcripts that were not signature transcripts for any other cell population with a larger *n*_1_/*n*_2_ ratio (see Figure 2 and Methods from [31]). Significant evidence for infiltration or expansion of a cell population with age was present if both *n*_1_/*n*_2_ and *n*_1*_/*n*_2*_ were significantly large (FDR-adjusted P<0.05; Fisher's Exact test). P-values generated among all cell populations were adjusted using the Benjamini-Hochberg procedure [73].

### Motif analyses

Human and mouse genome coordinates were obtained from refGene files downloaded from the UCSC database (human version hg19, GRCh37, mouse version mm9). Genomic sequences were derived from Bioconductor BSgenome packages (BSgenome.Hsapiens.UCSC.hg19 and BSgenome.Mmusculus.UCSC.mm9). Position weight matrices (PWMs) were obtained from the UniPROBE and Jaspar databases [32]–[34]. For each nucleotide position within a PWM, ratios of posterior to prior probabilities were calculated using empirically estimated nucleotide background frequencies (see Table S3). Input sequences were scored for matches to a given PWM by summing log_2_-transformed (posterior/prior) probability ratios across nucleotide positions within the PWM, and a match was called for sequence regions with scores exceeding 90% of the maximum score for the PWM [82]. If overlapping sequence matches to a given motif were identified, such overlapping matches were not double-counted but were instead counted as a single match in all genome scans.

Initial screens considered PWM matches only within genomic regions 2000 bp upstream and 200 bp downstream of annotated transcription start sites (Figures S3, S4, 7 and S15). Based on results of these initial screens, a selected subset of top-ranking motifs were further investigated with respect to all intergenic, all intronic and conserved sequence regions 2000 bp upstream of the TSS for each gene (Zeb1, AP-2 and YY1 motifs in human; Zeb1 and Prrx2 motifs in mouse; Figures S6 and S17). For human, we screened a total of 19,494 intergenic regions and 191,912 intronic regions for matches to the selected PWMs (irrespective of sequence conservation). Likewise, for mouse we screened a total of 20,079 intergenic regions and 179,146 intronic regions (irrespective of sequence conservation). Human conserved sequences (≤2000 bp) upstream of transcription start sites were obtained from UCSC multiple alignments of 45 vertebrate genomes (hg19, GRCh37). Mouse conserved sequences (≤2000 bp) upstream of transcription start sites were obtained from UCSC multiple alignments of 29 vertebrate genomes (mm9). For analyses of intergenic, intronic and conserved sequences (Figures S6 and S17), sequence lengths were variable and we noted a correlation between PWM match counts and sequence length. To remove this effect, we calculated a length-adjusted count score for each sequence, which was proportional to the density of PWM matches within a given sequence. Length-adjusted scores were calculated as the residual values in a Poisson regression model, with PWM counts as the response variable and sequence length (ln-transformed) as a single predictor variable. Average length-adjusted scores were then compared between foreground and background sequence sets as described in Figure S6 and S17 legends.

## Supporting Information

Figure S1 **Ranked lists of genes altered by aging in skin and other human tissues.** Tables display age-associated expression patterns for ranked lists of the top 50 genes (A) increased by aging across all human tissues, (B) decreased by aging across all human tissues, (C) increased by aging in human skin, or (D) decreased by aging in human skin. For a given gene (row) and tissue (column), colors denote the estimated fold-change expression ratio (old/young) between an individual *t*+40 years of age and an individual *t* years of age (a linear rate of change with age is assumed). Filled triangles indicate whether genes were significantly increased or decreased by aging (P<0.05). Tables (A) and (B) list genes most frequently regulated by aging across all human tissues, with genes ranked according to the total number of significant results obtained across all columns in each table (i.e., the total number of up or down triangles per row). Genes for which expression is similarly altered in human skin are indicated by an asterisk symbol. Tables (C) and (D) list genes most strongly regulated by aging in skin specifically, with genes first filtered to include only genes significantly altered by age in both sexes (P<0.05 in each sex), and then ranked according to the estimated 40-year fold-change (i.e., old/young; averaged between males and females). Genes for which expression is similarly altered in three or more other human aging profiles are indicated by an asterisk symbol.
(PDF)

Figure S2 **Gene ontology biological process terms associated with human skin aging and their association with aging across human tissues.** Genes regulated by aging in human skin were analyzed to identify significantly over-represented gene ontology (GO) biological process terms (P<0.05; Fisher's exact test). The analysis was performed based upon (A) a set of 2469 human genes increased by aging in female skin (P<0.05), (B) a set of 2578 human genes decreased by aging in female skin (P<0.05), (C) a set of 2199 human genes increased by aging in male skin and (D) a set of 1113 human genes decreased by aging in male skin (P<0.05). The font color of each GO term in the left margin corresponds to the degree to which that term was overrepresented (P<0.05, black font; P<0.01, blue font; P<0.001, red font). For each GO term, colors within the chart denote the average 40-year fold change (old/young) among genes associated with that GO term. The number of genes associated with each GO term is indicated in brackets (this total only includes genes that are among the 2469, 2578, 2199 or 1113 genes analyzed in parts A, B, C and D, respectively). Filled triangles indicate whether average fold-change estimates among these genes are significantly high (up-triangle; P<0.05) or significantly low (down-triangle; P<0.05). Triangles are displayed only if significant p-values were obtained from each of three separate tests evaluating whether the average fold-change estimate (or distribution of estimates) is significantly high or low (i.e., one-sample t-test, exact binomial proportion test and Wilcoxon signed rank test; triangles denote P<0.05 in each test). GO terms have been clustered based upon a similarity metric that is proportional to the number of shared ancestral terms in the GO hierarchy.
(PDF)

Figure S3 **Sex-specific associations of transcription factor binding site motifs with the gene expression response to aging in human skin.** The chart lists top-scoring motifs associated with genes (A) increased by aging in female subjects, (B) decreased by aging in female subjects and (C) increased by aging in male subjects. A total of 541 position weight matrices (PWMs) associated with mammalian transcription factors were obtained from the Jaspar and UniPROBE databases. For each PWM, regions proximal to the annotated transcription start site (TSS) of known human genes were scanned to identify putative binding sites (2000 BP upstream, 200 BP downstream). Based on results from this scan, we identified genes associated with varying numbers of binding sites (1…*n*) for a given motif. The figure provides a “threshold-free” display of trends relating binding site abundance to age-associated gene expression patterns. We derived a series of gene sets (*x*_1_, *x*_2_… *x_n_*), where the first set *x*_1_ contained genes with at least one binding site in the search region, set *x*_2_ contained genes with at least two binding sites, and so on, with set *x_n_* containing genes with at least *n* binding sites. The number of genes belonging to each set declined successively in the series spanning set *x*_1_ to *x_n_*. The number of sets evaluated in this fashion (*n*) varied among the 541 PWMs considered, since more complex PWM models had fewer genome matches within search regions. For each PWM and each gene set, the proportion of genes increased by aging in females (A and B) or males (C) was evaluated (horizontal axis). For sets *x*_1_, *x*_2_,…, *x_n_*, this proportion is represented by open circles, with blue circles representing sets for which the ratio of age-increased to age-decreased genes is significantly different from 0.50 (P<0.05; Fisher's exact test), and yellow circles representing sets for which the ratio is significant at a more stringent threshold (FDR-adjusted P<0.05; Benjamini-Hochberg method). Circle size corresponds to the number of genes included in each set (see legend in right margin), where larger symbols denote larger sets of genes with fewer PWM motif matches, and smaller circles denote smaller sets of genes with more PWM motif matches. In parts (A)–(C), PWMs have been ranked according to the most extreme ratio (i.e., most different from 0.50) obtained for any set including at least 100 genes. For each age-associated motif, candidate target genes are listed in the right margin (red and green font). Candidate target genes were identified as those strongly altered by aging (P<0.05), with a maximal number of motif occurrences within the search region (age-increased genes in red font; age-decreased genes in green font). We noted that two top-scoring motifs (blue font, left margin) exhibited a similar relationship with aging in female versus male skin, while for one motif (magenta font, left margin) there was a contrasting relationship with aging in females versus males. Motifs associated with age-decreased expression in males are not shown because no gene sets with ratios (no. age-increased genes/no. age-decreased genes) significantly less than 0.50 were identified in males.
(TIF)

Figure S4 **Sex-specific associations of transcription factor binding site motifs with the gene expression response to aging in human skin.** This figure is identical to Figure S3, except a simulation-based correction for multiple testing has been applied (yellow symbols). For each simulation trial, genes were randomly assigned to gene sets (631 sets in total), where the respective size of gene sets was matched to those evaluated in our analysis. Following this random assignment of genes to gene sets, p-values were generated from each set to test for age-biased expression (Fisher's Exact Test), and the lowest p-value arising among all gene sets was identified. This procedure was repeated in 2000 simulations, yielding a distribution for the minimum p-value expected to arise by chance alone, given the total number of gene sets evaluated for age-biased expression. We then identified the 0.05 quantile of this distribution (P*), which corresponds to the p-value that is lower than the minimal p-value that arose in 95% of the simulation trials. Those gene sets associated with p-values less than P* are denoted by yellow symbols.
(TIF)

Figure S5 **Zeb1, AP-2 and YY1 binding site density near the TSS is associated with decreased gene expression with age in females but not males.** In each figure, blue or black symbols represent the average 40-year fold-change (old/young) among genes with at least *t* binding sites in the region proximal to the annotated transcription state site (2KB upstream and 200 BP downstream of the TSS). An increasing series of thresholds (*t*) was used to define gene sets of successively smaller sizes (horizontal axis), and for each set, the average 40-year fold-change was calculated (vertical axis). The background color (red or green) reflects the proportion of age-increased (red) to age-decreased genes (green) with respect to a given threshold (*t*), where an increase in the size of the red region denotes a higher proportion of age-increased genes with increasing *t*, and an increase in the size of the green region denotes a higher proportion of age-decreased genes with increasing *t*. For any given threshold *t*, blue symbols denote gene sets for which the average 40-year fold-change among genes with at least *t* binding sites is significantly different from that of genes with fewer than *t* binding sites (P<0.05; two-sample t-test). The dotted horizontal line represents the average 40-year fold-change (old/young) among all 18,442 human genes included in the analysis.
(TIF)

Figure S6 **Genomic regions with increased AP-2 binding site density are associated with decreased gene expression with age in female human subjects but not male subjects.** These analyses were performed with respect to all human non-coding intergenic regions (repeat-masked) (parts A–D), all intronic regions (repeat-masked) (parts E–H), and conserved sequences located 2000 BP upstream of the annotated TSS for all human genes (parts I–L). In each case, sequences were scanned to identify putative AP-2 binding sites (consensus motif: GCC----G), and length-adjusted residual scores were calculated for all sequences (see Methods). Positive scores indicate a large number of AP-2 binding sites (given sequence length) and negative scores indicate a small number of AP-2 binding sets (given sequence length). Average residual scores were then compared between a foreground set of sequences (adjacent to age-increased or age-decreased genes) and a background set of sequences (i.e., all other sequences excluding those in the foreground). The central tendency of the foreground sequence residual scores is displayed for (A, E, I) sequences within or adjacent to age-increased genes (red lines) in females, (B, F, J) sequences within or adjacent to age-decreased genes in females (green lines), (C, G, K) sequences within or adjacent to age-increased genes (red lines) in males, and (D, H, L) sequences within or adjacent to age-decreased genes in males (green lines). In each case, red or green lines represent the average residual score among foreground sequences (middle line) along with 95% confidence interval bounds (lower and upper lines). The grey region outlines a 95% confidence interval for the residual scores among sequences in the background set. Significant differences between the average foreground and background residual score are noted by blue bars near the top or bottom of each figure. Analyses were repeated using foreground sequence sets of varying size and selectivity (see horizontal axis). The smallest and most selective foreground set (leftmost-side of each figure) included only sequences adjacent to genes strongly altered by aging in the appropriate direction (based upon ranked p-values obtained from tests for an aging effect). The largest and least selective foreground set (rightmost-side of each figure) included sequences that were altered by aging in the appropriate direction, but were not necessarily adjacent to genes strongly altered by aging (based upon ranked p-values obtained from tests for an aging effect).
(PDF)

Figure S7 **Weak association between aging effects in sun-protected human skin and tail skin from CB6F1 mice (adjusted residuals).** A set of 15,203 human-mouse orthologous transcript pairs were placed into one of nine categories (horizontal axis in each panel) depending on whether transcripts were increased by aging (P<0.05; red up-triangles), decreased by aging (P<0.05; green down-triangles) or not altered by aging (black dash) in human sun-protected skin and/or tail skin from CB6F1 mice. For each category, an adjusted residual was calculated (vertical axis), which reflects departure from random association between human and mouse differential expression signatures (see Swindell [11]). Positive residuals indicate an overabundance of human-mouse transcript pairs within a particular category, while negative residuals indicate underabundance of human-mouse transcript pairs within a category. Under the null hypothesis (random association between human and mouse differential expression results), adjusted residuals are expected to follow a standard normal distribution, with residuals larger than three in absolute value providing evidence for a significant association between species. Correspondence between human-mouse aging patterns would have been supported by positive residuals for transcript pairs increased or decreased in both species (i.e., the fourth and third categories from the right), or alternatively, by negative residuals for transcript pairs with conflicting aging effects in the two species (i.e., the last two categories on the right).
(TIF)

Figure S8 **Weak association between aging effects in sun-protected human skin and tail skin from CB6F1 mice (AUC statistics).** 15,203 mouse transcripts were ranked according to the strength of aging effects among orthologous human genes in sun-protected skin (horizontal axis). This ranking was based upon (A) the degree of increase with aging in human females, (B) the degree of decrease with aging in human females, (C) the degree of increase with aging in human males or (D) the degree of decrease with aging in human males. In each panel (A)–(D), mouse transcripts associated with the strongest aging effect in humans (with respect to the human orthologue) were assigned lower ranks. Foreground sets of 200 mouse transcripts were then identified as those most strongly (A) increased with aging in mouse females, (B) decreased with aging in mouse females, (C) increased with aging in mouse males and (D) decreased with aging in mouse males. Red or green curves track the cumulative overlap (vertical axis) between the 200 foreground transcripts and the ranked set of 15,203 mouse transcripts. Enrichment of foreground genes with respect to the ranked lists (horizontal axis) is supported by red or green curves above the diagonal line, with a large positive area between the colored line and the diagonal (i.e., the region shaded in grey). This area is proportional to the Wilcoxon-Mann-Whitney (WMW) statistic, and for a large set of foreground genes chosen at random, is expected to follow a standard normal distribution (see equations 5–7 from Philippakis et al. [35]). The standard normal was therefore used as a null distribution to evaluate the significance of WMW statistics obtained in (A)–(D) and to generate the p-value listed in the lower-right of each panel.
(TIF)

Figure S9 **Weak association between aging effects in sun-protected human skin and tail skin from CB6F1 mice (Gene Set Enrichment Analysis).** 15,203 mouse transcripts were ranked according to the strength of aging effects among orthologous human genes in sun-protected skin (horizontal axis). This ranking was based upon (A) the degree of increase with aging in human females, (B) the degree of decrease with aging in human females, (C) the degree of increase with aging in human males or (D) the degree of decrease with aging in human males. In each panel (A)–(D), mouse transcripts associated with the strongest aging effect in humans (with respect to the human orthologue) were assigned lower ranks. Foreground sets of 200 mouse transcripts were then identified as those most strongly (A) increased with aging in mouse females, (B) decreased with aging in mouse females, (C) increased with aging in mouse males and (D) decreased with aging in mouse males. The enrichment (vertical axis) is a measure of the cumulative overlap between the foreground set of mouse transcripts and the ranked list of 15,203 mouse transcripts (see Subramanian et al. [36]). The enrichment score (ES) metric is the maximum enrichment observed across all ranked genes (see red circle) and is used as the test statistic. The null distribution of ES was generated from 1000 simulation trials in which the 15,203 mouse transcripts were ranked based upon randomized expression data. The shaded grey region within each panel represents the lower 95% of the simulated null distribution for ES. Correspondence between human-mouse aging patterns is not supported in (A)–(D) because the red circle (ES) shown in each panel lies within the grey region (i.e., within the lower 95% of the ES null distribution).
(TIF)

Figure S10 **Ranked lists of genes altered by aging in mouse tail skin and other tissues.** Tables display age-associated expression patterns for ranked lists of the top 50 genes (A) increased by aging across all mouse tissues, (B) decreased by aging across all mouse tissues, (C) increased by aging in mouse tail skin (CB6F1 strain), or (D) decreased by aging in mouse tail skin (CB6F1 strain). For a given gene (row) and tissue (column), colors denote the estimated fold-change expression ratio between an older mouse *t*+2 years of age and a younger mouse *t* years of age (a linear rate of change with age is assumed). Filled triangles denote significant aging effects (P<0.05). In (A) and (B), gene rankings are based upon the total number of significant results observed across all columns in each table, and an asterisk symbol is used to denote genes similarly altered between mouse skin (males and/or females) and the other aging profiles. In (C) and (D), genes were first filtered to include only those significantly altered by aging in skin (P<0.05), and then ranked according to fold-change estimates (old/young) (averaged between sexes). Genes for which expression is similarly altered in three or more other mouse aging profiles are indicated by an asterisk symbol.
(PDF)

Figure S11 **Gene ontology biological process terms associated with mouse tail skin aging (CB6F1 strain).** Genes regulated by aging in mouse tail skin were analyzed to identify significantly over-represented gene ontology (GO) biological process terms (P<0.05; Fisher's exact test). The analysis was performed based upon (A) a set of 2633 mouse genes significantly increased by aging in females (P<0.05), (B) a set of 3134 mouse genes significantly decreased by aging in females (P<0.05), (C) a set of 2509 mouse genes significantly increased by aging in males (P<0.05) and (D) a set of 3208 mouse genes significantly decreased by aging in males (P<0.05). The font color of each GO term in the left margin corresponds to the degree to which that term was overrepresented (P<0.05, black font; P<0.01, blue font; P<0.001, red font). For each GO term, colors within the chart denote the average 2-year fold change (old/young) among genes associated with that GO term. The number of genes associated with each GO term is indicated in brackets (this total only includes genes that are among the 2633, 3134, 2509 or 3208 genes analyzed in parts A, B, C and D, respectively). Filled triangles indicate whether average fold-change estimates among these genes are significantly high (up-triangle; P<0.05) or significantly low (down-triangle; P<0.05). Triangles are displayed only if significant p-values were obtained from each of three separate tests evaluating whether the average fold-change estimate (or distribution of estimates) is significantly high or low (i.e., one-sample t-test, exact binomial proportion test and Wilcoxon signed rank test; triangles denote P<0.05 in each test). GO terms have been clustered based upon a similarity metric that is proportional to the number of shared ancestral terms in the GO hierarchy.
(PDF)

Figure S12 ***Lyz1*** **expression is not elevated with age in tail skin but increased by age in ear skin, heart and kidney from CB6F1 mice.** RT-PCR was used to evaluate the expression of *Lyz1* in mice from three age groups (5 months, 17 months and 30 months) and from both sexes (*n* = 5–6 mice for each sex/age combination). The expression of 18S ribosomal RNA (Rn18s) was used as an internal control gene for calculation of relative expression in each sample. Plots (A)–(F) show mean expression levels in each group (± one standard error) normalized to the average expression level of the young (5 month) female group. In each plot, and for each sex, the average expression of middle-aged (17 month) and old groups (30 months) was compared to that of the young group (5 months). A red star indicates a significant difference relative to the youngest group of the same sex (P<0.05; two-sided two-sample t-test). A blue star is used to indicate a significant difference based upon a two-sample non-parametric statistical test (P<0.05; either Wilcoxon or Kruskal-Wallis rank sum test).
(TIF)

Figure S13 ***Lyz2*** **expression is not elevated with age in tail skin but increased by age in ear skin, heart, kidney and small intestine from CB6F1 mice.** RT-PCR was used to evaluate the expression of *Lyz2* in mice from three age groups (5 months, 17 months and 30 months) and from both sexes (*n* = 5–6 mice for each sex/age combination). The expression of 18S ribosomal RNA (Rn18s) was used as an internal control gene for calculation of relative expression in each sample. Plots (A)–(F) show mean expression levels in each group (± one standard error) normalized to the average expression level of the young (5 month) female group. In each plot, and for each sex, the average expression of middle-aged (17 month) and old groups (30 months) was compared to that of the young group (5 months). A red star indicates a significant difference relative to the youngest group of the same sex (P<0.05; two-sided two-sample t-test). A blue star is used to indicate a significant difference based upon a two-sample non-parametric statistical test (P<0.05; either Wilcoxon or Kruskal-Wallis rank sum test).
(TIF)

Figure S14 **Aging of mouse tail skin leads to decreased expression of transcripts associated with dendritic cells but increased expression of CD4+ T cell-associated genes (CB6F1 strain).** We identified 500 probe sets (from the Affymetrix Mouse Genome 430 2.0 Array) associated with signature transcripts that exhibit high expression in CD4+ T-cells isolated from spleen (based upon data provided under GEO series accession GSE20366). Likewise, we identified 500 probe sets associated with transcripts that exhibit high expression in dendritic cells harvested from lung tissue (GSE18607). The scatterplots (A)–(D) display expression of these transcripts in tail skin samples from young (5 month) and old (30 month) CB6F1 mice of both sexes (*n* = 5 per age group for each sex). In each scatterplot, red symbols represent probe sets with higher expression in old mice, while green symbols represent probe sets with higher expression in young mice. The relative proportion of these two probe set groups is indicated by the pie chart shown in each panel.
(TIF)

Figure S15 **Top-ranked motifs associated with aging effects in mouse tail skin (CB6F1 strain): Overlap with top-ranked human motifs but dissimilar associations with aging.** This figure is identical to Figure 8, except a simulation-based correction for multiple testing has been applied (yellow symbols). For each simulation trial, genes were randomly assigned to gene sets (625 sets in total), where the respective size of gene sets was matched to those evaluated in our analysis. Following this random assignment of genes to gene sets, p-values were generated from each set to test for age-biased expression (Fisher's Exact Test), and the lowest p-value arising among all gene sets was identified. This procedure was repeated in 2000 simulations, yielding a distribution for the minimum p-value expected to arise by chance alone, given the total number of gene sets evaluated for age-biased expression. We then identified the 0.05 quantile of this distribution (P*), which corresponds to the p-value that is lower than the minimal p-value that arose in 95% of the simulation trials. Those gene sets associated with p-values less than P* are denoted by yellow symbols.
(TIF)

Figure S16 **Zeb1 and Prrx2 motif density near the TSS is associated with age-related expression patterns in tail skin from both sexes (CB6F1 strain).** In each figure, blue or black symbols represent the average 2-year fold-change (old/young) among genes with at least *t* binding sites in the region proximal to the annotated transcription start site (2 KB upstream and 200 BP downstream of the TSS). An increasing series of thresholds (*t*) was used to define gene sets of successively smaller sizes (horizontal axis), and for each set, the average 2-year fold-change was calculated (vertical axis). The background color (red or green) reflects the proportion of age-increased (red) to age-decreased genes (green) with respect to a given threshold (*t*), where an increase in the size of the red region denotes a higher proportion of age-increased genes, and an increase in the size of the green region denotes a higher proportion of age-decreased genes. For any given threshold *t*, blue symbols denote gene sets for which the average 2-year fold-change among genes with at least *t* binding sites is significantly different from that of genes with fewer than *t* binding sites (P<0.05; two-sample t-test). The dotted horizontal line represents the average 2-year fold-change (old/young) among all 17, 329 mouse genes included in the analysis.
(TIF)

Figure S17 **Genomic regions with increased Zeb1 motif density are associated with increased gene expression with aging of tail skin from both sexes (CB6F1 strain).** The panels (A)–(L) compare Zeb1 motif density between sequences adjacent to or within genes exhibiting increased (or decreased) expression with age (i.e., foreground sequences), relative to sequences adjacent to or within genes that exhibit decreased (or increased) expression with age (i.e., background sequences) (i.e., as described in the AP-2 analysis shown in Figure S6). For each sequence examined, a length-adjusted residual score was calculated, which is proportional to the density of Zeb1binding sites within that sequence. Each panel shows the average residual score among sequences adjacent to or within age-increased genes (see red lines and 95% confidence intervals in A, C, E, G, I and K) or the average residual score among sequences adjacent to or within age-decreased genes (see green lines and 95% confidence intervals in B, D, F, H, J and L). The grey region in each panel outlines a 95% confidence interval for the average residual score among sequences assigned to the background set (which includes all sequences not within the foreground set). The analysis was repeated based upon foreground sets of varying size and selectivity (horizontal axis; see Figure S6 legend). A significant motif-expression association is indicated by non-overlap between 95% confidence intervals associated with foreground and background gene sets (denoted by blue bars located near the top or bottom of each panel).
(PDF)

Figure S18 **Ranked lists of genes altered by aging in both human and mouse tissues.** Tables display age-associated expression patterns for ranked lists of the top 50 genes most strongly (A) increased by aging across all human tissues, (B) decreased by aging across all human tissues, (C) increased by aging in human skin (both sexes), (D) decreased by aging in human skin (both sexes), (E) increased by aging across all mouse tissues, (F) decreased by aging across all mouse tissues, (G) increased by aging in mouse skin (both sexes) and (H) decreased by aging in mouse skin (both sexes). In each figure (A)–(H), age-associated gene expression patterns in human tissues are displayed in the left panel, and age-associated gene expression patterns in mouse tissues are displayed in the right panel. Ranked lists include only orthologous genes that are shared between humans and mice (and thus may differ from those presented in Figures S1 and S10). In (A), (B), (E) and (F), genes have been ranked according to the total number of significant results observed for a given gene across human and mouse tissues (i.e., the total number of up-triangles per row or the total number of down-triangles per row). Tables (C), (D), (G) and (H) list genes most strongly regulated by aging in skin specifically, with genes first filtered to include only genes significantly altered by age in both sexes of a given species (P<0.05), and then ranked according to the estimated 40-year or 2-year fold-change (old/young; averaged between males and females).
(PDF)

Figure S19 **Biological processes associated with concordant and discordant age-associated expression patterns in human sun-protected skin and mouse tail skin.** Figures (A) and (B) list gene ontology (GO) biological process terms significantly overrepresented (P<0.05) among genes with (A) concordant or (B) discordant age-associated expression patterns in humans and mice. The analysis was performed based upon (A) 2190 genes concordantly altered by aging in humans and mice (i.e., increased or decreased in the same sex of both species; P<0.05 for each species) and (B) 644 genes altered discordantly altered by aging in humans and mice (i.e., increased in one species and decreased in the other for the same sex; P<0.05 per species). GO terms in both (A) and (B) have been clustered based upon a similarity metric that is proportional to the number of shared ancestral terms in the GO hierarchy. For each term, an exemplar gene is listed in parentheses, which exhibits either a corresponding (in part A) or non-corresponding (in part B) expression pattern in humans and mice. As an alternative strategy (Figures C–F), we scored 5242 GO biological process terms (all GO terms associated with orthologous genes in humans and mice), and identified those for which the direction of aging effects (i.e., increase or decrease) was most similar or discordant in human and mouse skin (i.e., regardless of whether genes were *significantly* altered by aging in either species; see Figures C–F). For each GO term selected in this fashion, figures (C)–(F) display the proportion of age-increased and age-decreased genes in each species (red and green bars). The right margin of Figures (C)–(F) lists three exemplar genes associated with the selected terms.
(PDF)

Figure S20 **The overlap among sets of age-regulated genes derived from different datasets with young and old tissues is characteristically 10–15%.** We calculated the pairwise overlap between sets of age-regulated genes derived from (A) 27 datasets that included young and old human tissue samples and (B) 30 datasets that included young and old mouse tissue samples. All human datasets considered in part (A) were generated from the same Affymetrix Human Genome U133 Plus 2.0 array platform (Table S1), and all mouse datasets considered in part (B) used the same Affymetrix Mouse Genome 430 2.0 Array platform (Table S2). Further details on each dataset included are provided in the Methods section. In both (A) and (B), datasets used to derive sets of age-regulated genes are listed along the horizontal axis. The height of each corresponding grey bar indicates the percentage of genes significantly altered by age (either increased or decreased), based upon a comparison-wise Type I error rate of P<0.05, such that approximately 5% of genes are expected to be age-regulated by chance (see yellow background behind grey bars). For each dataset, red and green dots indicate the percent overlap between the set of age-regulated genes identified from that dataset as compared to the others included in the same figure. Percent overlap is defined as 100× (*x*/*y*), where *x* is the number of unique genes significantly altered by age (in the same direction) in two separate datasets, and *y* is the number of unique genes significantly altered by age in the reference dataset listed along the horizontal axis. Red dots indicate overlap with respect to sets of *age-increased* genes, while green dots indicate overlap with respect to sets of *age-decreased* genes. In (A), we evaluated gene set overlap among all 351 pairwise combinations among the 27 human datasets, and on average, overlap between sets of age-regulated genes was 11.4%, with a minimum of 0.50% and a maximum of 66.5%. In (B), we evaluated gene set overlap among all 435 pairwise combinations among the 30 mouse datasets, and on average, overlap between sets of age-regulated genes was 12.5%, with a minimum of 0.40% and a maximum of 54.3%.
(PDF)

Table S1 **Human datasets (Affymetrix Human Genome U133 Plus 2.0 array).** This table lists datasets used to evaluate the effects of aging on gene expression in skin and other human tissues. The table includes experimental details for each dataset along with information on the number of genes identified as significantly altered by aging (P<0.05 and FDR-adjusted P<0.05).
(PDF)

Table S2 **Mouse Datasets (Affymetrix Mouse Genome 430 2.0 array).** This table lists datasets used to evaluate the effects of aging on gene expression in skin and other mouse tissues. The table includes experimental details for each dataset along with information on the number of genes identified as significantly altered by aging (P<0.05 and FDR-adjusted P<0.05).
(PDF)

Table S3 **Empirical estimates of nucleotide frequency background distributions (human hg19 and mouse mm9).** Human and mouse genomes were searched to identify matches with respect to position weight matrices representing motifs associated with known mammalian transcription factors. This table lists the region-specific estimates of the nucleotide frequency background distribution used for the calculation of position weight matrices.
(PDF)
