# Supplementary material for: Meta-Profiles of Gene Expression during Aging: Limited Similarities between Mouse and Human and an Unexpectedly Decreased Inflammatory Signature
Source: PLoS One. 2012 Mar 7;7(3):e33204. doi: 10.1371/journal.pone.0033204 (PMC3296693; doi:10.1371/journal.pone.0033204)
Supplement: Figure S1 — Ranked lists of genes altered by aging in skin and other human tissues. Tables display age-associated expression patterns for ranked lists of the top 50 genes (A) increased by aging across all human tissues, (B) decreased by aging across all human tissues, (C) increased by aging in human skin, or (D) decreased by aging in human skin. For a given gene (row) and tissue (column), colors denote the estimated fold-change expression ratio (old/young) between an individual t+40 years of age and an individual t years of age (a linear rate of change with age is assumed). Filled triangles indicate whether genes were significantly increased or decreased by aging (P<0.05). Tables (A) and (B) list genes most frequently regulated by aging across all human tissues, with genes ranked according to the total number of significant results obtained across all columns in each table (i.e., the total number of up or down triangles per row). Genes for which expression is similarly altered in human skin are indicated by an asterisk symbol. Tables (C) and (D) list genes most strongly regulated by aging in skin specifically, with genes first filtered to include only genes significantly altered by age in both sexes (P<0.05 in each sex), and then ranked according to the estimated 40-year fold-change (i.e., old/young; averaged between males and females). Genes for which expression is similarly altered in three or more other human aging profiles are indicated by an asterisk symbol. (PDF) [file pone.0033204.s001.pdf]

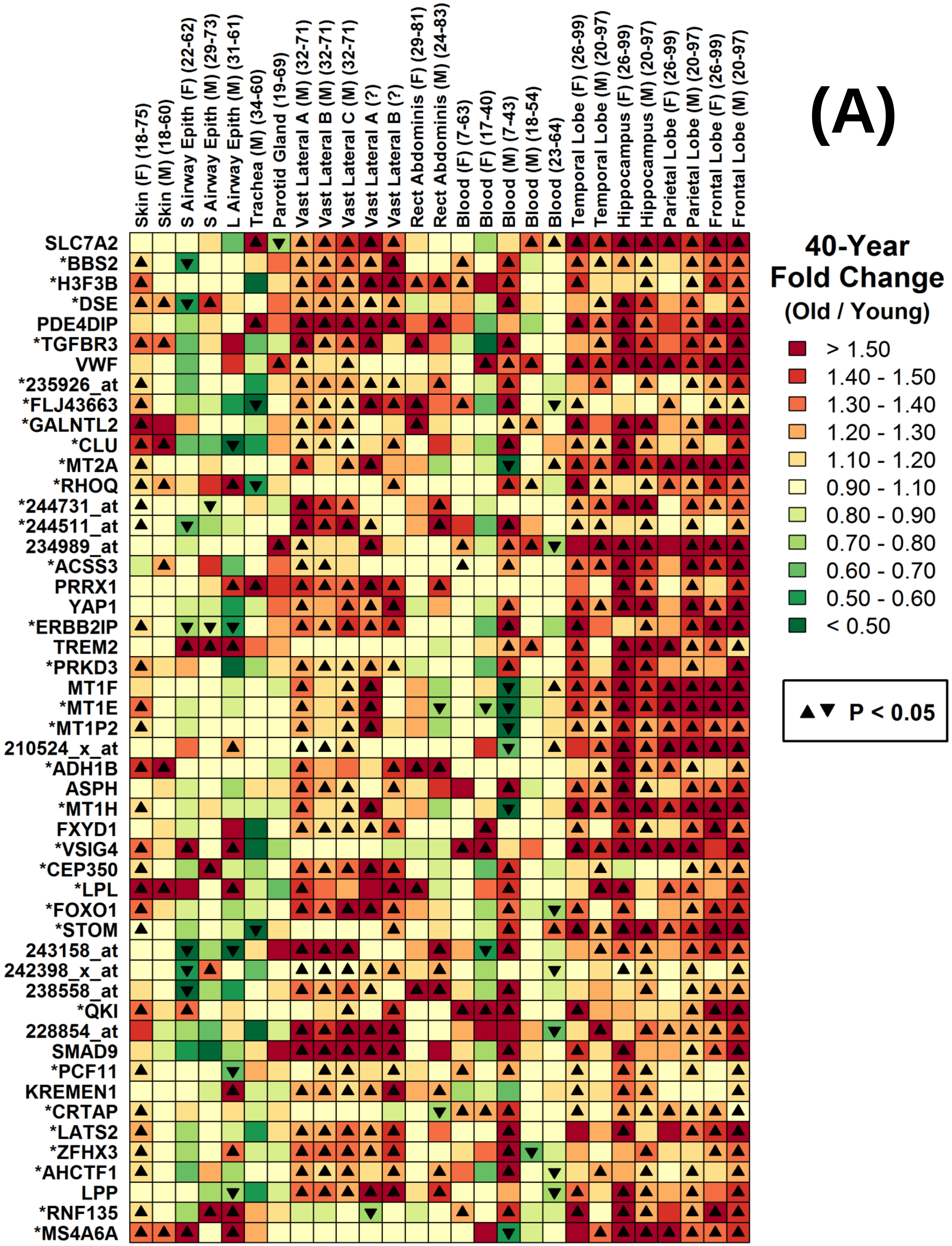
(A)
Skin (F) (18-75)
Skin (M) (18-60)
S Airway Epith (F) (22-62)
S Airway Epith (M) (29-73)
L Airway Epith (M) (31-61)
Trachea (M) (34-60)
Parotid Gland (19-69)
Vast Lateral A (M) (32-71)
Vast Lateral B (M) (32-71)
Vast Lateral C (M) (32-71)
Vast Lateral A (?)
Vast Lateral B (?)
Rect Abdominis (F) (29-81)
Rect Abdominis (M) (24-83)
Blood (F) (7-63)
Blood (F) (17-40)
Blood (M) (7-43)
Blood (M) (18-54)
Blood (23-64)
Temporal Lobe (F) (26-99)
Temporal Lobe (M) (20-97)
Hippocampus (F) (26-99)
Hippocampus (M) (20-97)
Parietal Lobe (F) (26-99)
Parietal Lobe (M) (20-97)
Frontal Lobe (F) (26-99)
Frontal Lobe (M) (20-97)
SLC7A2
*BBS2
*H3F3B
*DSE
PDE4DIP
*TGFBR3
VWF
*235926_at
*FLJ43663
*GALNTL2
*CLU
*MT2A
*RHOQ
*244731_at
*244511_at
234989_at
*ACSS3
PRRX1
YAP1
*ERBB2IP
TREM2
*PRKD3
MT1F
*MT1E
*MT1P2
210524_x_at
*ADH1B
ASPH
*MT1H
FXYD1
*VSIG4
*CEP350
*LPL
*FOXO1
*STOM
243158_at
242398_x_at
238558_at
*QKI
228854_at
SMAD9
*PCF11
KREMEN1
*CRTAP
*LATS2
*ZFHX3
*AHCTF1
LPP
*RNF135
*MS4A6A
40-Year Fold Change (Old / Young)
> 1.50
1.40 - 1.50
1.30 - 1.40
1.20 - 1.30
1.10 - 1.20
0.90 - 1.10
0.80 - 0.90
0.70 - 0.80
0.60 - 0.70
0.50 - 0.60
< 0.50
▲▼ P < 0.05

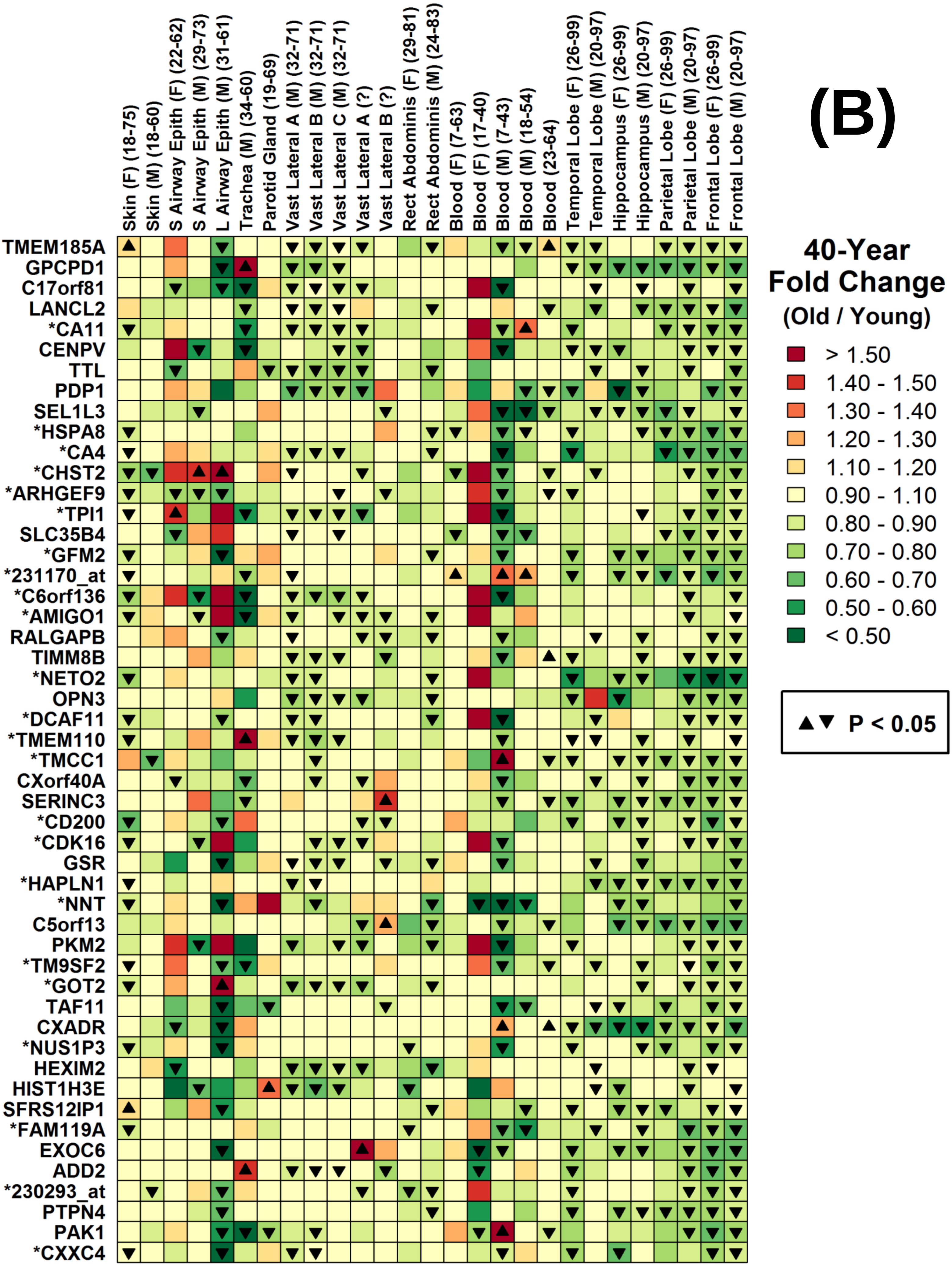

(B)
Skin (F) (18-75)
Skin (M) (18-60)
S Airway Epith (F) (22-62)
S Airway Epith (M) (29-73)
L Airway Epith (M) (31-61)
Trachea (M) (34-60)
Parotid Gland (19-69)
Vast Lateral A (M) (32-71)
Vast Lateral B (M) (32-71)
Vast Lateral C (M) (32-71)
Vast Lateral A (?)
Vast Lateral B (?)
Rect Abdominis (F) (29-81)
Rect Abdominis (M) (24-83)
Blood (F) (7-63)
Blood (F) (17-40)
Blood (M) (7-43)
Blood (M) (18-54)
Blood (23-64)
Temporal Lobe (F) (26-99)
Temporal Lobe (M) (20-97)
Hippocampus (F) (26-99)
Hippocampus (M) (20-97)
Parietal Lobe (F) (26-99)
Parietal Lobe (M) (20-97)
Frontal Lobe (F) (26-99)
Frontal Lobe (M) (20-97)
TMEM185A
GPCPD1
C17orf81
LANCL2
*CA11
CENPV
TTL
PDP1
SEL1L3
*HSPA8
*CA4
*CHST2
*ARHGEF9
*TPI1
SLC35B4
*GFM2
*231170_at
*C6orf136
*AMIGO1
RALGAPB
TIMM8B
*NETO2
OPN3
*DCAF11
*TMEM110
*TMCC1
CXorf40A
SERINC3
*CD200
*CDK16
GSR
*HAPLN1
*NNT
C5orf13
PKM2
*TM9SF2
*GOT2
TAF11
CXADR
*NUS1P3
HEXIM2
HIST1H3E
SFRS12IP1
*FAM119A
EXOC6
ADD2
*230293_at
PTPN4
PAK1
*CXXC4
40-Year Fold Change (Old / Young)
> 1.50
1.40 - 1.50
1.30 - 1.40
1.20 - 1.30
1.10 - 1.20
0.90 - 1.10
0.80 - 0.90
0.70 - 0.80
0.60 - 0.70
0.50 - 0.60
< 0.50
▲▼ P < 0.05

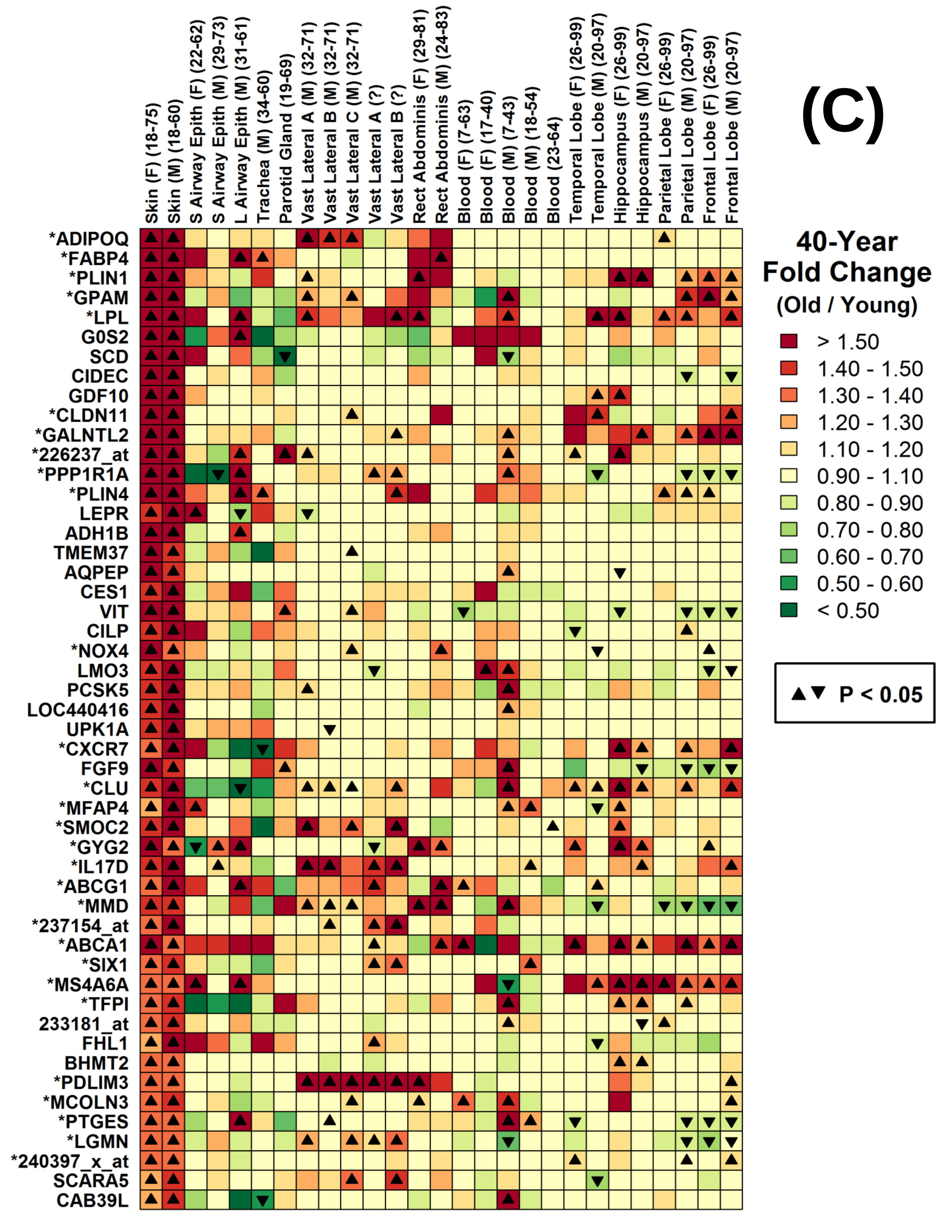

(C)
Skin (F) (18-75)
Skin (M) (18-60)
S Airway Epith (F) (22-62)
S Airway Epith (M) (29-73)
L Airway Epith (M) (31-61)
Trachea (M) (34-60)
Parotid Gland (19-69)
Vast Lateral A (M) (32-71)
Vast Lateral B (M) (32-71)
Vast Lateral C (M) (32-71)
Vast Lateral A (?)
Vast Lateral B (?)
Rect Abdominis (F) (29-81)
Rect Abdominis (M) (24-83)
Blood (F) (7-63)
Blood (F) (17-40)
Blood (M) (7-43)
Blood (M) (18-54)
Blood (23-64)
Temporal Lobe (F) (26-99)
Temporal Lobe (M) (20-97)
Hippocampus (F) (26-99)
Hippocampus (M) (20-97)
Parietal Lobe (F) (26-99)
Parietal Lobe (M) (20-97)
Frontal Lobe (F) (26-99)
Frontal Lobe (M) (20-97)
*ADIPOQ
*FABP4
*PLIN1
*GPAM
*LPL
G0S2
SCD
CIDEC
GDF10
*CLDN11
*GALNTL2
*226237_at
*PPP1R1A
*PLIN4
LEPR
ADH1B
TMEM37
AQPEP
CES1
VIT
CILP
*NOX4
LMO3
PCSK5
LOC440416
UPK1A
*CXCR7
FGF9
*CLU
*MFAP4
*SMOC2
*GYG2
*IL17D
*ABCG1
*MMD
*237154_at
*ABCA1
*SIX1
*MS4A6A
*TFPI
233181_at
FHL1
BHMT2
*PDLIM3
*MCOLN3
*PTGES
*LGMN
*240397_x_at
SCARA5
CAB39L
40-Year Fold Change (Old / Young)
> 1.50
1.40 - 1.50
1.30 - 1.40
1.20 - 1.30
1.10 - 1.20
0.90 - 1.10
0.80 - 0.90
0.70 - 0.80
0.60 - 0.70
0.50 - 0.60
< 0.50
▲▼ P < 0.05

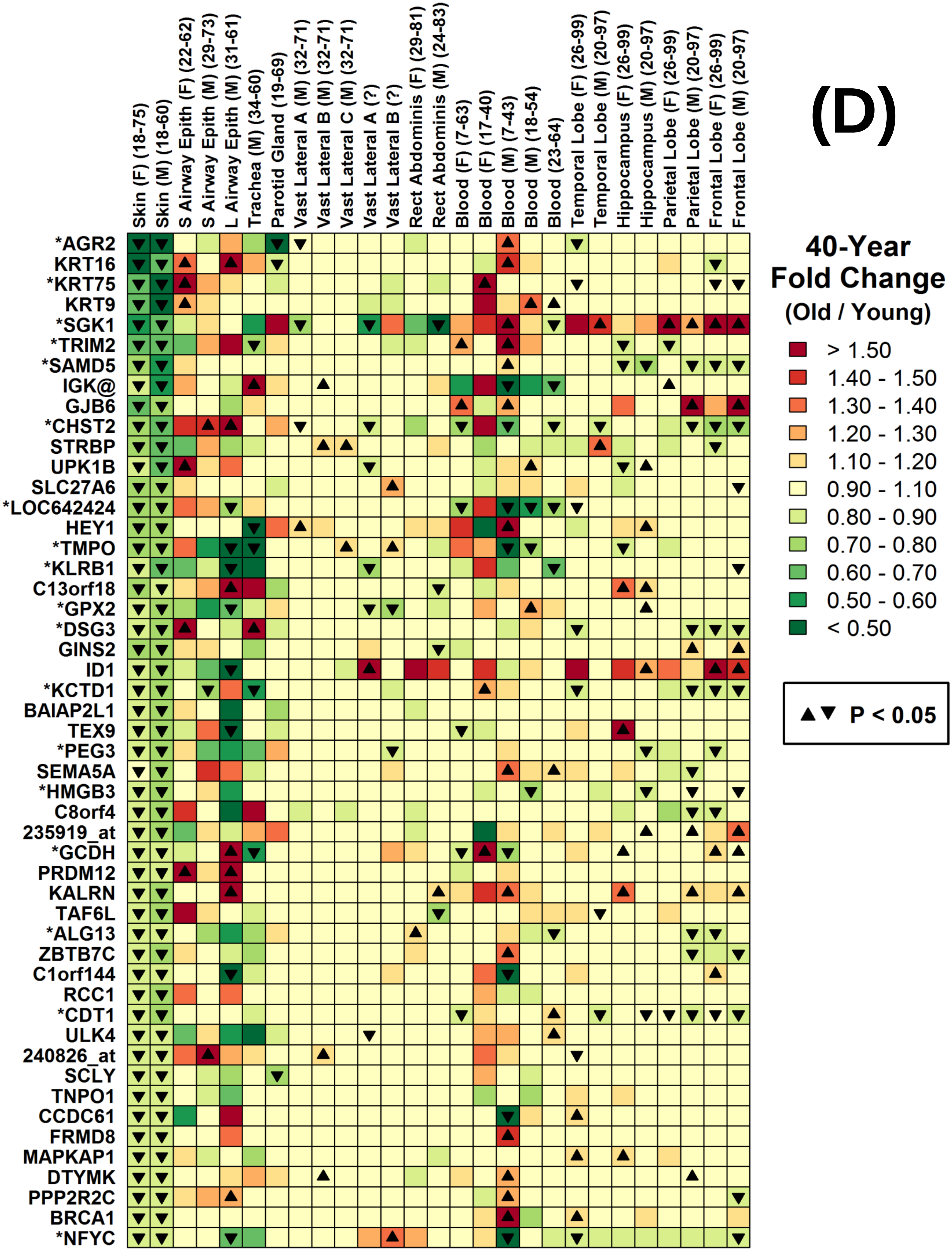

(D)
Skin (F) (18-75)
Skin (M) (18-60)
S Airway Epith (F) (22-62)
S Airway Epith (M) (29-73)
L Airway Epith (M) (31-61)
Trachea (M) (34-60)
Parotid Gland (19-69)
Vast Lateral A (M) (32-71)
Vast Lateral B (M) (32-71)
Vast Lateral C (M) (32-71)
Vast Lateral A (?)
Vast Lateral B (?)
Rect Abdominis (F) (29-81)
Rect Abdominis (M) (24-83)
Blood (F) (7-63)
Blood (F) (17-40)
Blood (M) (7-43)
Blood (M) (18-54)
Blood (23-64)
Temporal Lobe (F) (26-99)
Temporal Lobe (M) (20-97)
Hippocampus (F) (26-99)
Hippocampus (M) (20-97)
Parietal Lobe (F) (26-99)
Parietal Lobe (M) (20-97)
Frontal Lobe (F) (26-99)
Frontal Lobe (M) (20-97)
*AGR2
KRT16
*KRT75
KRT9
*SGK1
*TRIM2
*SAMD5
IGK@
GJB6
*CHST2
STRBP
UPK1B
SLC27A6
*LOC642424
HEY1
*TMPO
*KLRB1
C13orf18
*GPX2
*DSG3
GINS2
ID1
*KCTD1
BAIAP2L1
TEX9
*PEG3
SEMA5A
*HMGB3
C8orf4
235919_at
*GCDH
PRDM12
KALRN
TAF6L
*ALG13
ZBTB7C
C1orf144
RCC1
*CDT1
ULK4
240826_at
SCLY
TNPO1
CCDC61
FRMD8
MAPKAP1
DTYMK
PPP2R2C
BRCA1
*NFYC
40-Year
Fold Change
(Old / Young)
> 1.50
1.40 - 1.50
1.30 - 1.40
1.20 - 1.30
1.10 - 1.20
0.90 - 1.10
0.80 - 0.90
0.70 - 0.80
0.60 - 0.70
0.50 - 0.60
< 0.50
▲▼ P < 0.05
