# Supplementary figures and images for: Meta-Profiles of Gene Expression during Aging: Limited Similarities between Mouse and Human and an Unexpectedly Decreased Inflammatory Signature
Source: PLoS One. 2012 Mar 7;7(3):e33204. doi: 10.1371/journal.pone.0033204 (PMC3296693; doi:10.1371/journal.pone.0033204)

(A) Females  
Age-increased Genes

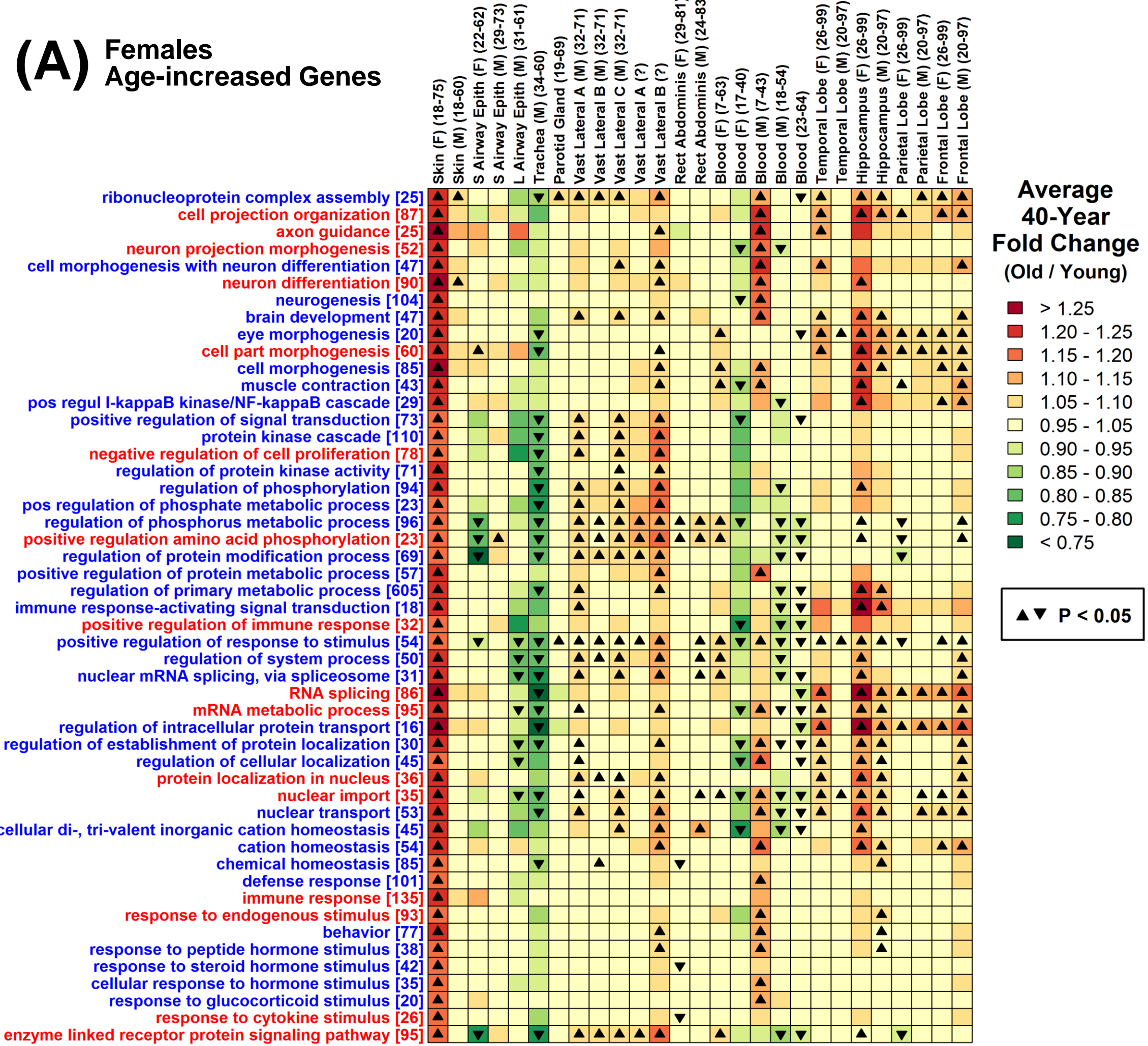

**(B)**

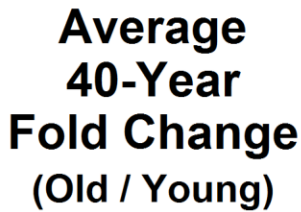

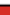 > 1.25  
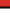 1.20 - 1.25  
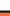 1.15 - 1.20  
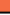 1.10 - 1.15  
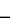 1.05 - 1.10  
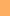 0.95 - 1.05  
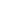 0.90 - 0.95  
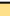 0.85 - 0.90  
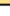 0.80 - 0.85  
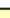 0.75 - 0.80  
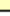 < 0.75

▲▼  $P < 0.05$

(C) Males  
Age-increased Genes

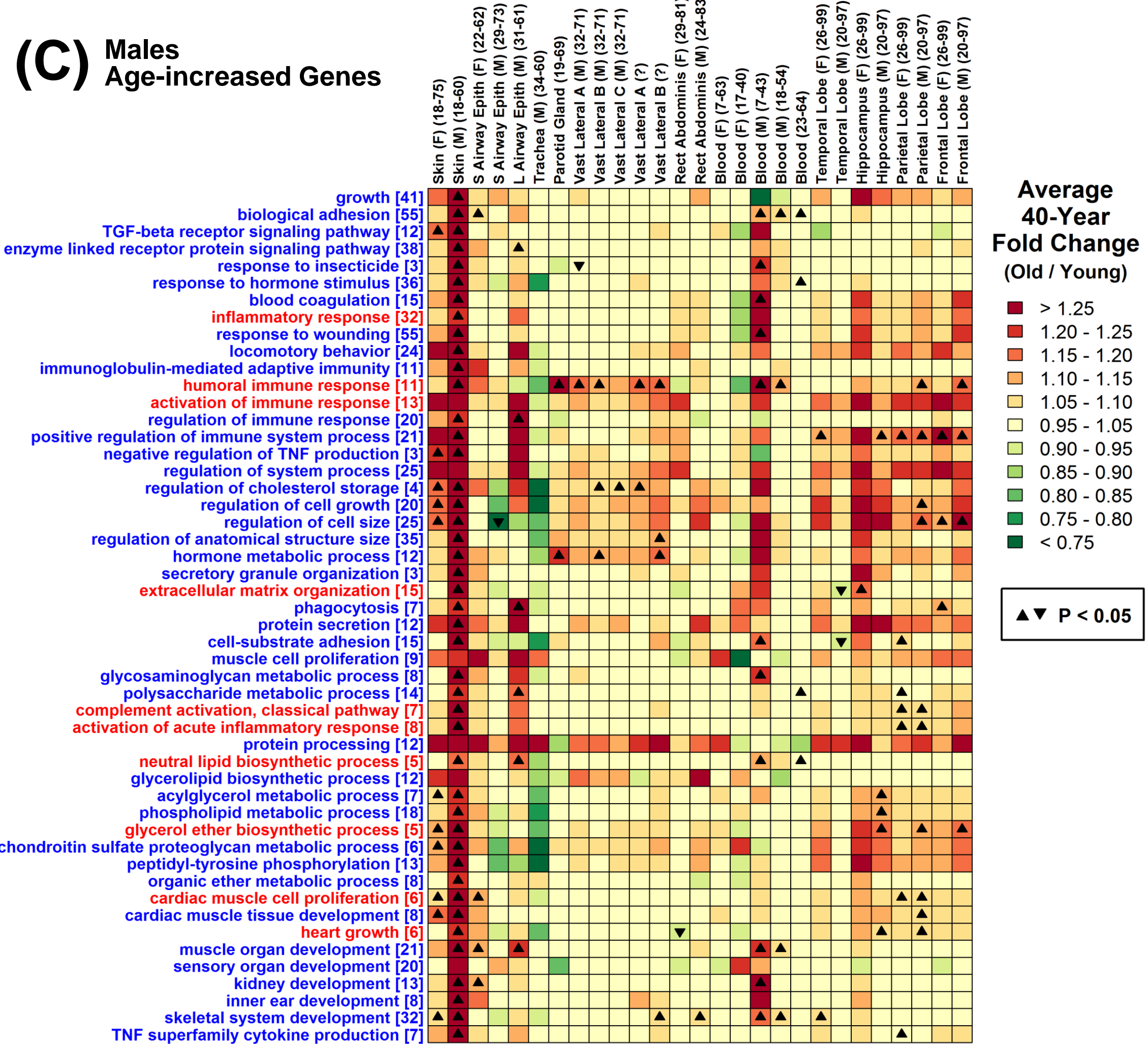

(D) Males  
Age-decreased Genes

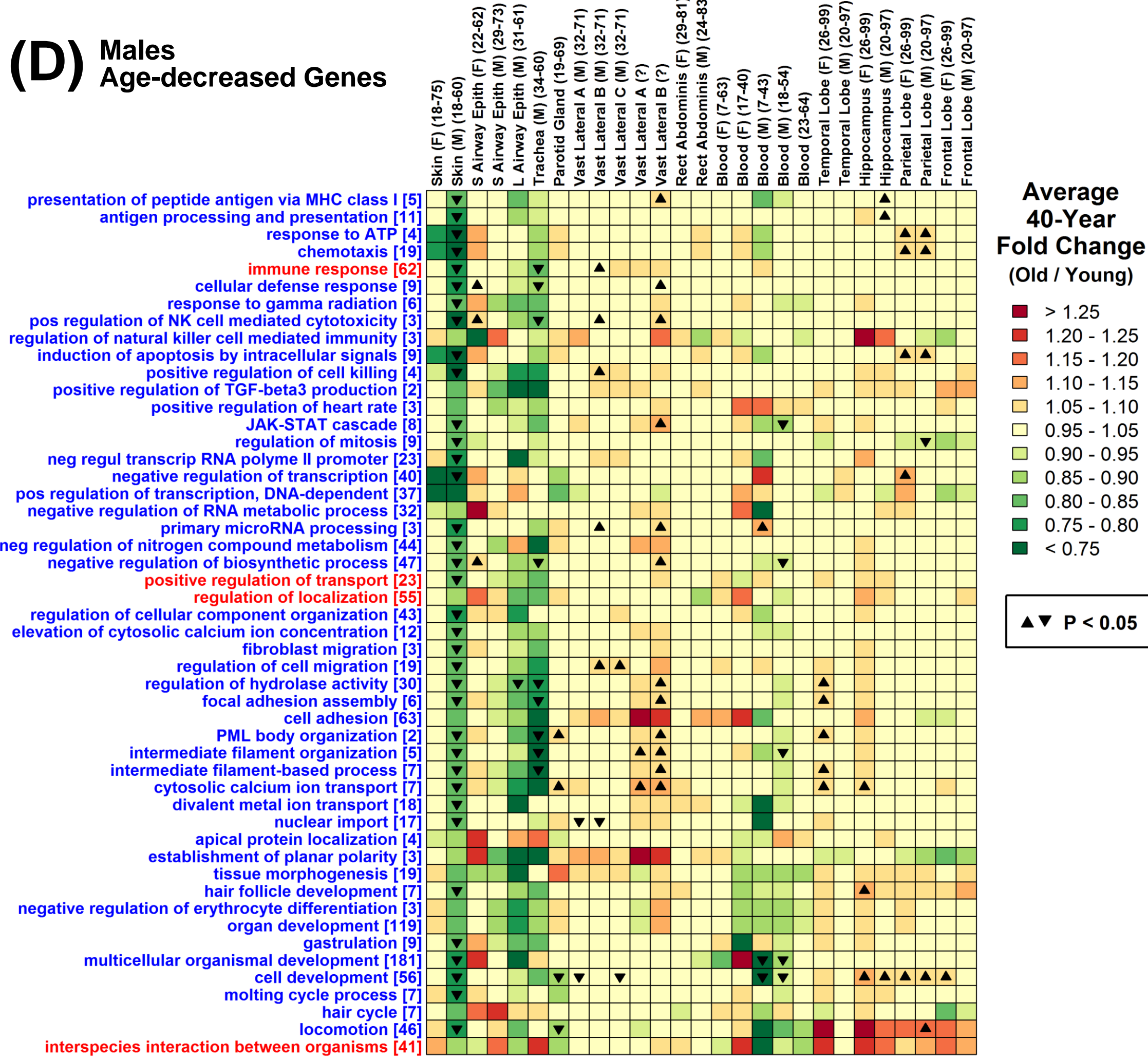

Supplement: Figure S2 — Gene ontology biological process terms associated with human skin aging and their association with aging across human tissues. Genes regulated by aging in human skin were analyzed to identify significantly over-represented gene ontology (GO) biological process terms (P<0.05; Fisher's exact test). The analysis was performed based upon (A) a set of 2469 human genes increased by aging in female skin (P<0.05), (B) a set of 2578 human genes decreased by aging in female skin (P<0.05), (C) a set of 2199 human genes increased by aging in male skin and (D) a set of 1113 human genes decreased by aging in male skin (P<0.05). The font color of each GO term in the left margin corresponds to the degree to which that term was overrepresented (P<0.05, black font; P<0.01, blue font; P<0.001, red font). For each GO term, colors within the chart denote the average 40-year fold change (old/young) among genes associated with that GO term. The number of genes associated with each GO term is indicated in brackets (this total only includes genes that are among the 2469, 2578, 2199 or 1113 genes analyzed in parts A, B, C and D, respectively). Filled triangles indicate whether average fold-change estimates among these genes are significantly high (up-triangle; P<0.05) or significantly low (down-triangle; P<0.05). Triangles are displayed only if significant p-values were obtained from each of three separate tests evaluating whether the average fold-change estimate (or distribution of estimates) is significantly high or low (i.e., one-sample t-test, exact binomial proportion test and Wilcoxon signed rank test; triangles denote P<0.05 in each test). GO terms have been clustered based upon a similarity metric that is proportional to the number of shared ancestral terms in the GO hierarchy. (PDF) [file pone.0033204.s002.pdf]

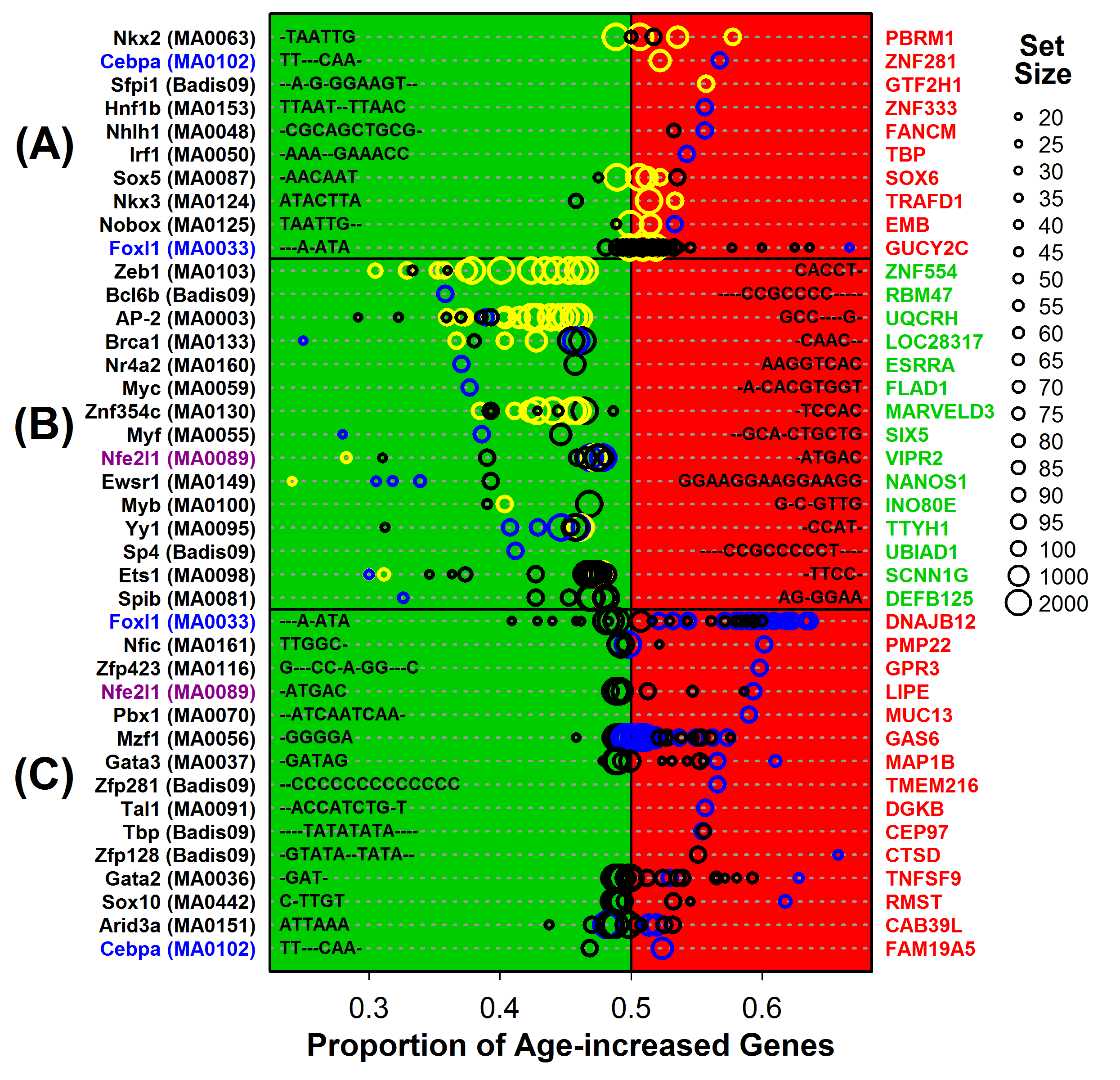

Supplement: Figure S3 — Sex-specific associations of transcription factor binding site motifs with the gene expression response to aging in human skin. The chart lists top-scoring motifs associated with genes (A) increased by aging in female subjects, (B) decreased by aging in female subjects and (C) increased by aging in male subjects. A total of 541 position weight matrices (PWMs) associated with mammalian transcription factors were obtained from the Jaspar and UniPROBE databases. For each PWM, regions proximal to the annotated transcription start site (TSS) of known human genes were scanned to identify putative binding sites (2000 BP upstream, 200 BP downstream). Based on results from this scan, we identified genes associated with varying numbers of binding sites (1…n) for a given motif. The figure provides a “threshold-free” display of trends relating binding site abundance to age-associated gene expression patterns. We derived a series of gene sets (x 1, x 2… xn), where the first set x 1 contained genes with at least one binding site in the search region, set x 2 contained genes with at least two binding sites, and so on, with set xn containing genes with at least n binding sites. The number of genes belonging to each set declined successively in the series spanning set x 1 to xn. The number of sets evaluated in this fashion (n) varied among the 541 PWMs considered, since more complex PWM models had fewer genome matches within search regions. For each PWM and each gene set, the proportion of genes increased by aging in females (A and B) or males (C) was evaluated (horizontal axis). For sets x 1, x 2,…, xn, this proportion is represented by open circles, with blue circles representing sets for which the ratio of age-increased to age-decreased genes is significantly different from 0.50 (P<0.05; Fisher's exact test), and yellow circles representing sets for which the ratio is significant at a more stringent threshold (FDR-adjusted P<0.05; Benjamini-Hochberg method). Circle size correspon [file pone.0033204.s003.tif]

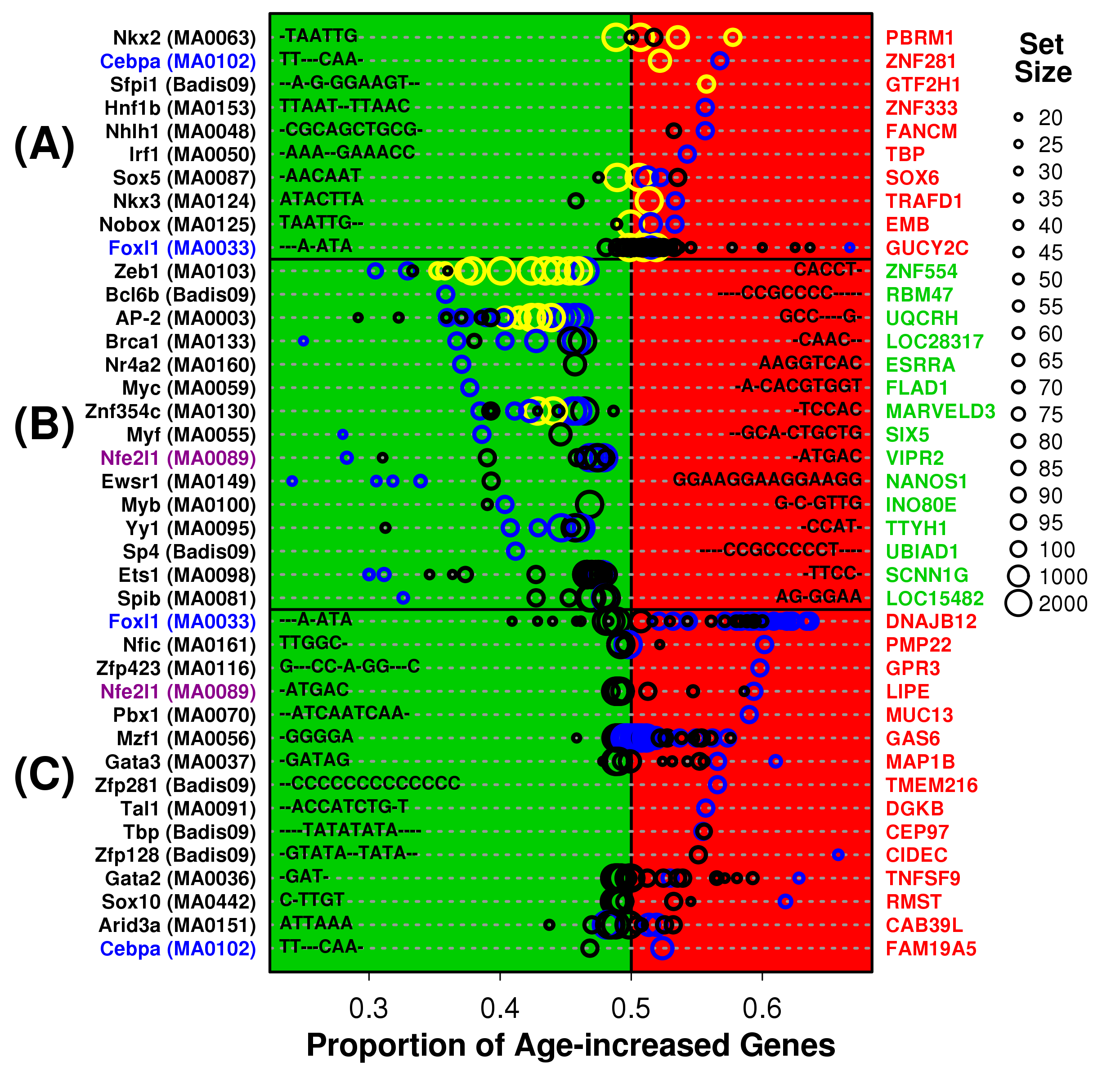

Supplement: Figure S4 — Sex-specific associations of transcription factor binding site motifs with the gene expression response to aging in human skin. This figure is identical to Figure S3, except a simulation-based correction for multiple testing has been applied (yellow symbols). For each simulation trial, genes were randomly assigned to gene sets (631 sets in total), where the respective size of gene sets was matched to those evaluated in our analysis. Following this random assignment of genes to gene sets, p-values were generated from each set to test for age-biased expression (Fisher's Exact Test), and the lowest p-value arising among all gene sets was identified. This procedure was repeated in 2000 simulations, yielding a distribution for the minimum p-value expected to arise by chance alone, given the total number of gene sets evaluated for age-biased expression. We then identified the 0.05 quantile of this distribution (P*), which corresponds to the p-value that is lower than the minimal p-value that arose in 95% of the simulation trials. Those gene sets associated with p-values less than P* are denoted by yellow symbols. (TIF) [file pone.0033204.s004.tif]

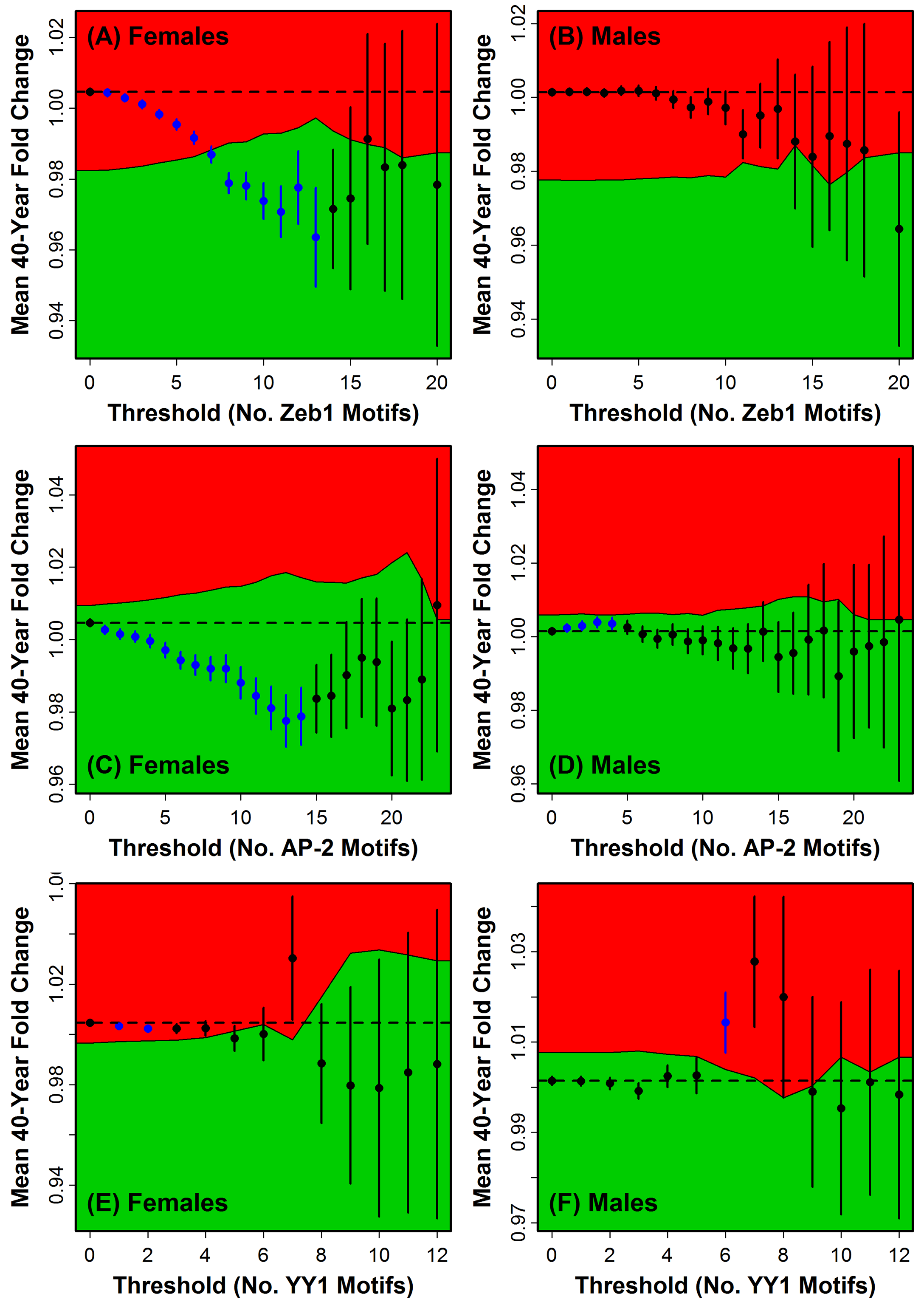

Supplement: Figure S5 — Zeb1, AP-2 and YY1 binding site density near the TSS is associated with decreased gene expression with age in females but not males. In each figure, blue or black symbols represent the average 40-year fold-change (old/young) among genes with at least t binding sites in the region proximal to the annotated transcription state site (2KB upstream and 200 BP downstream of the TSS). An increasing series of thresholds (t) was used to define gene sets of successively smaller sizes (horizontal axis), and for each set, the average 40-year fold-change was calculated (vertical axis). The background color (red or green) reflects the proportion of age-increased (red) to age-decreased genes (green) with respect to a given threshold (t), where an increase in the size of the red region denotes a higher proportion of age-increased genes with increasing t, and an increase in the size of the green region denotes a higher proportion of age-decreased genes with increasing t. For any given threshold t, blue symbols denote gene sets for which the average 40-year fold-change among genes with at least t binding sites is significantly different from that of genes with fewer than t binding sites (P<0.05; two-sample t-test). The dotted horizontal line represents the average 40-year fold-change (old/young) among all 18,442 human genes included in the analysis. (TIF) [file pone.0033204.s005.tif]

# Intergenic Non-Coding Sequences

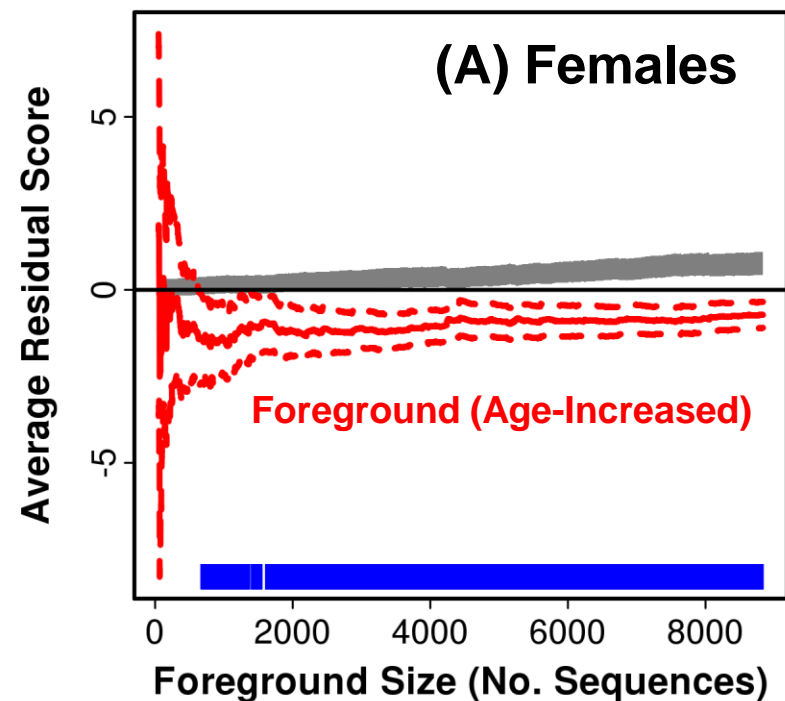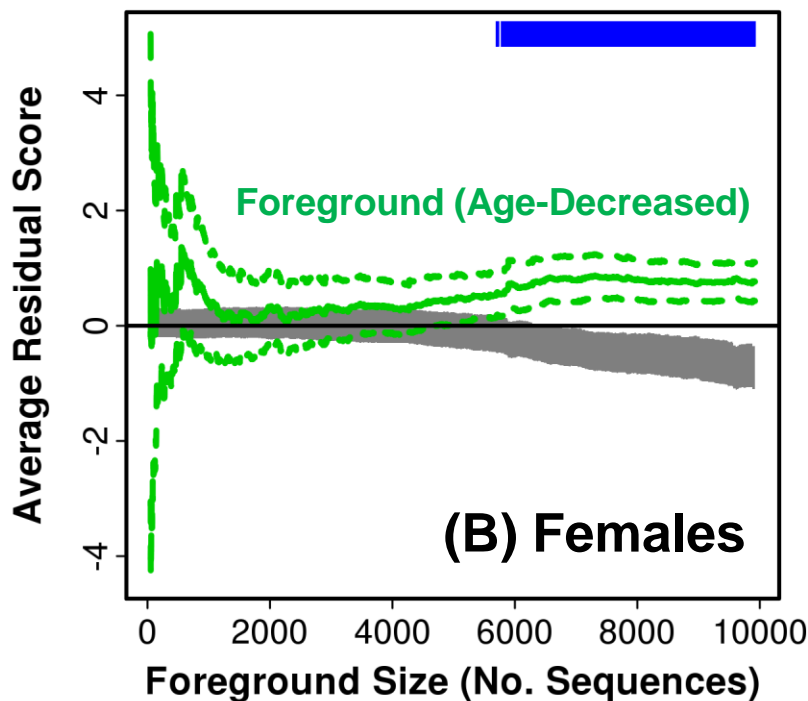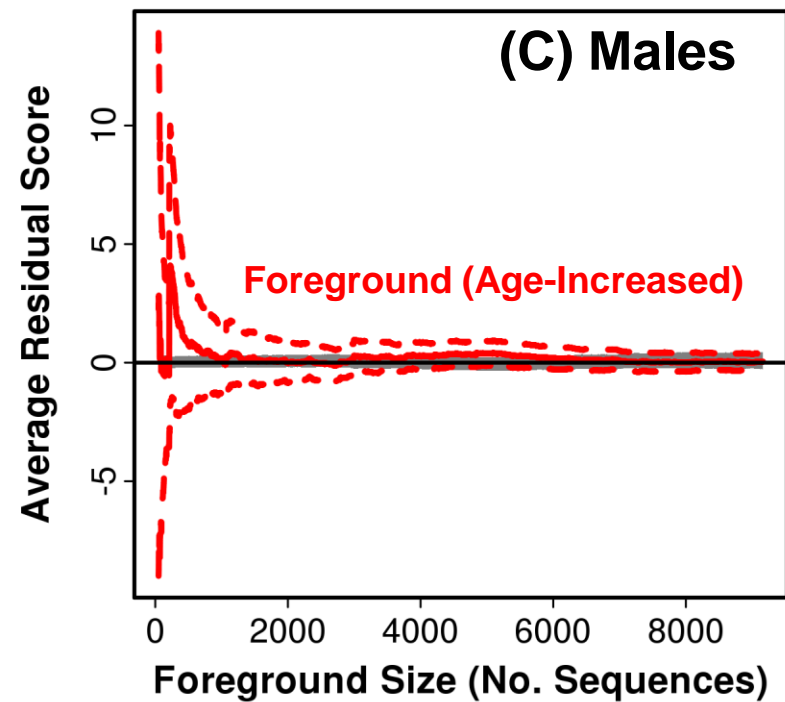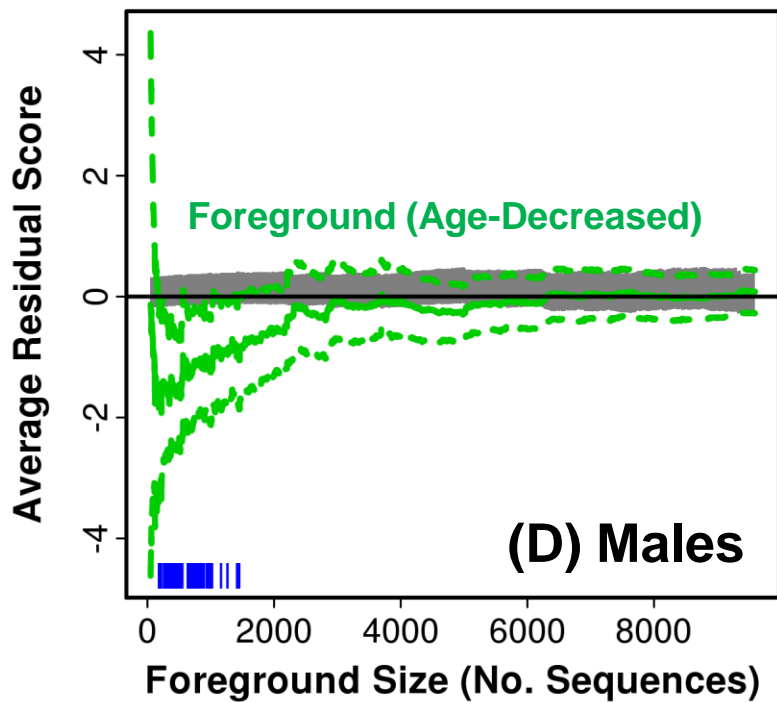

# Intronic Sequences

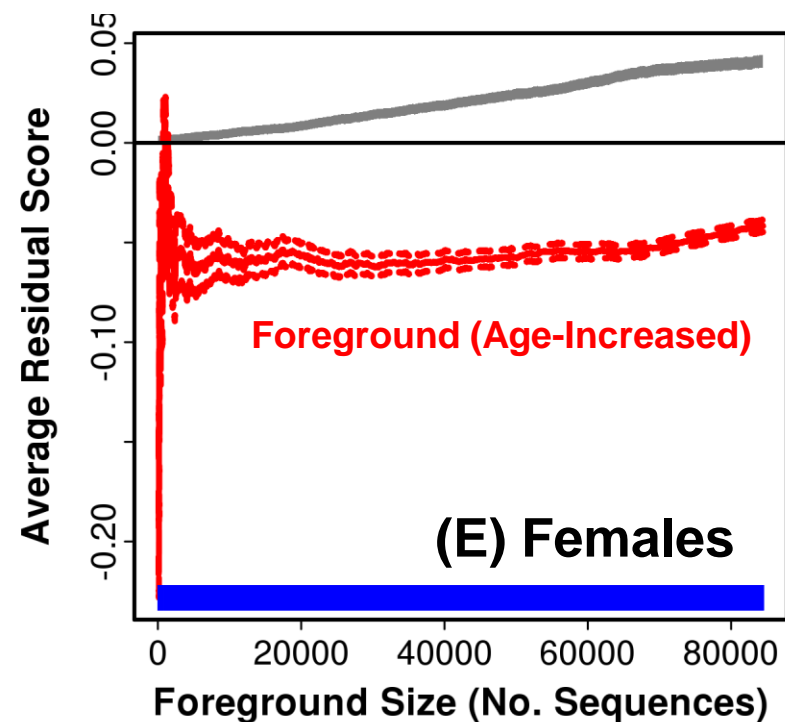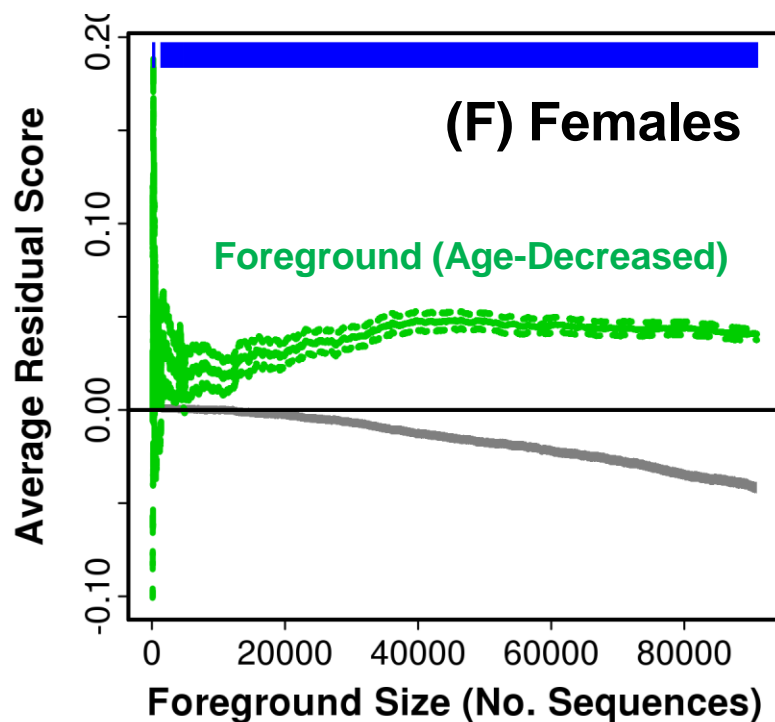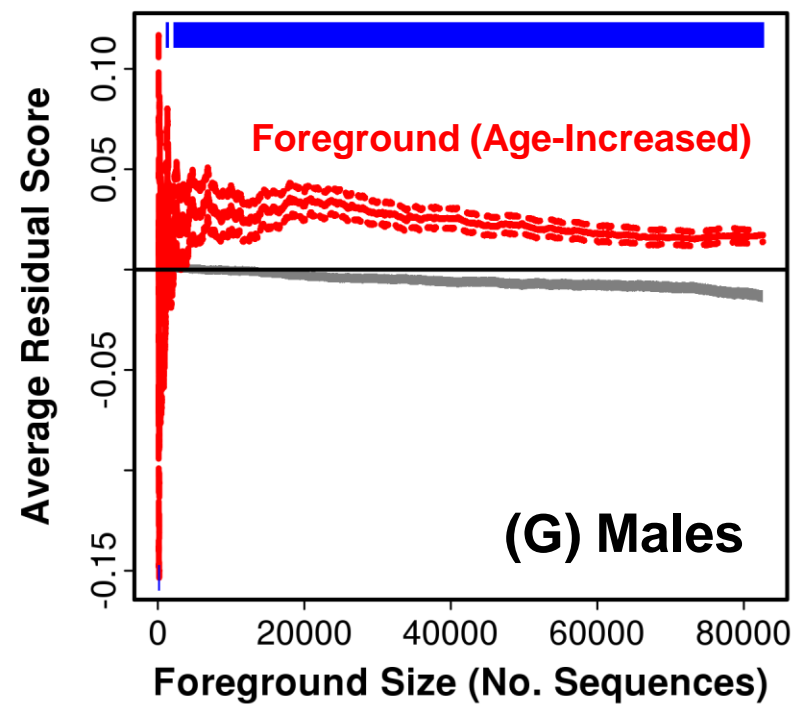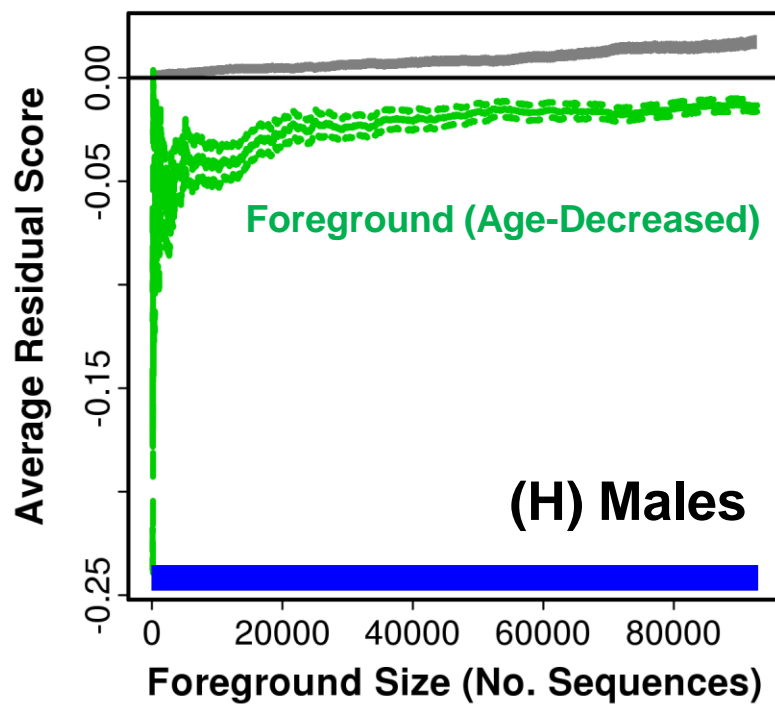

# Conserved Sequences (2000 BP Upstream TSS)

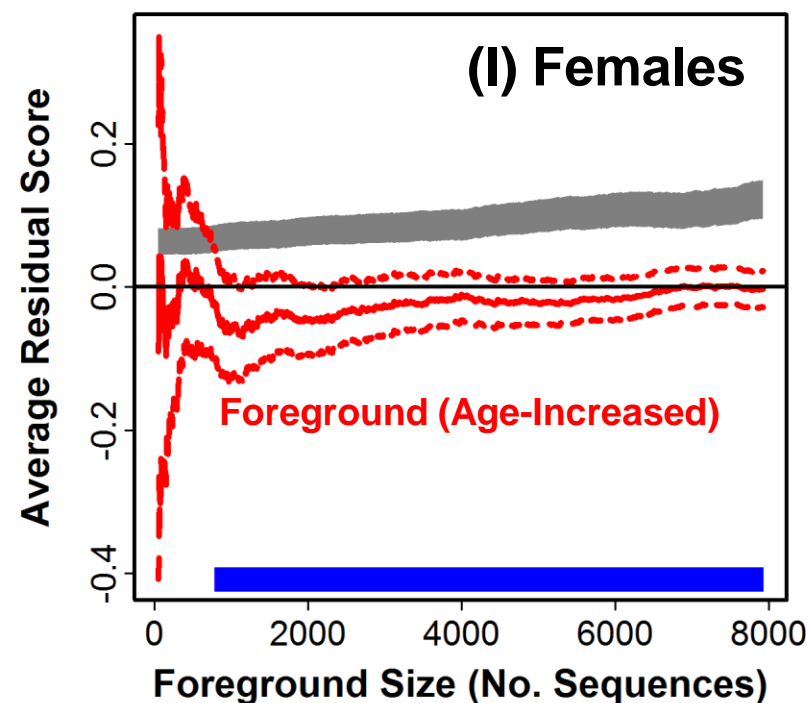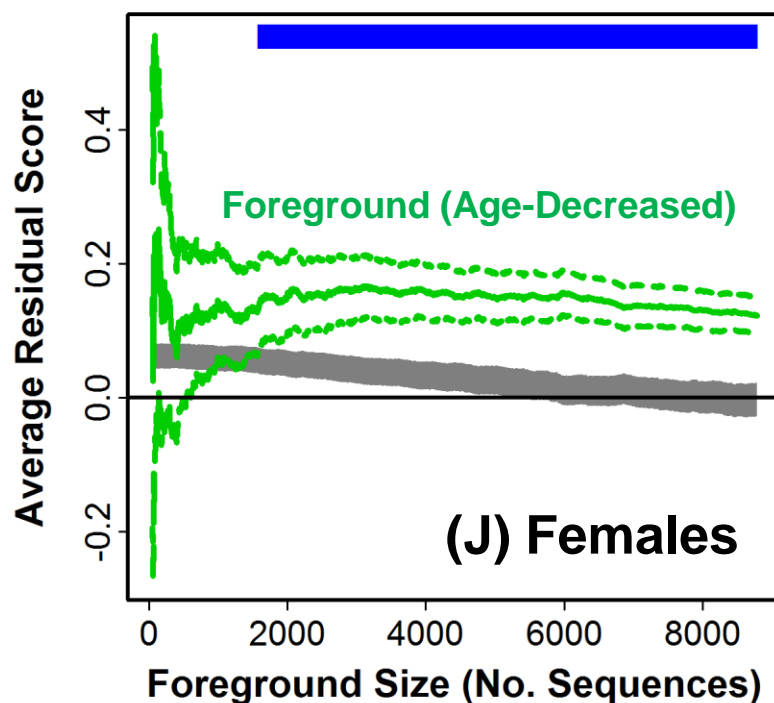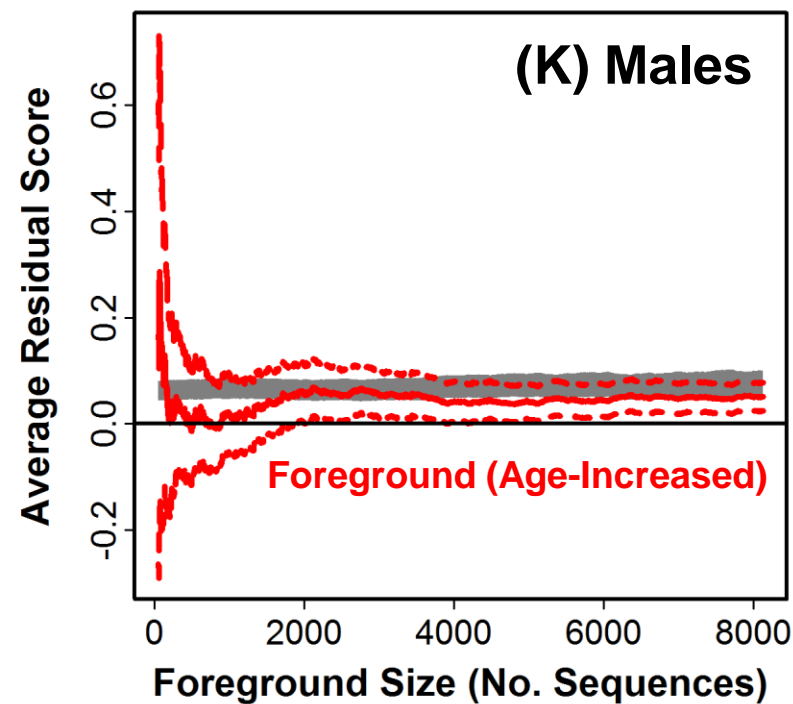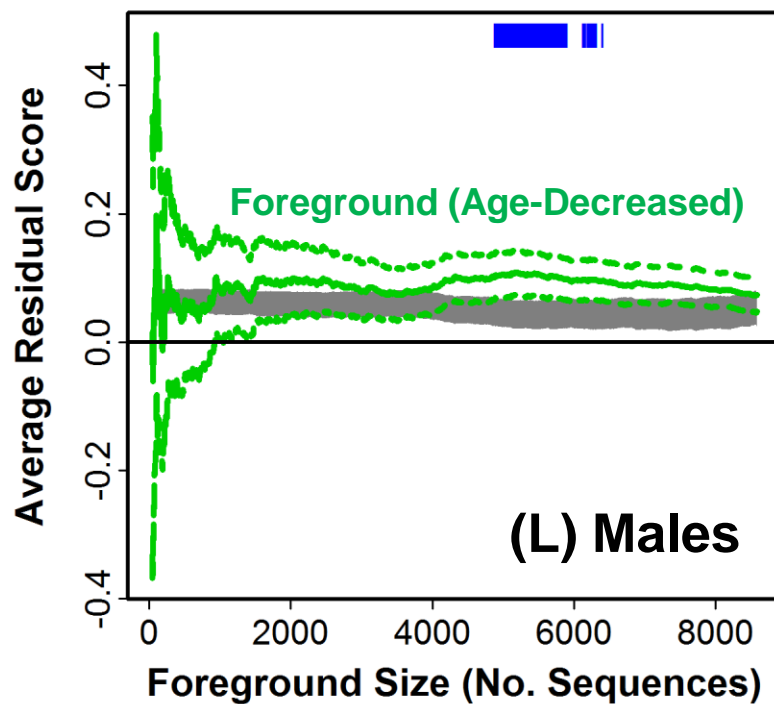

Supplement: Figure S6 — Genomic regions with increased AP-2 binding site density are associated with decreased gene expression with age in female human subjects but not male subjects. These analyses were performed with respect to all human non-coding intergenic regions (repeat-masked) (parts A–D), all intronic regions (repeat-masked) (parts E–H), and conserved sequences located 2000 BP upstream of the annotated TSS for all human genes (parts I–L). In each case, sequences were scanned to identify putative AP-2 binding sites (consensus motif: GCC----G), and length-adjusted residual scores were calculated for all sequences (see Methods). Positive scores indicate a large number of AP-2 binding sites (given sequence length) and negative scores indicate a small number of AP-2 binding sets (given sequence length). Average residual scores were then compared between a foreground set of sequences (adjacent to age-increased or age-decreased genes) and a background set of sequences (i.e., all other sequences excluding those in the foreground). The central tendency of the foreground sequence residual scores is displayed for (A, E, I) sequences within or adjacent to age-increased genes (red lines) in females, (B, F, J) sequences within or adjacent to age-decreased genes in females (green lines), (C, G, K) sequences within or adjacent to age-increased genes (red lines) in males, and (D, H, L) sequences within or adjacent to age-decreased genes in males (green lines). In each case, red or green lines represent the average residual score among foreground sequences (middle line) along with 95% confidence interval bounds (lower and upper lines). The grey region outlines a 95% confidence interval for the residual scores among sequences in the background set. Significant differences between the average foreground and background residual score are noted by blue bars near the top or bottom of each figure. Analyses were repeated using foreground sequence sets of varying size and selectivity (see horizontal axis). [file pone.0033204.s006.pdf]

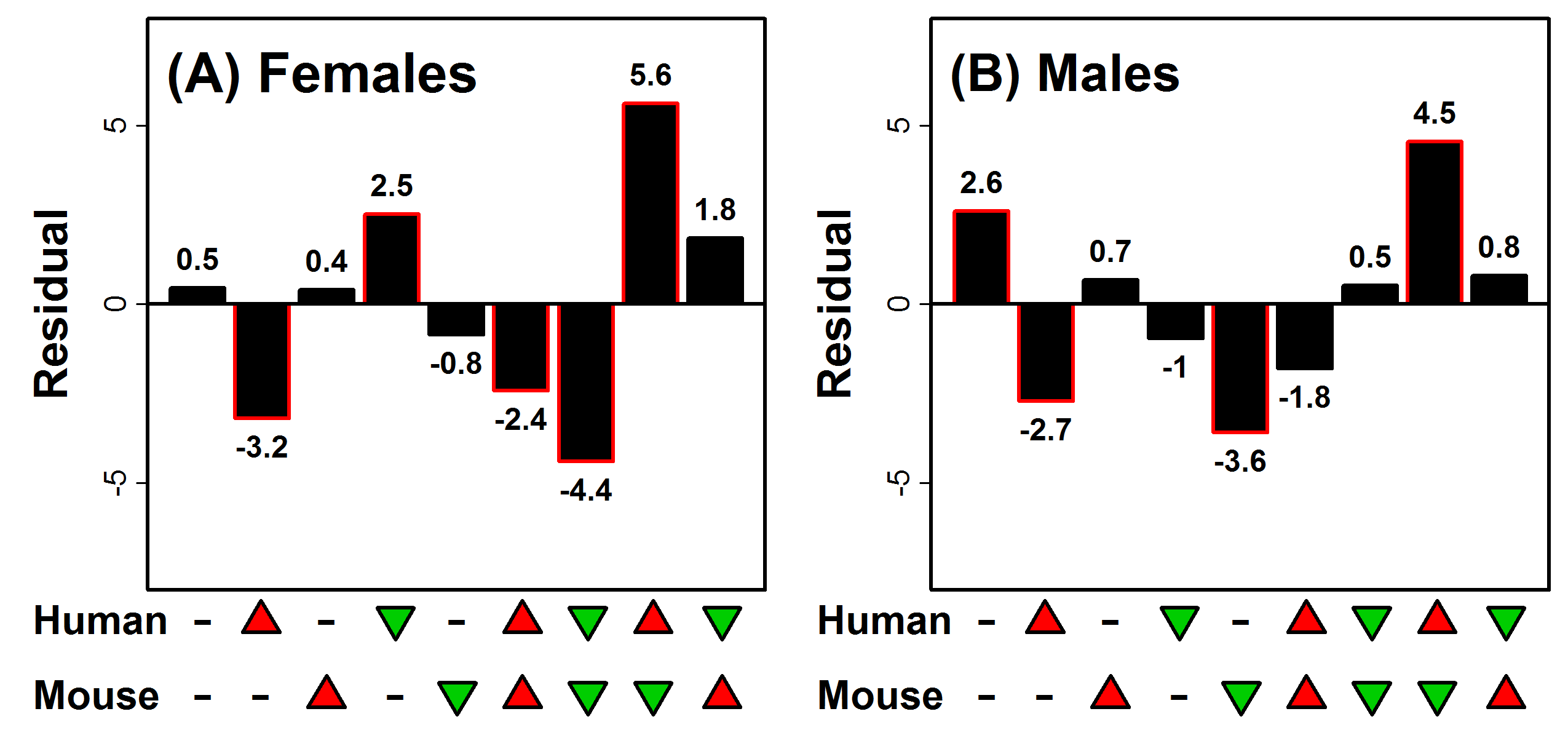

Supplement: Figure S7 — Weak association between aging effects in sun-protected human skin and tail skin from CB6F1 mice (adjusted residuals). A set of 15,203 human-mouse orthologous transcript pairs were placed into one of nine categories (horizontal axis in each panel) depending on whether transcripts were increased by aging (P<0.05; red up-triangles), decreased by aging (P<0.05; green down-triangles) or not altered by aging (black dash) in human sun-protected skin and/or tail skin from CB6F1 mice. For each category, an adjusted residual was calculated (vertical axis), which reflects departure from random association between human and mouse differential expression signatures (see Swindell [11]). Positive residuals indicate an overabundance of human-mouse transcript pairs within a particular category, while negative residuals indicate underabundance of human-mouse transcript pairs within a category. Under the null hypothesis (random association between human and mouse differential expression results), adjusted residuals are expected to follow a standard normal distribution, with residuals larger than three in absolute value providing evidence for a significant association between species. Correspondence between human-mouse aging patterns would have been supported by positive residuals for transcript pairs increased or decreased in both species (i.e., the fourth and third categories from the right), or alternatively, by negative residuals for transcript pairs with conflicting aging effects in the two species (i.e., the last two categories on the right). (TIF) [file pone.0033204.s007.tif]

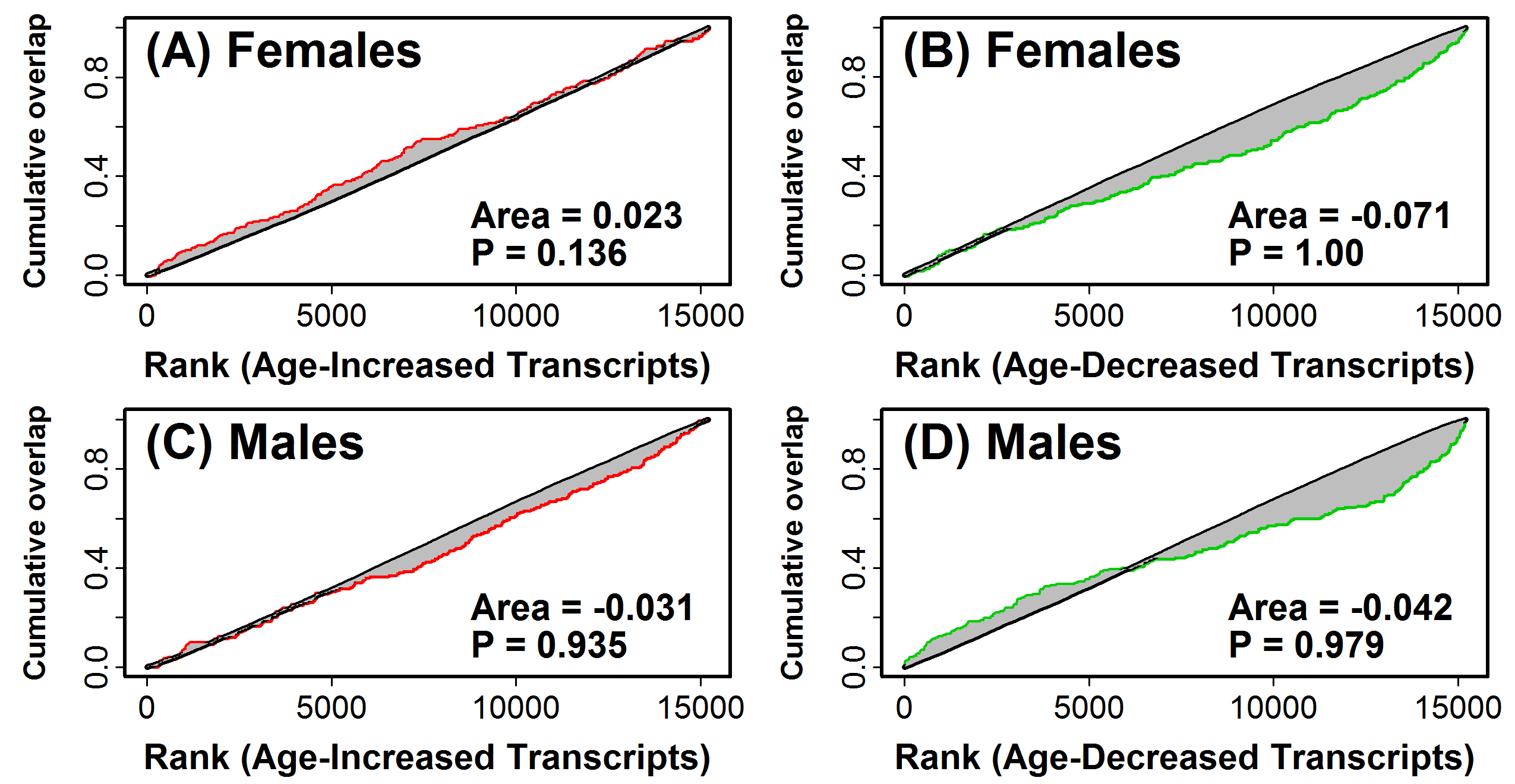

Supplement: Figure S8 — Weak association between aging effects in sun-protected human skin and tail skin from CB6F1 mice (AUC statistics). 15,203 mouse transcripts were ranked according to the strength of aging effects among orthologous human genes in sun-protected skin (horizontal axis). This ranking was based upon (A) the degree of increase with aging in human females, (B) the degree of decrease with aging in human females, (C) the degree of increase with aging in human males or (D) the degree of decrease with aging in human males. In each panel (A)–(D), mouse transcripts associated with the strongest aging effect in humans (with respect to the human orthologue) were assigned lower ranks. Foreground sets of 200 mouse transcripts were then identified as those most strongly (A) increased with aging in mouse females, (B) decreased with aging in mouse females, (C) increased with aging in mouse males and (D) decreased with aging in mouse males. Red or green curves track the cumulative overlap (vertical axis) between the 200 foreground transcripts and the ranked set of 15,203 mouse transcripts. Enrichment of foreground genes with respect to the ranked lists (horizontal axis) is supported by red or green curves above the diagonal line, with a large positive area between the colored line and the diagonal (i.e., the region shaded in grey). This area is proportional to the Wilcoxon-Mann-Whitney (WMW) statistic, and for a large set of foreground genes chosen at random, is expected to follow a standard normal distribution (see equations 5–7 from Philippakis et al. [35]). The standard normal was therefore used as a null distribution to evaluate the significance of WMW statistics obtained in (A)–(D) and to generate the p-value listed in the lower-right of each panel. (TIF) [file pone.0033204.s008.tif]

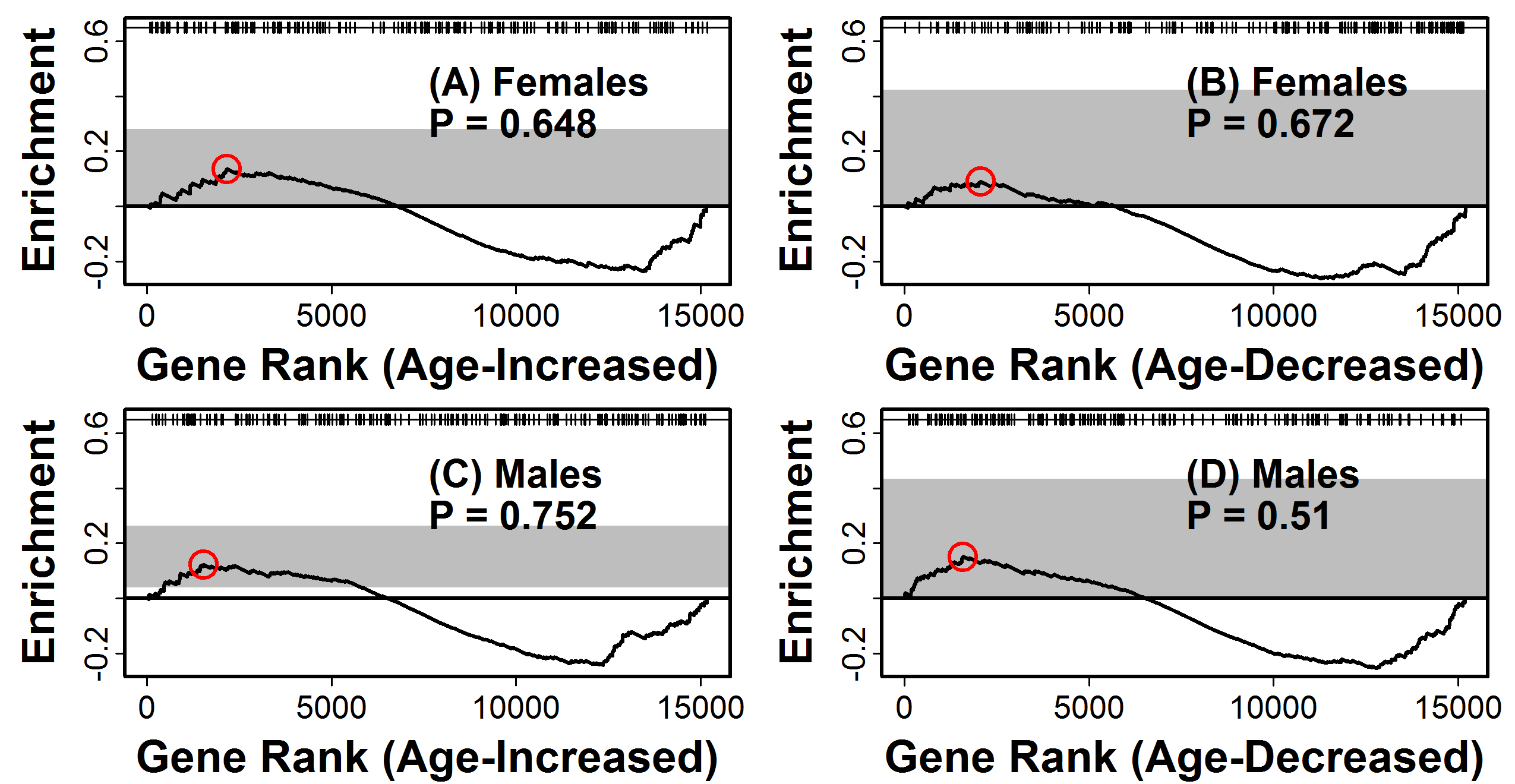

Supplement: Figure S9 — Weak association between aging effects in sun-protected human skin and tail skin from CB6F1 mice (Gene Set Enrichment Analysis). 15,203 mouse transcripts were ranked according to the strength of aging effects among orthologous human genes in sun-protected skin (horizontal axis). This ranking was based upon (A) the degree of increase with aging in human females, (B) the degree of decrease with aging in human females, (C) the degree of increase with aging in human males or (D) the degree of decrease with aging in human males. In each panel (A)–(D), mouse transcripts associated with the strongest aging effect in humans (with respect to the human orthologue) were assigned lower ranks. Foreground sets of 200 mouse transcripts were then identified as those most strongly (A) increased with aging in mouse females, (B) decreased with aging in mouse females, (C) increased with aging in mouse males and (D) decreased with aging in mouse males. The enrichment (vertical axis) is a measure of the cumulative overlap between the foreground set of mouse transcripts and the ranked list of 15,203 mouse transcripts (see Subramanian et al. [36]). The enrichment score (ES) metric is the maximum enrichment observed across all ranked genes (see red circle) and is used as the test statistic. The null distribution of ES was generated from 1000 simulation trials in which the 15,203 mouse transcripts were ranked based upon randomized expression data. The shaded grey region within each panel represents the lower 95% of the simulated null distribution for ES. Correspondence between human-mouse aging patterns is not supported in (A)–(D) because the red circle (ES) shown in each panel lies within the grey region (i.e., within the lower 95% of the ES null distribution). (TIF) [file pone.0033204.s009.tif]

**2-Year  
Fold Change  
(Old / Young)**

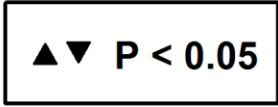



(C)

2-Year  
Fold Change  
(Old / Young)

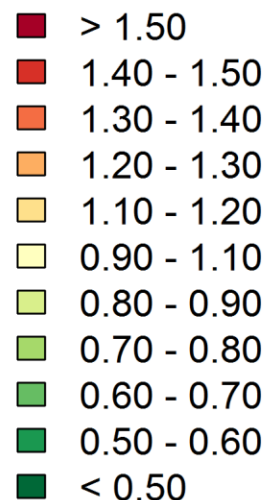

▲ ▼ P < 0.05

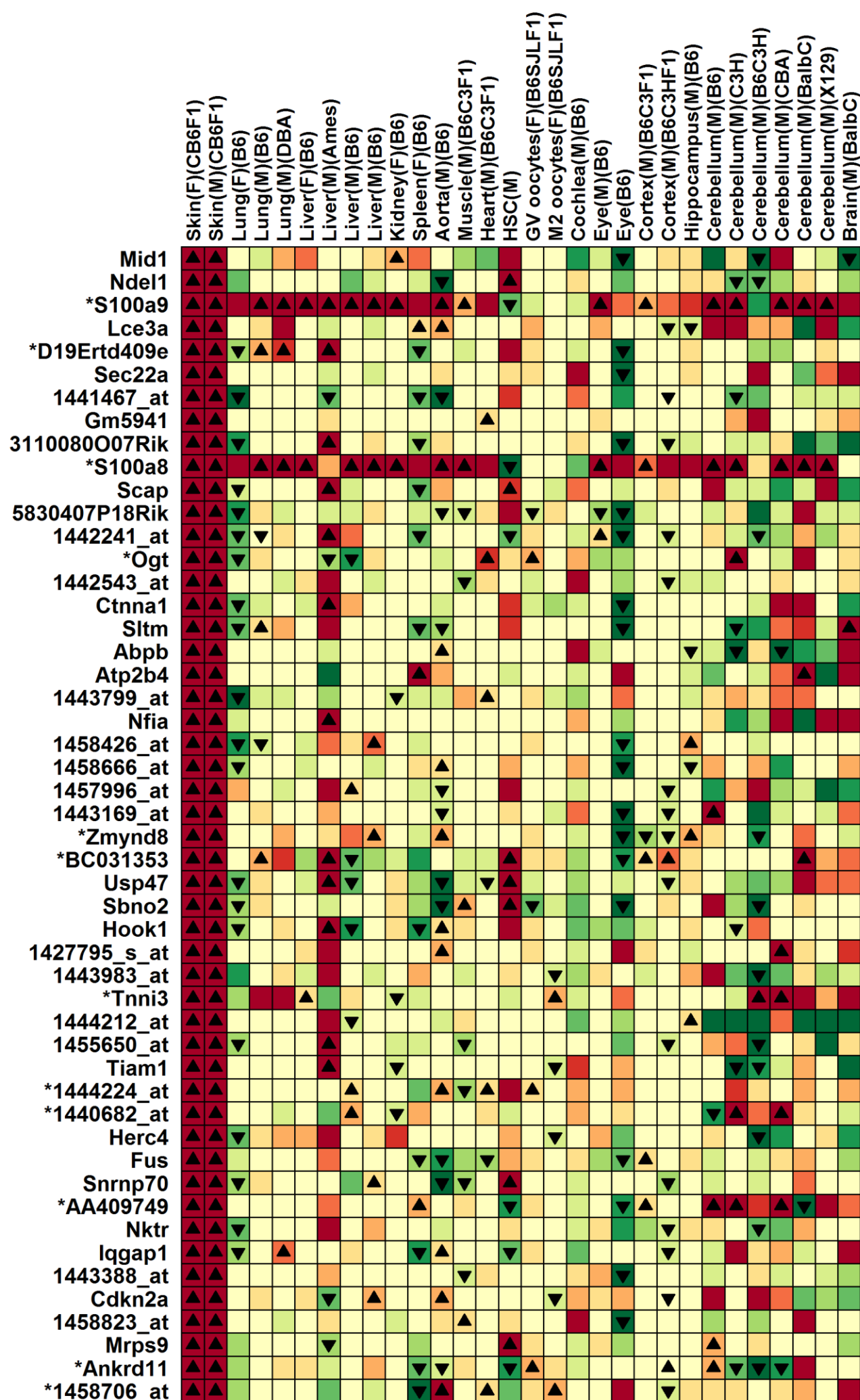

(D)

2-Year  
Fold Change  
(Old / Young)

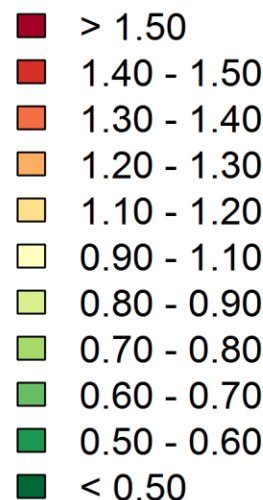

▲ ▼ P < 0.05

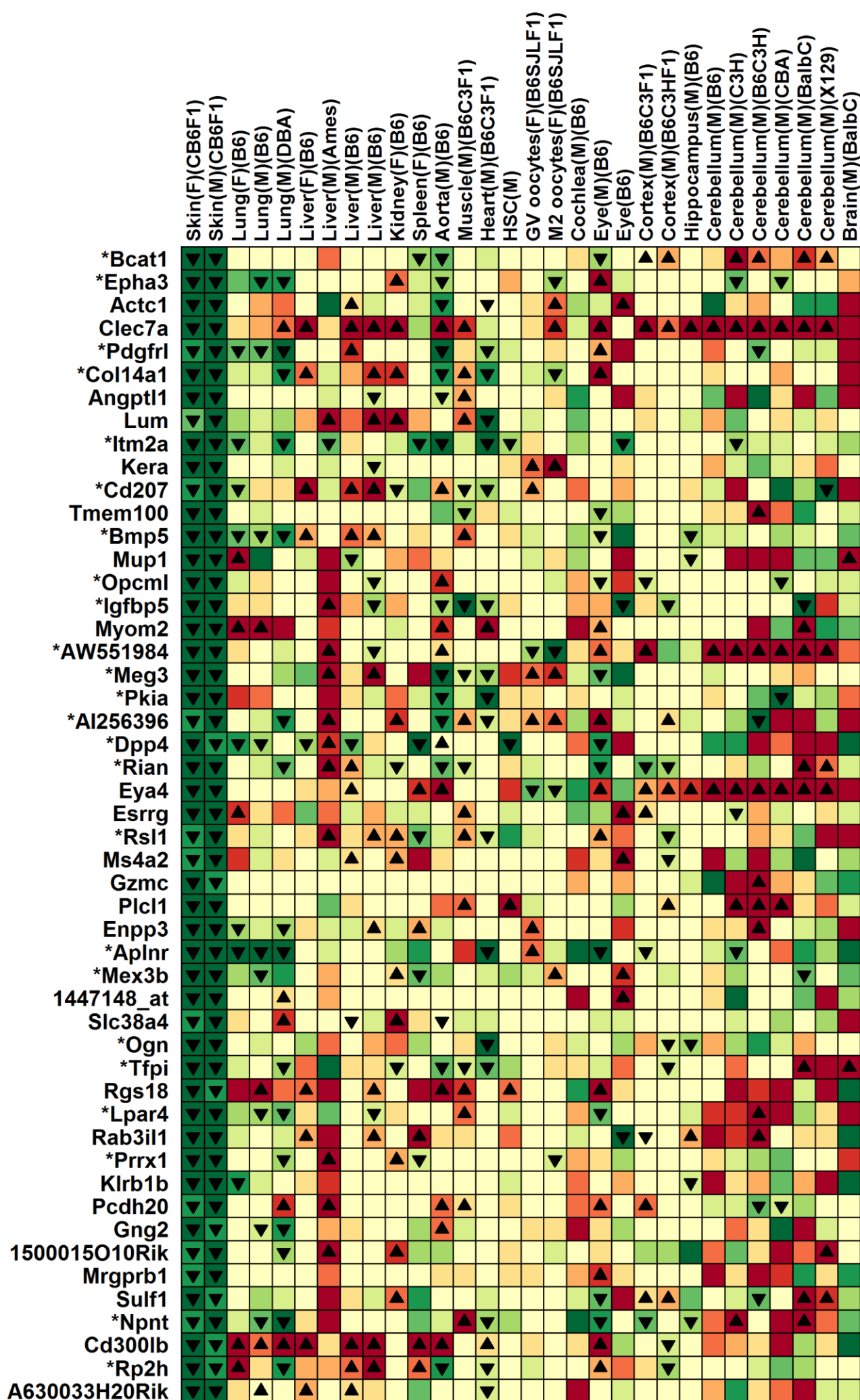

Supplement: Figure S10 — Ranked lists of genes altered by aging in mouse tail skin and other tissues. Tables display age-associated expression patterns for ranked lists of the top 50 genes (A) increased by aging across all mouse tissues, (B) decreased by aging across all mouse tissues, (C) increased by aging in mouse tail skin (CB6F1 strain), or (D) decreased by aging in mouse tail skin (CB6F1 strain). For a given gene (row) and tissue (column), colors denote the estimated fold-change expression ratio between an older mouse t+2 years of age and a younger mouse t years of age (a linear rate of change with age is assumed). Filled triangles denote significant aging effects (P<0.05). In (A) and (B), gene rankings are based upon the total number of significant results observed across all columns in each table, and an asterisk symbol is used to denote genes similarly altered between mouse skin (males and/or females) and the other aging profiles. In (C) and (D), genes were first filtered to include only those significantly altered by aging in skin (P<0.05), and then ranked according to fold-change estimates (old/young) (averaged between sexes). Genes for which expression is similarly altered in three or more other mouse aging profiles are indicated by an asterisk symbol. (PDF) [file pone.0033204.s010.pdf]

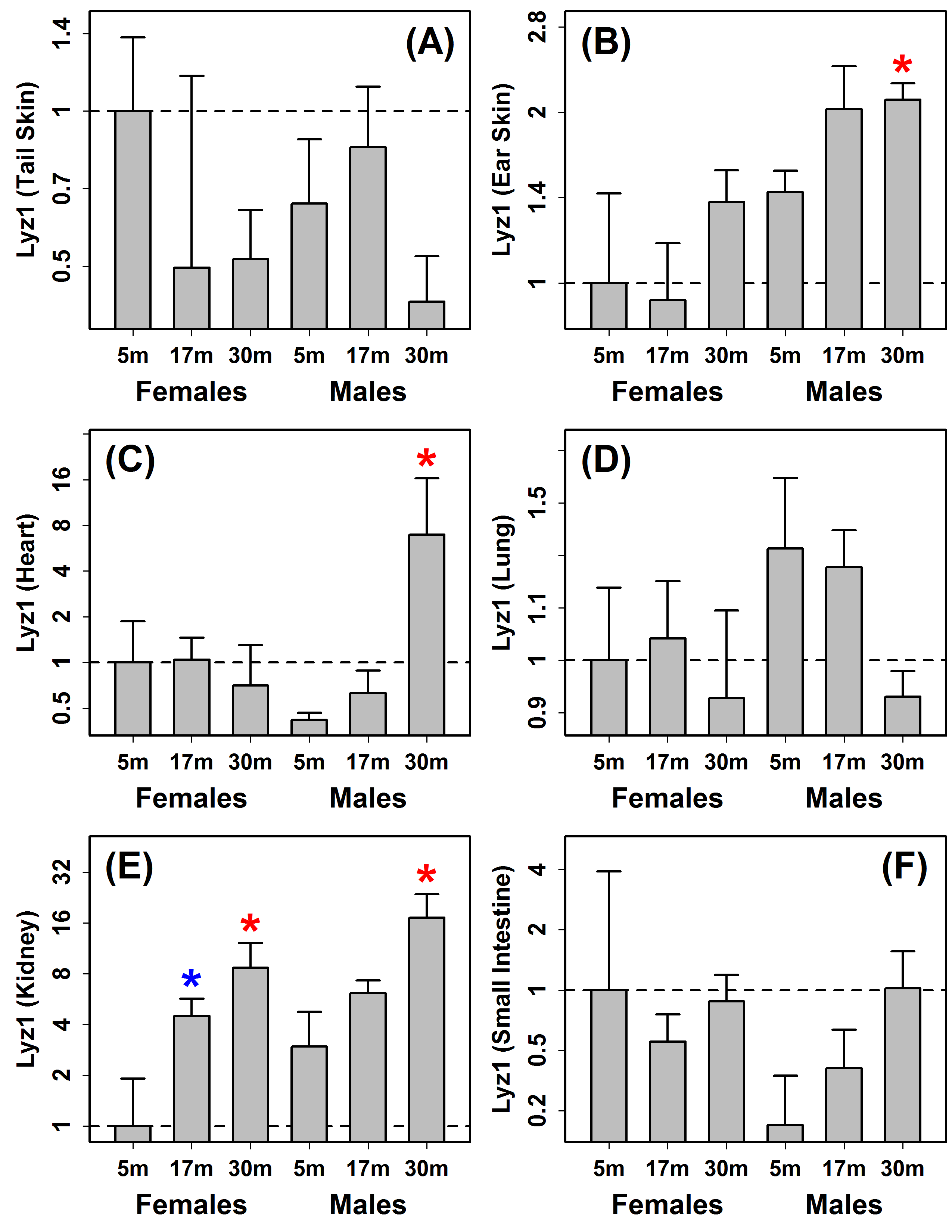

Supplement: Figure S12 — Lyz1 expression is not elevated with age in tail skin but increased by age in ear skin, heart and kidney from CB6F1 mice. RT-PCR was used to evaluate the expression of Lyz1 in mice from three age groups (5 months, 17 months and 30 months) and from both sexes (n = 5–6 mice for each sex/age combination). The expression of 18S ribosomal RNA (Rn18s) was used as an internal control gene for calculation of relative expression in each sample. Plots (A)–(F) show mean expression levels in each group (± one standard error) normalized to the average expression level of the young (5 month) female group. In each plot, and for each sex, the average expression of middle-aged (17 month) and old groups (30 months) was compared to that of the young group (5 months). A red star indicates a significant difference relative to the youngest group of the same sex (P<0.05; two-sided two-sample t-test). A blue star is used to indicate a significant difference based upon a two-sample non-parametric statistical test (P<0.05; either Wilcoxon or Kruskal-Wallis rank sum test). (TIF) [file pone.0033204.s012.tif]

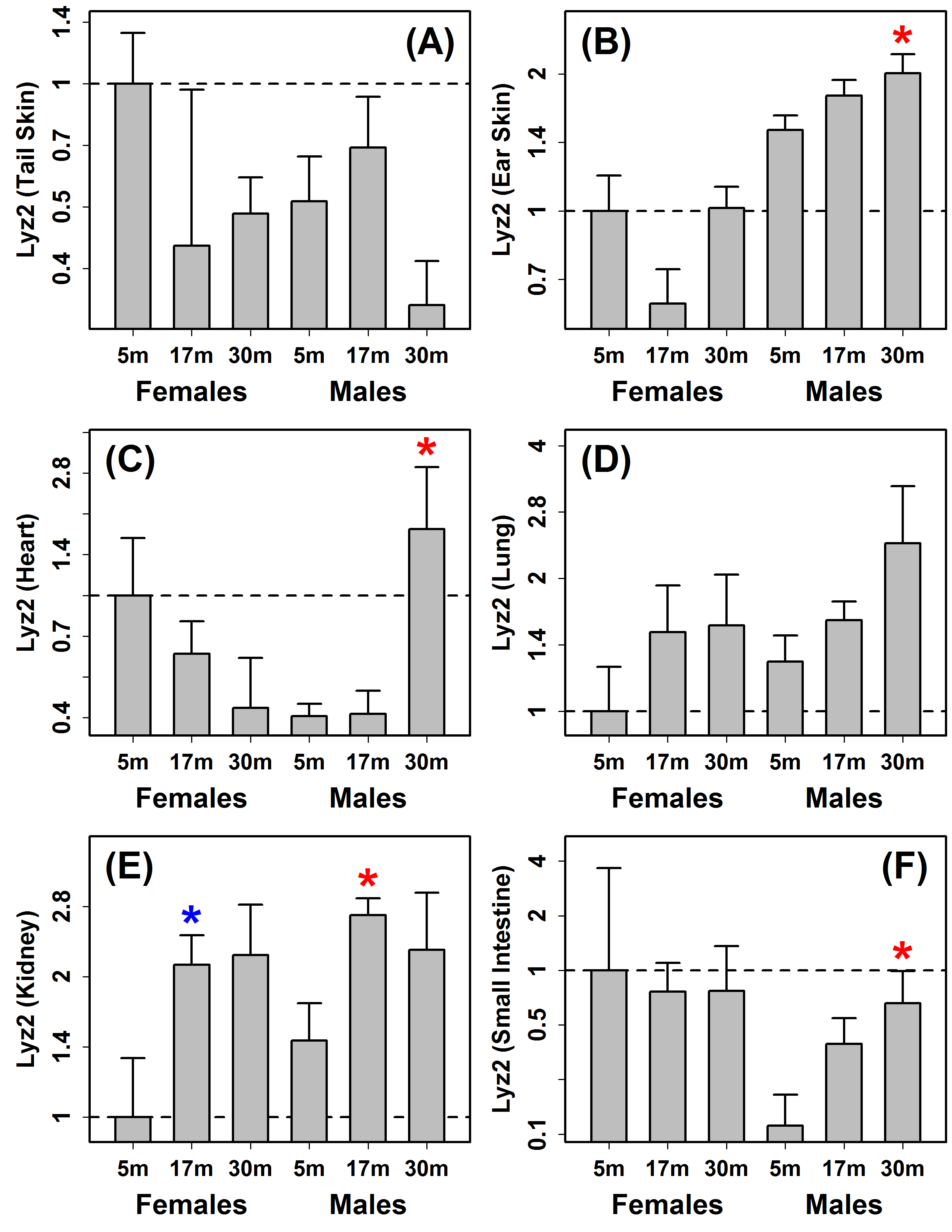

Supplement: Figure S13 — Lyz2 expression is not elevated with age in tail skin but increased by age in ear skin, heart, kidney and small intestine from CB6F1 mice. RT-PCR was used to evaluate the expression of Lyz2 in mice from three age groups (5 months, 17 months and 30 months) and from both sexes (n = 5–6 mice for each sex/age combination). The expression of 18S ribosomal RNA (Rn18s) was used as an internal control gene for calculation of relative expression in each sample. Plots (A)–(F) show mean expression levels in each group (± one standard error) normalized to the average expression level of the young (5 month) female group. In each plot, and for each sex, the average expression of middle-aged (17 month) and old groups (30 months) was compared to that of the young group (5 months). A red star indicates a significant difference relative to the youngest group of the same sex (P<0.05; two-sided two-sample t-test). A blue star is used to indicate a significant difference based upon a two-sample non-parametric statistical test (P<0.05; either Wilcoxon or Kruskal-Wallis rank sum test). (TIF) [file pone.0033204.s013.tif]

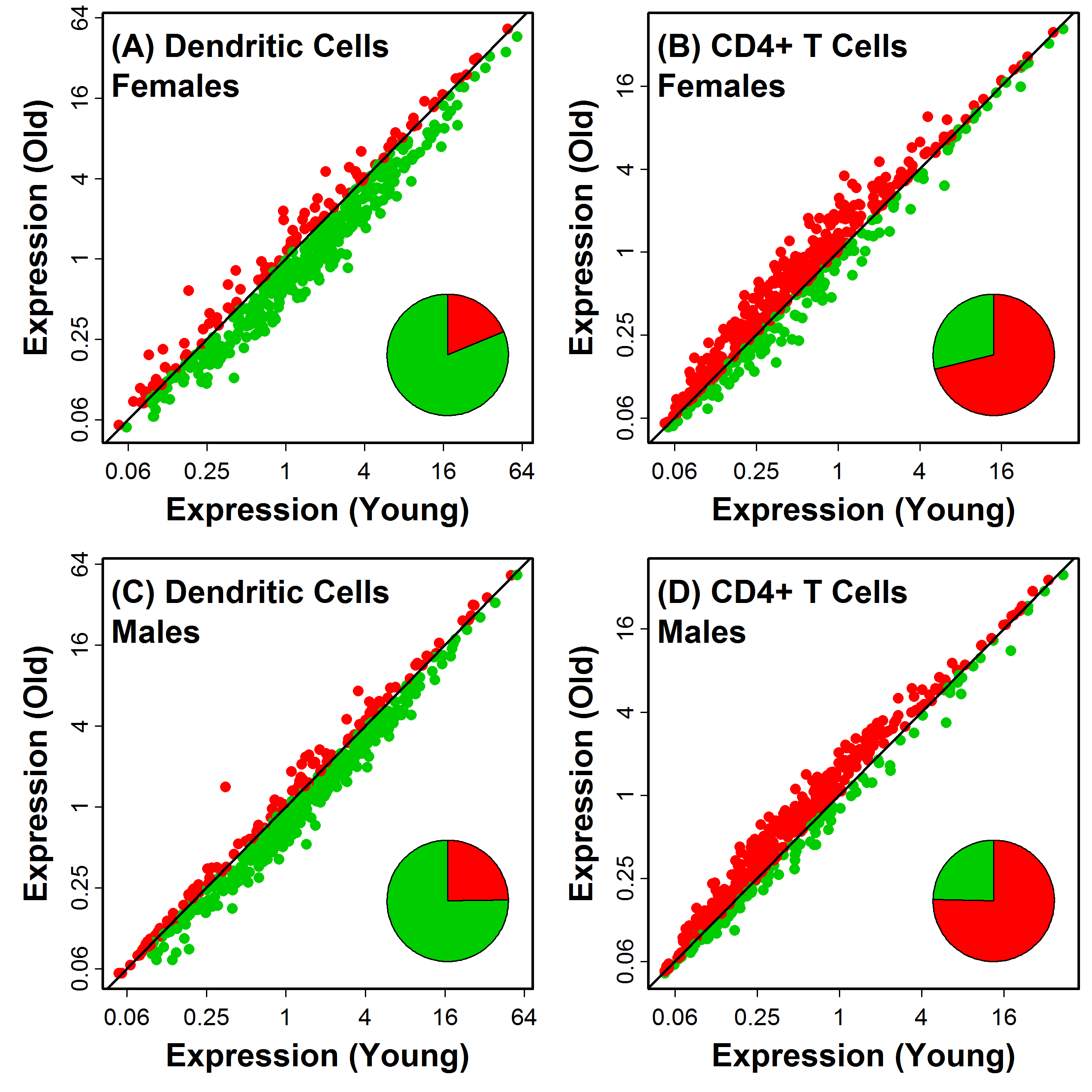

Supplement: Figure S14 — Aging of mouse tail skin leads to decreased expression of transcripts associated with dendritic cells but increased expression of CD4+ T cell-associated genes (CB6F1 strain). We identified 500 probe sets (from the Affymetrix Mouse Genome 430 2.0 Array) associated with signature transcripts that exhibit high expression in CD4+ T-cells isolated from spleen (based upon data provided under GEO series accession GSE20366). Likewise, we identified 500 probe sets associated with transcripts that exhibit high expression in dendritic cells harvested from lung tissue (GSE18607). The scatterplots (A)–(D) display expression of these transcripts in tail skin samples from young (5 month) and old (30 month) CB6F1 mice of both sexes (n = 5 per age group for each sex). In each scatterplot, red symbols represent probe sets with higher expression in old mice, while green symbols represent probe sets with higher expression in young mice. The relative proportion of these two probe set groups is indicated by the pie chart shown in each panel. (TIF) [file pone.0033204.s014.tif]

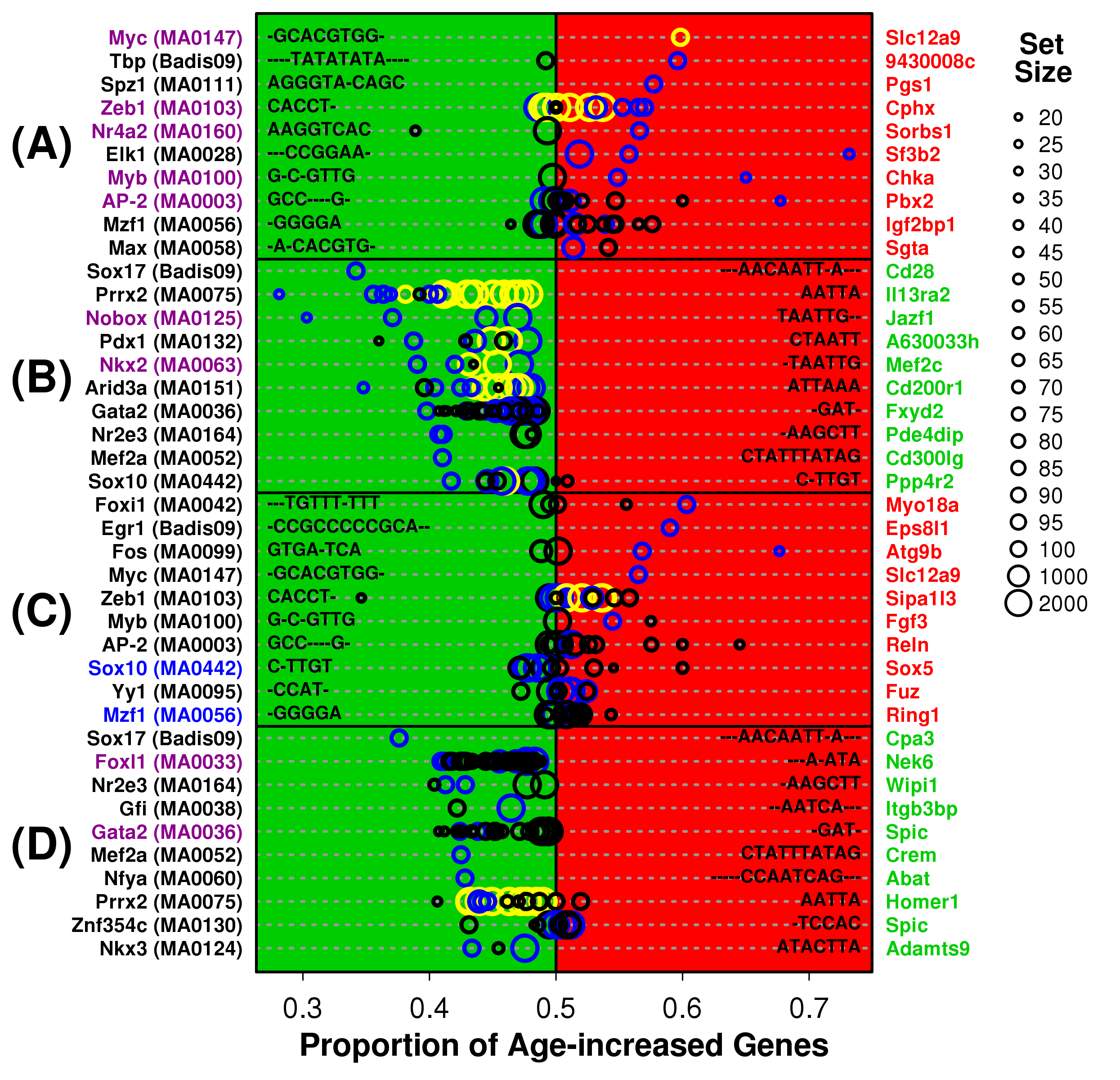

Supplement: Figure S15 — Top-ranked motifs associated with aging effects in mouse tail skin (CB6F1 strain): Overlap with top-ranked human motifs but dissimilar associations with aging. This figure is identical to Figure 8, except a simulation-based correction for multiple testing has been applied (yellow symbols). For each simulation trial, genes were randomly assigned to gene sets (625 sets in total), where the respective size of gene sets was matched to those evaluated in our analysis. Following this random assignment of genes to gene sets, p-values were generated from each set to test for age-biased expression (Fisher's Exact Test), and the lowest p-value arising among all gene sets was identified. This procedure was repeated in 2000 simulations, yielding a distribution for the minimum p-value expected to arise by chance alone, given the total number of gene sets evaluated for age-biased expression. We then identified the 0.05 quantile of this distribution (P*), which corresponds to the p-value that is lower than the minimal p-value that arose in 95% of the simulation trials. Those gene sets associated with p-values less than P* are denoted by yellow symbols. (TIF) [file pone.0033204.s015.tif]

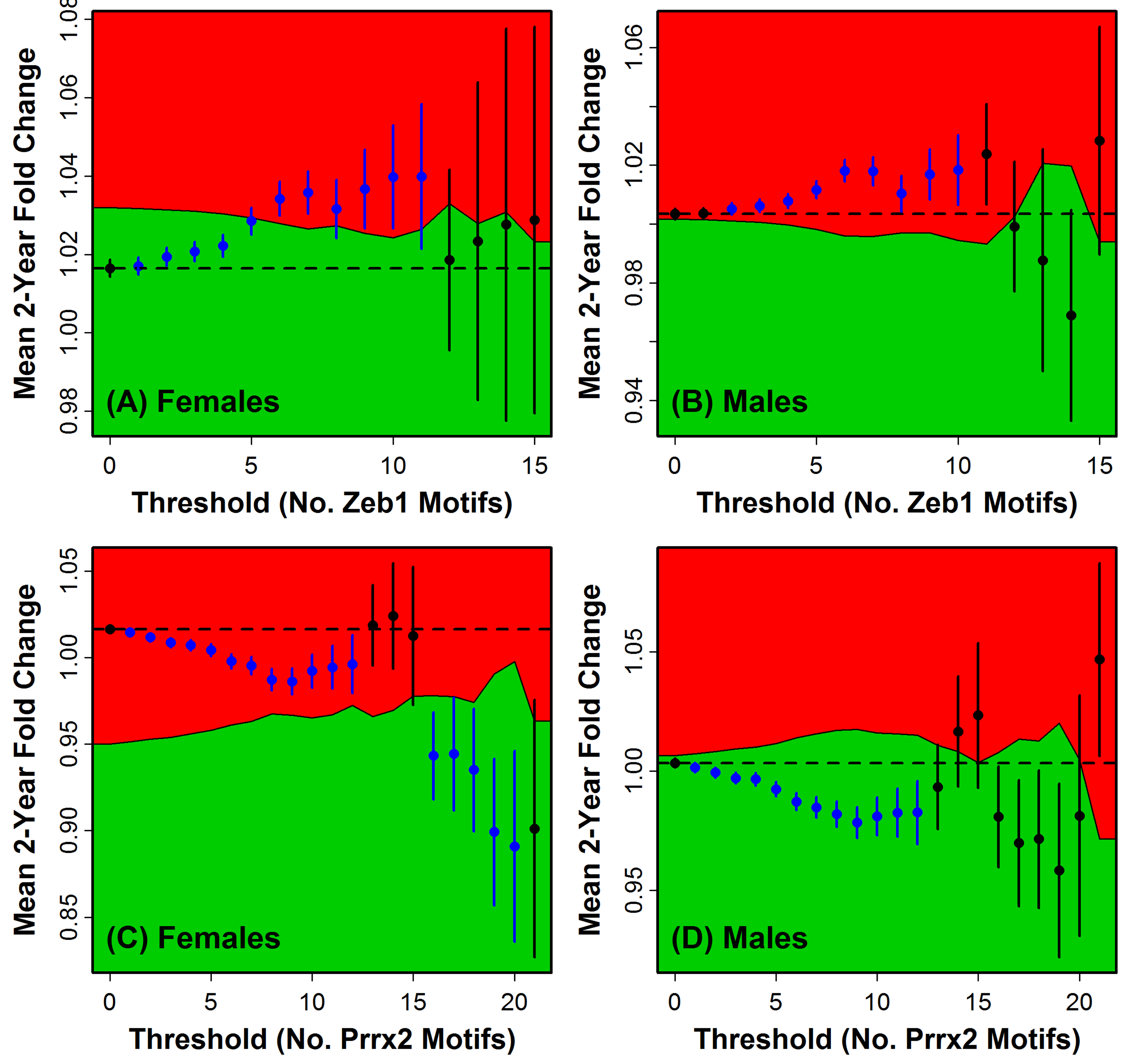

Supplement: Figure S16 — Zeb1 and Prrx2 motif density near the TSS is associated with age-related expression patterns in tail skin from both sexes (CB6F1 strain). In each figure, blue or black symbols represent the average 2-year fold-change (old/young) among genes with at least t binding sites in the region proximal to the annotated transcription start site (2 KB upstream and 200 BP downstream of the TSS). An increasing series of thresholds (t) was used to define gene sets of successively smaller sizes (horizontal axis), and for each set, the average 2-year fold-change was calculated (vertical axis). The background color (red or green) reflects the proportion of age-increased (red) to age-decreased genes (green) with respect to a given threshold (t), where an increase in the size of the red region denotes a higher proportion of age-increased genes, and an increase in the size of the green region denotes a higher proportion of age-decreased genes. For any given threshold t, blue symbols denote gene sets for which the average 2-year fold-change among genes with at least t binding sites is significantly different from that of genes with fewer than t binding sites (P<0.05; two-sample t-test). The dotted horizontal line represents the average 2-year fold-change (old/young) among all 17, 329 mouse genes included in the analysis. (TIF) [file pone.0033204.s016.tif]

# Intergenic Non-Coding Sequences

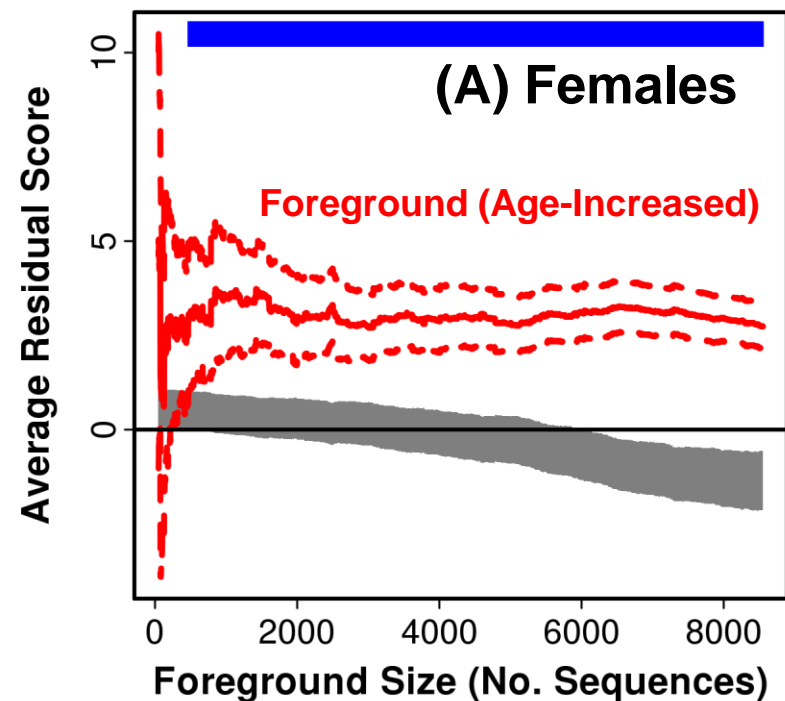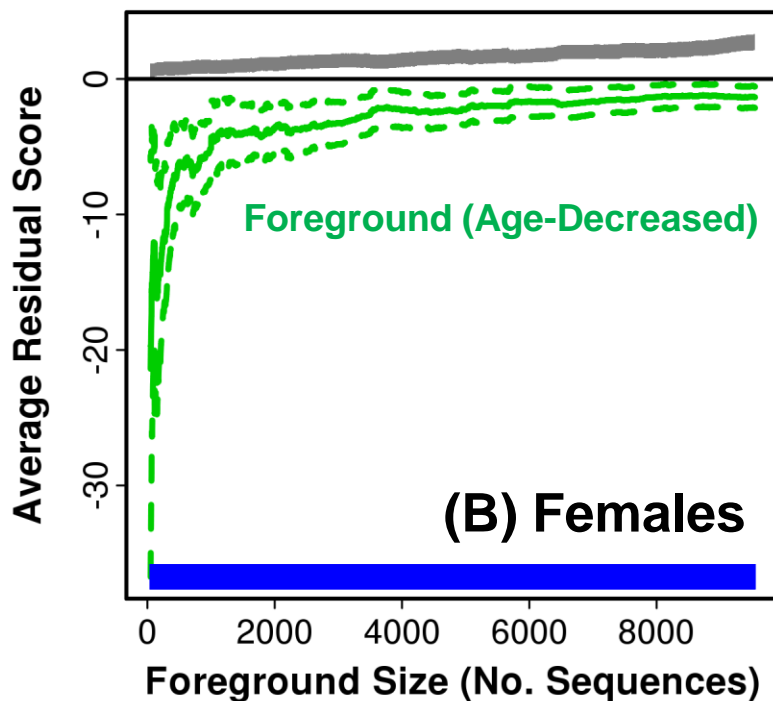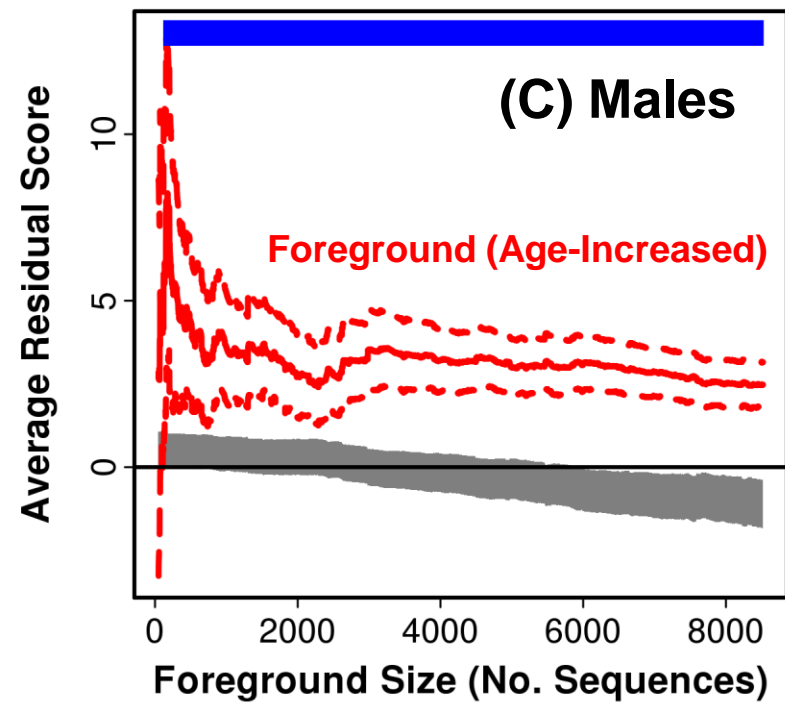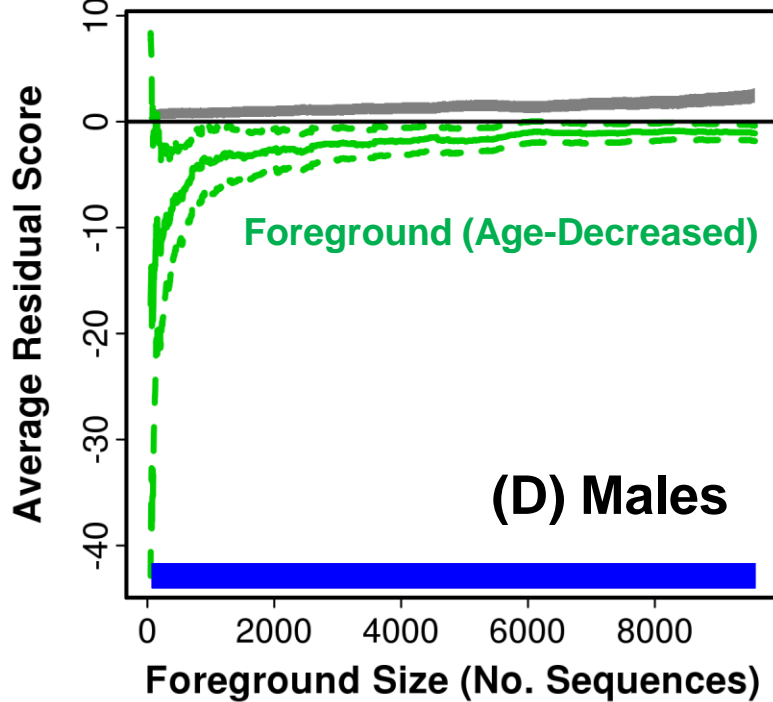

# Intronic Sequences

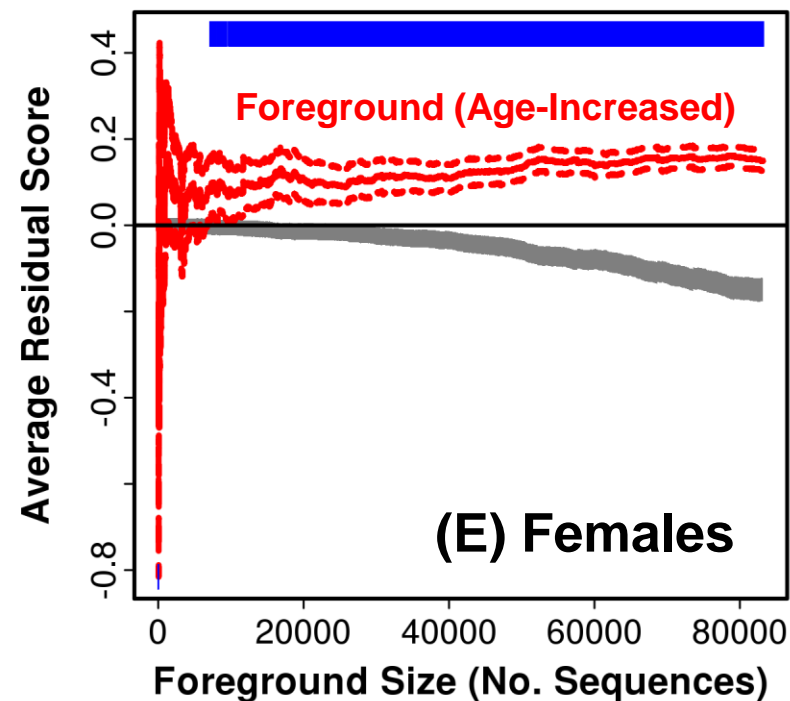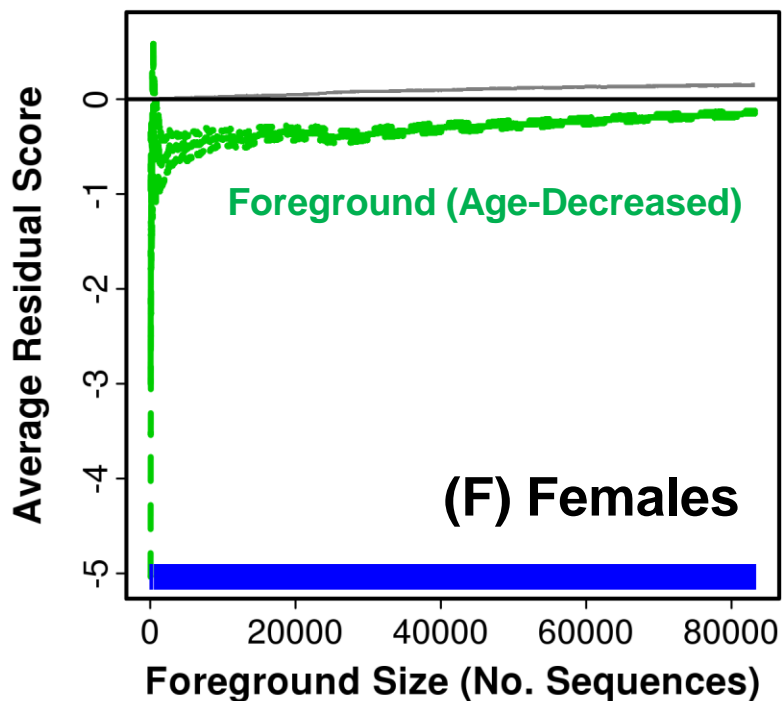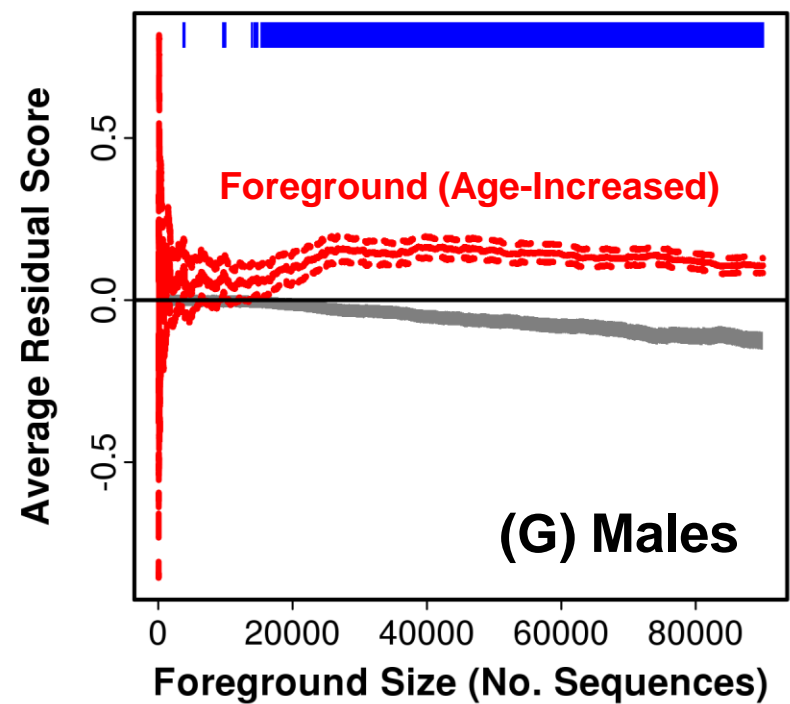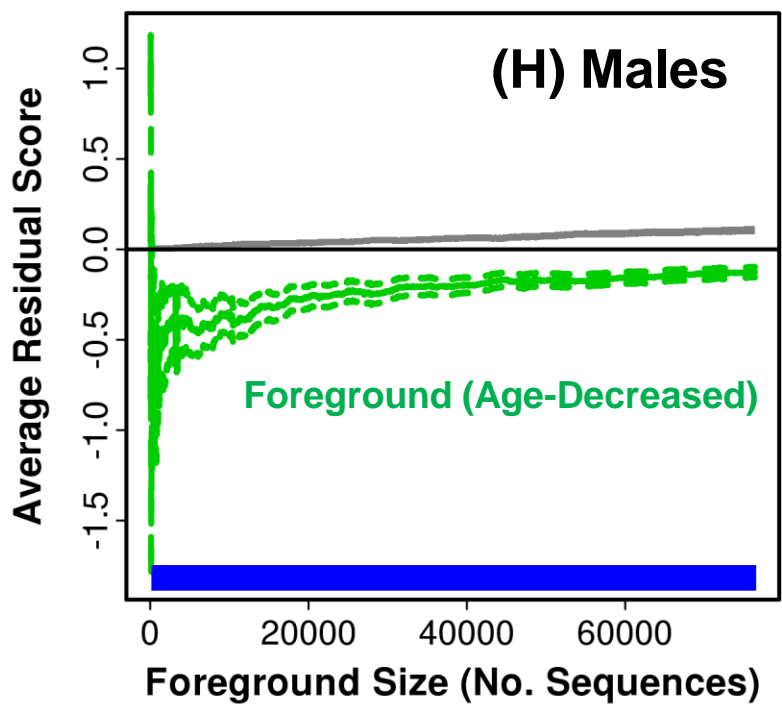

# Conserved Sequences (2000 BP Upstream TSS)

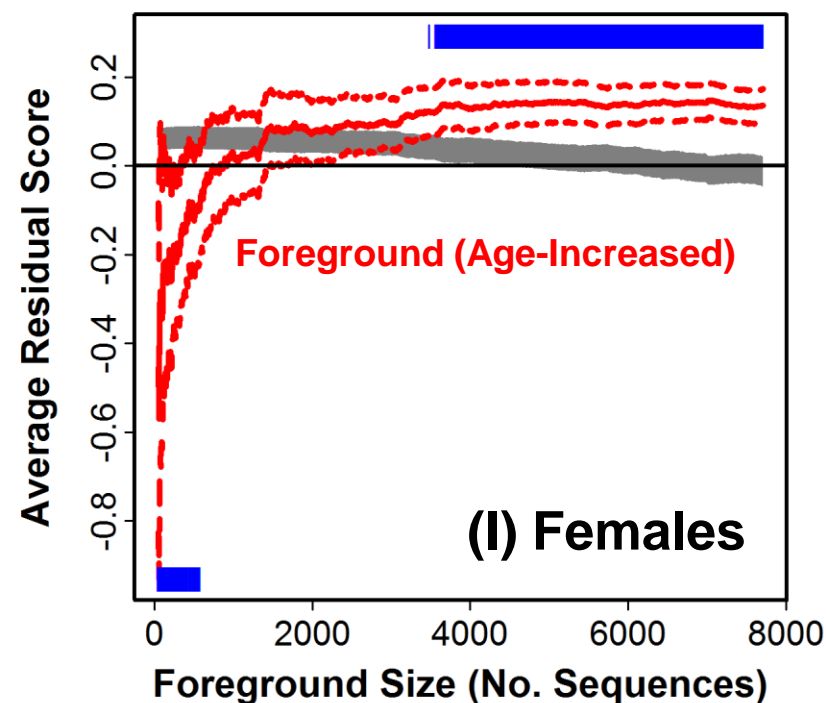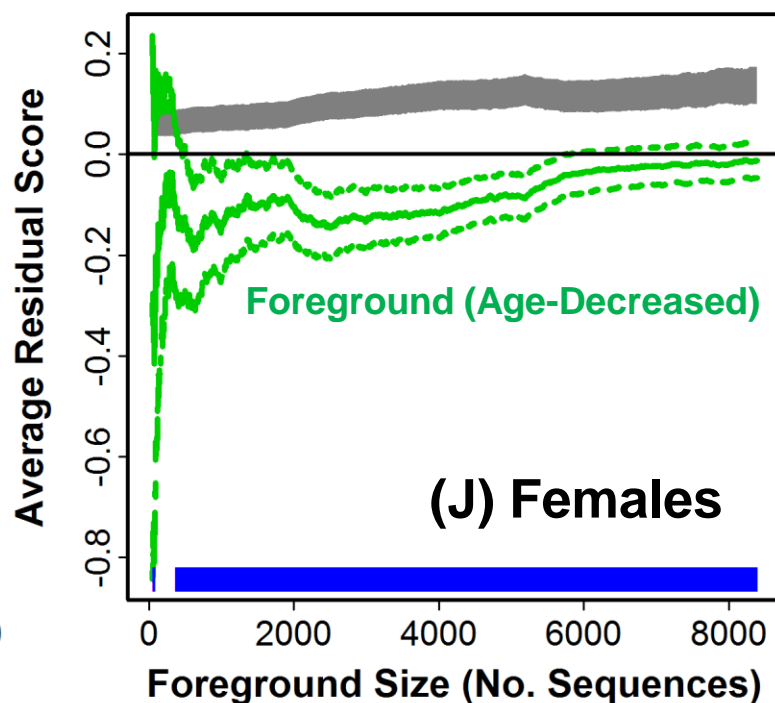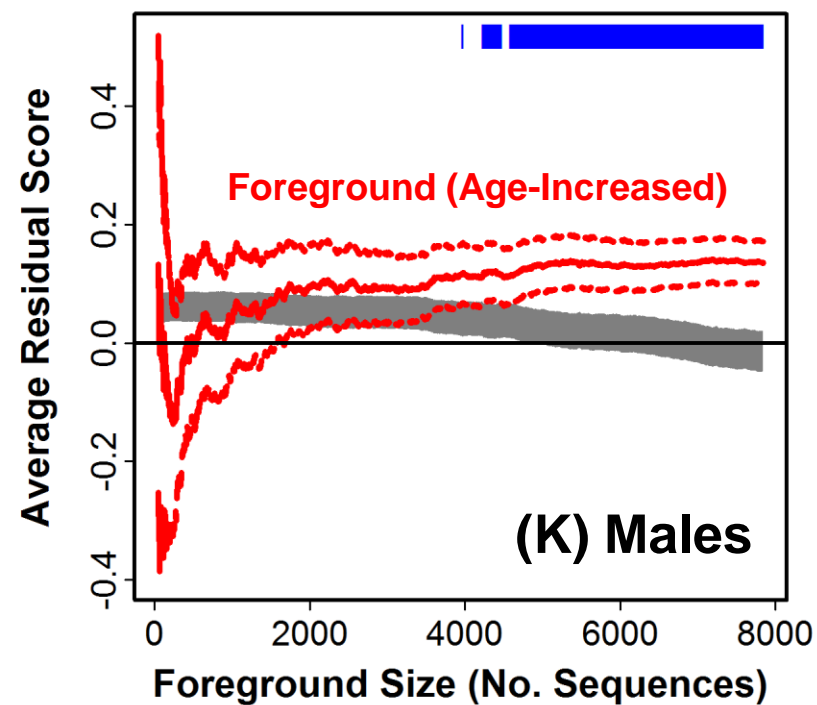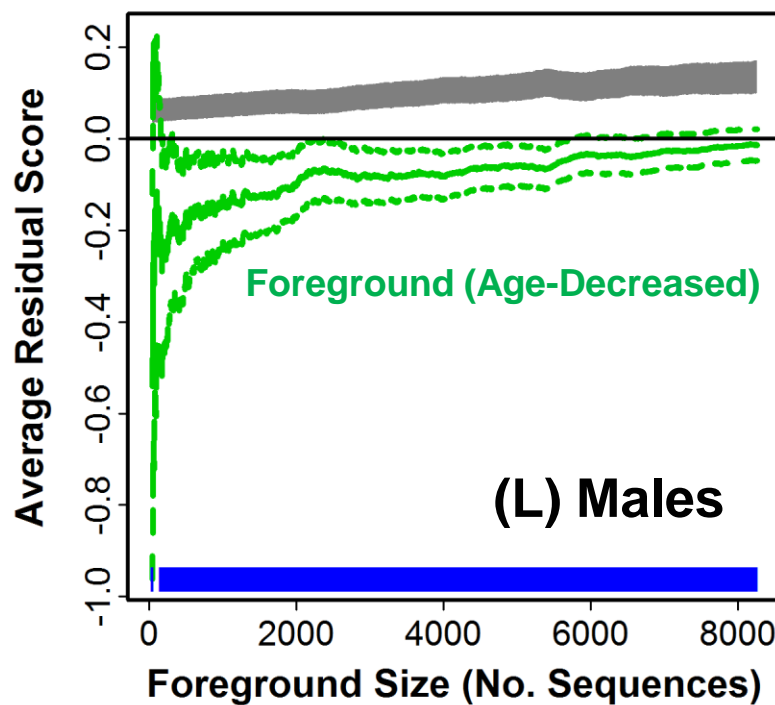

Supplement: Figure S17 — Genomic regions with increased Zeb1 motif density are associated with increased gene expression with aging of tail skin from both sexes (CB6F1 strain). The panels (A)–(L) compare Zeb1 motif density between sequences adjacent to or within genes exhibiting increased (or decreased) expression with age (i.e., foreground sequences), relative to sequences adjacent to or within genes that exhibit decreased (or increased) expression with age (i.e., background sequences) (i.e., as described in the AP-2 analysis shown in Figure S6). For each sequence examined, a length-adjusted residual score was calculated, which is proportional to the density of Zeb1binding sites within that sequence. Each panel shows the average residual score among sequences adjacent to or within age-increased genes (see red lines and 95% confidence intervals in A, C, E, G, I and K) or the average residual score among sequences adjacent to or within age-decreased genes (see green lines and 95% confidence intervals in B, D, F, H, J and L). The grey region in each panel outlines a 95% confidence interval for the average residual score among sequences assigned to the background set (which includes all sequences not within the foreground set). The analysis was repeated based upon foreground sets of varying size and selectivity (horizontal axis; see Figure S6 legend). A significant motif-expression association is indicated by non-overlap between 95% confidence intervals associated with foreground and background gene sets (denoted by blue bars located near the top or bottom of each panel). (PDF) [file pone.0033204.s017.pdf]

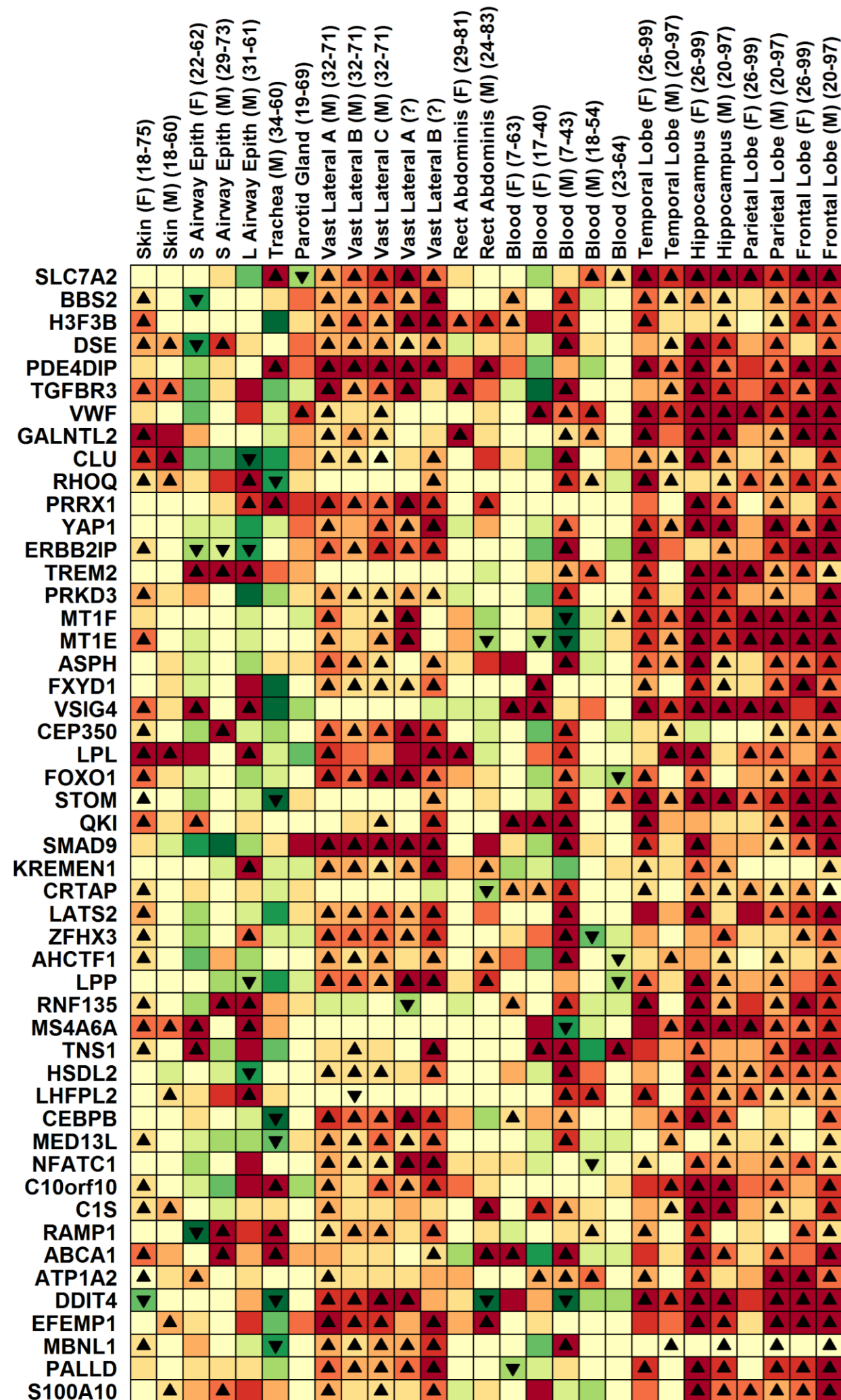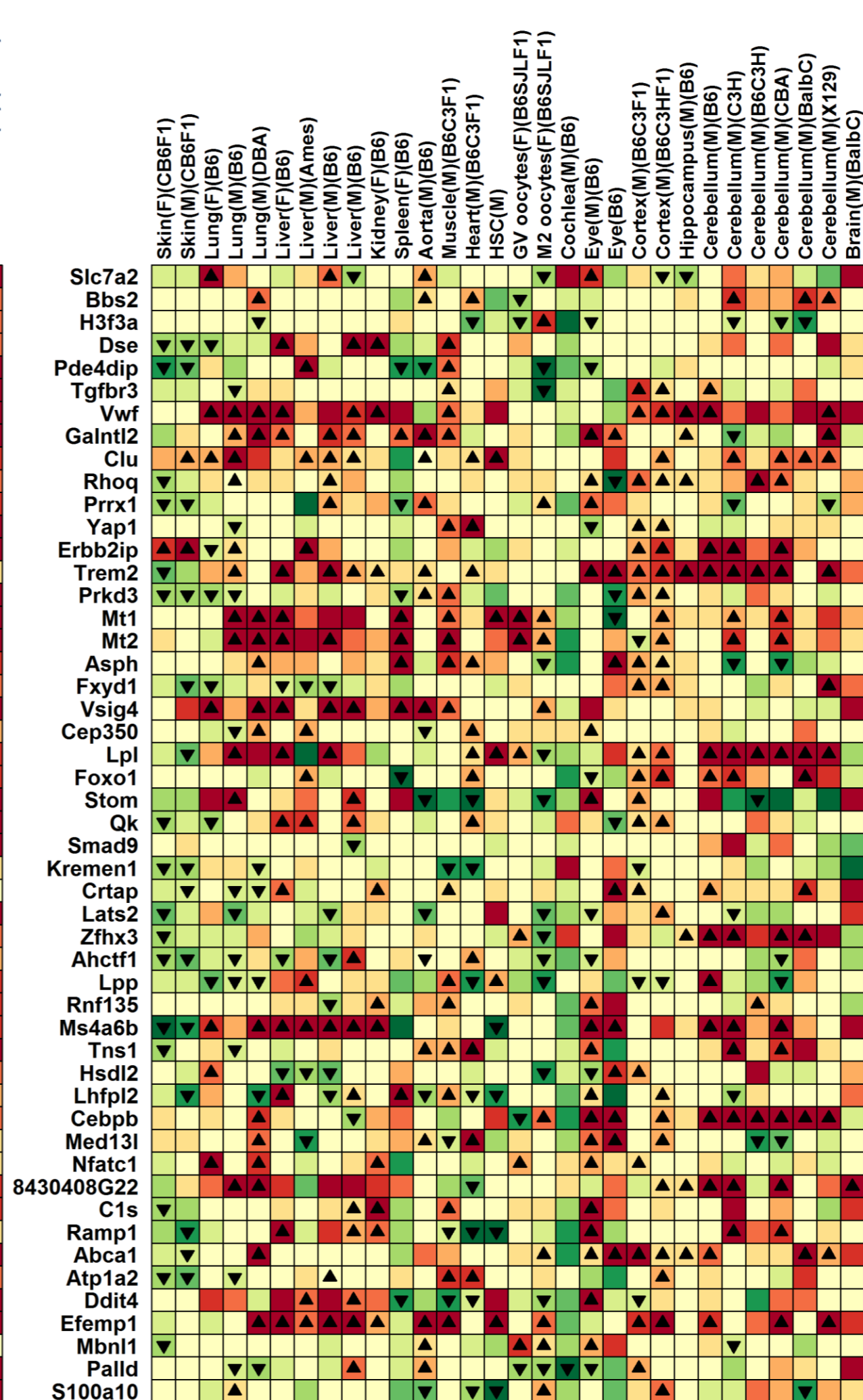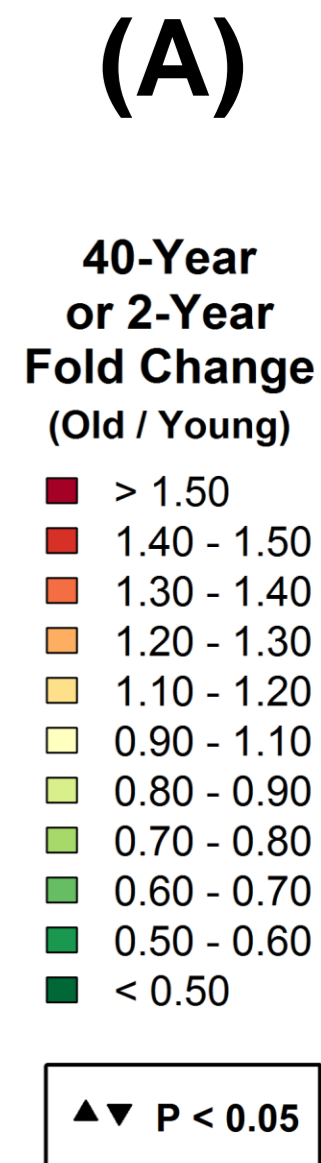

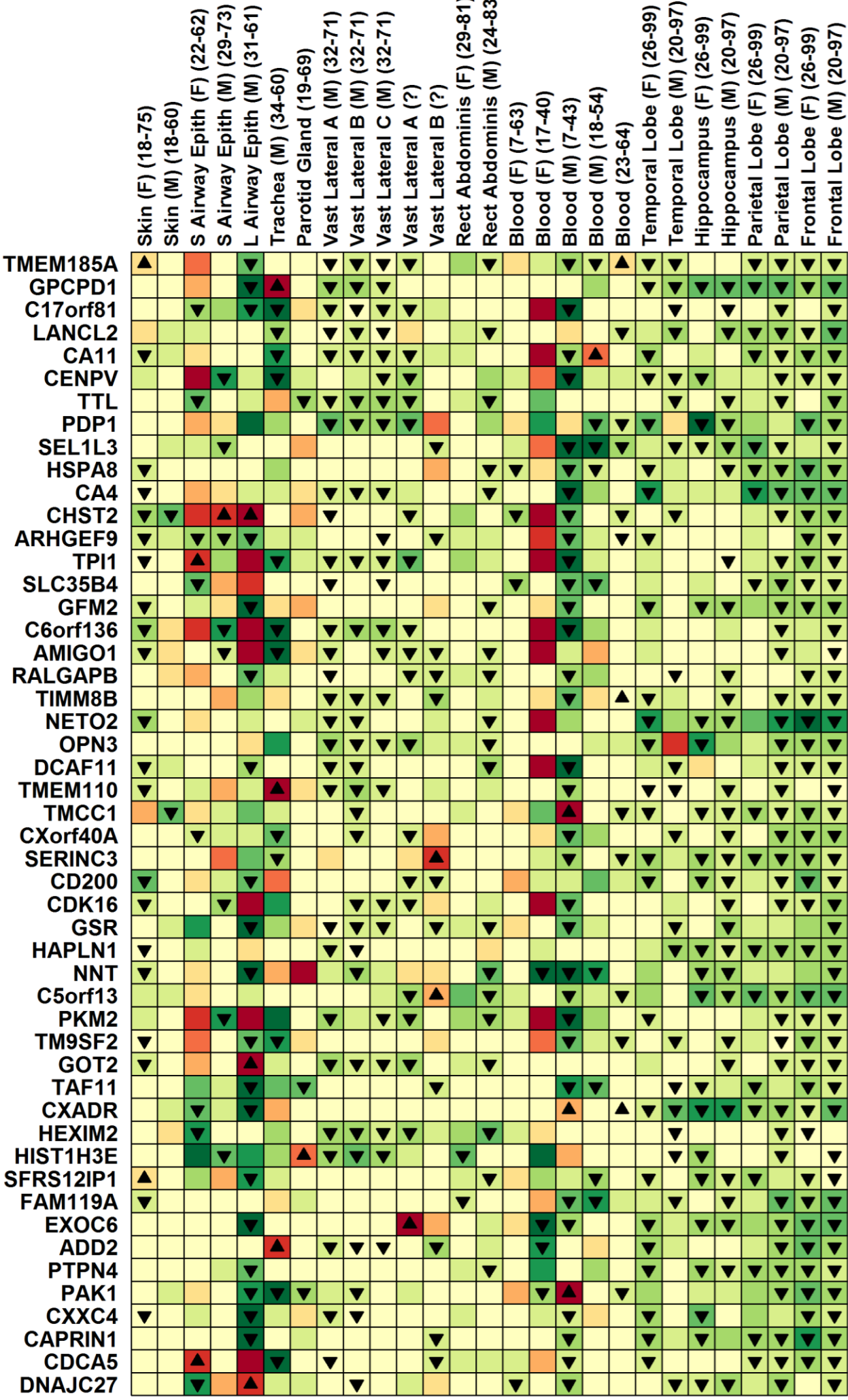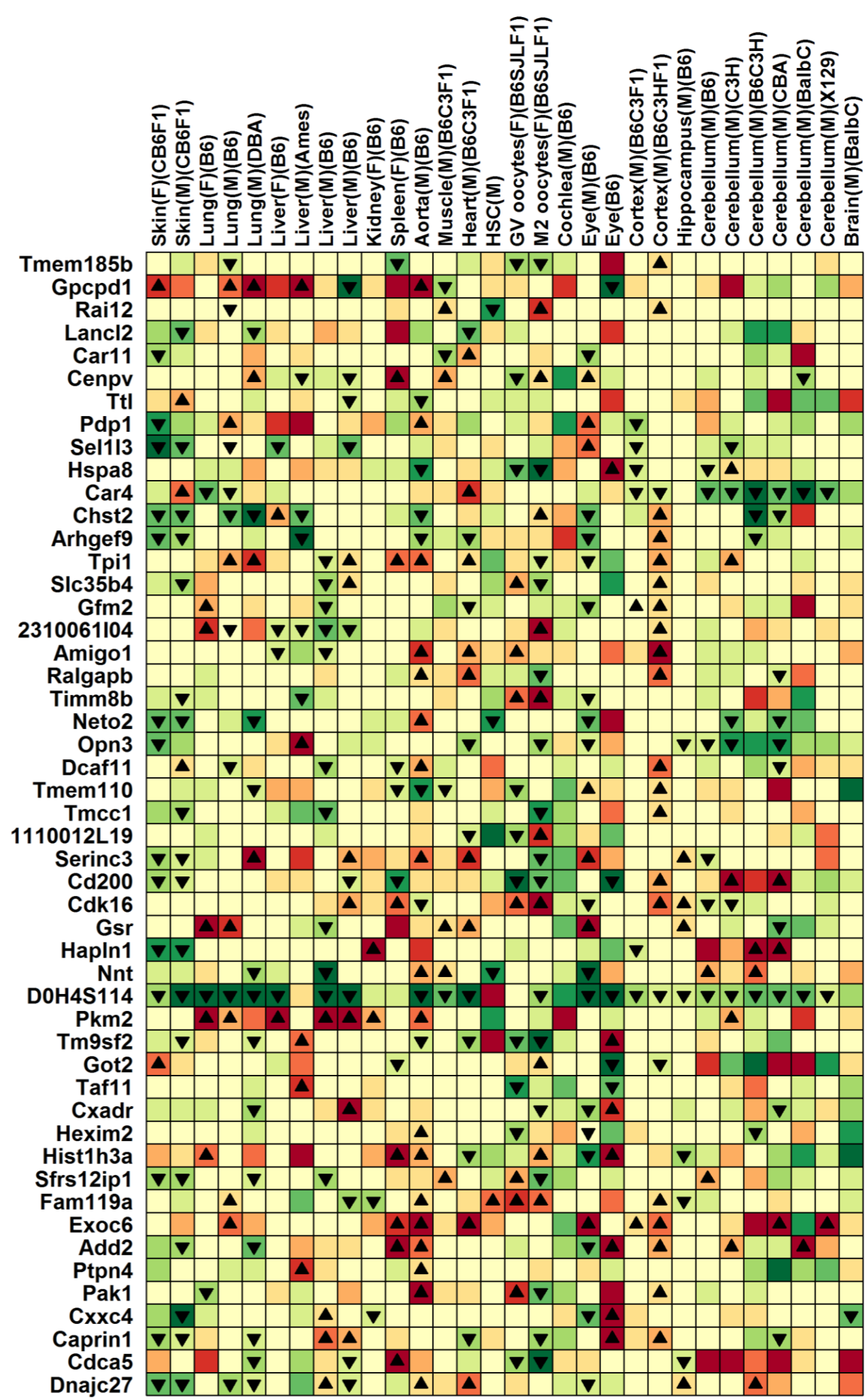

(B)

40-Year  
or 2-Year  
Fold Change  
(Old / Young)

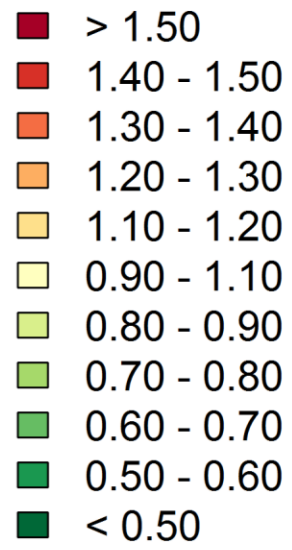

▲ ▼ P < 0.05

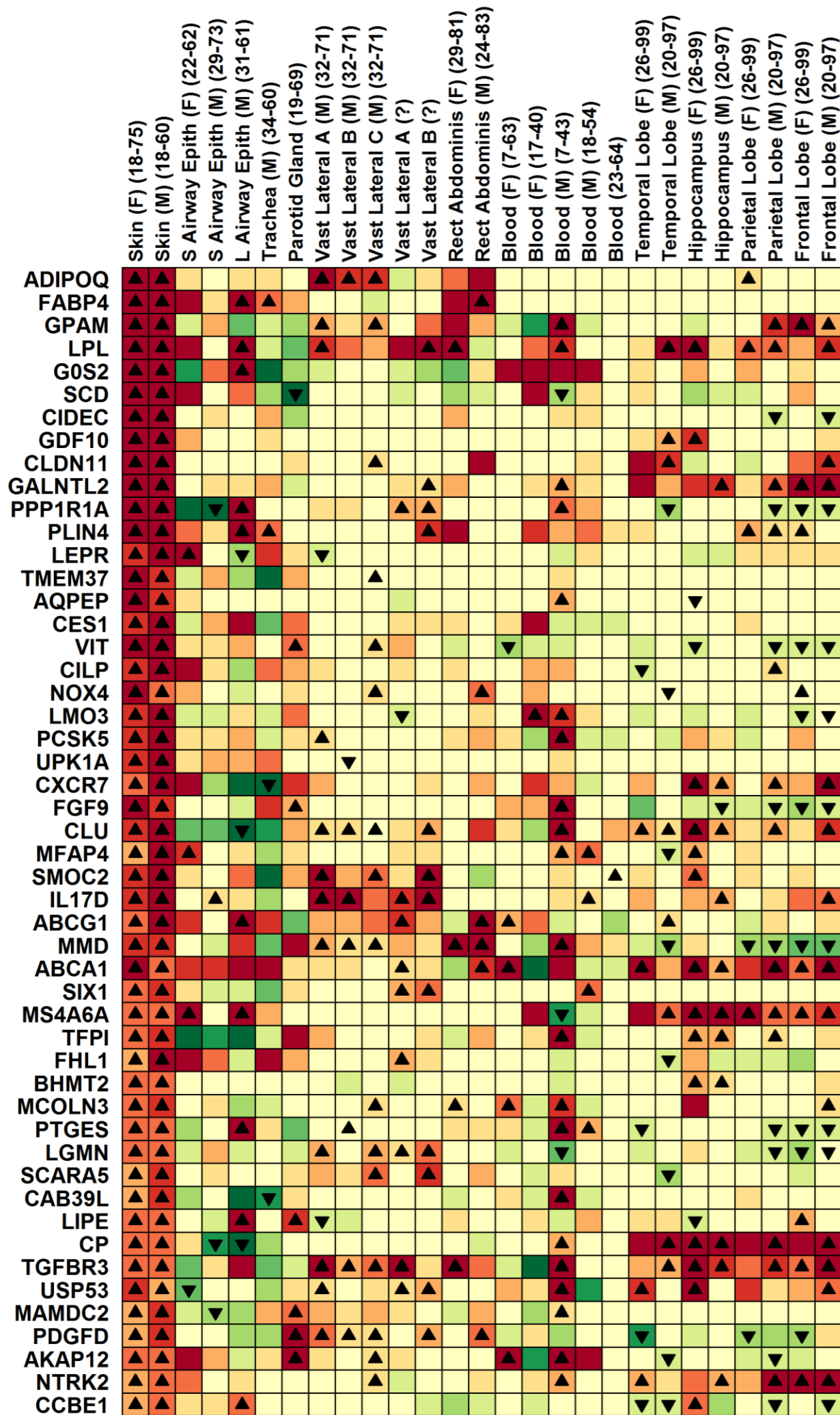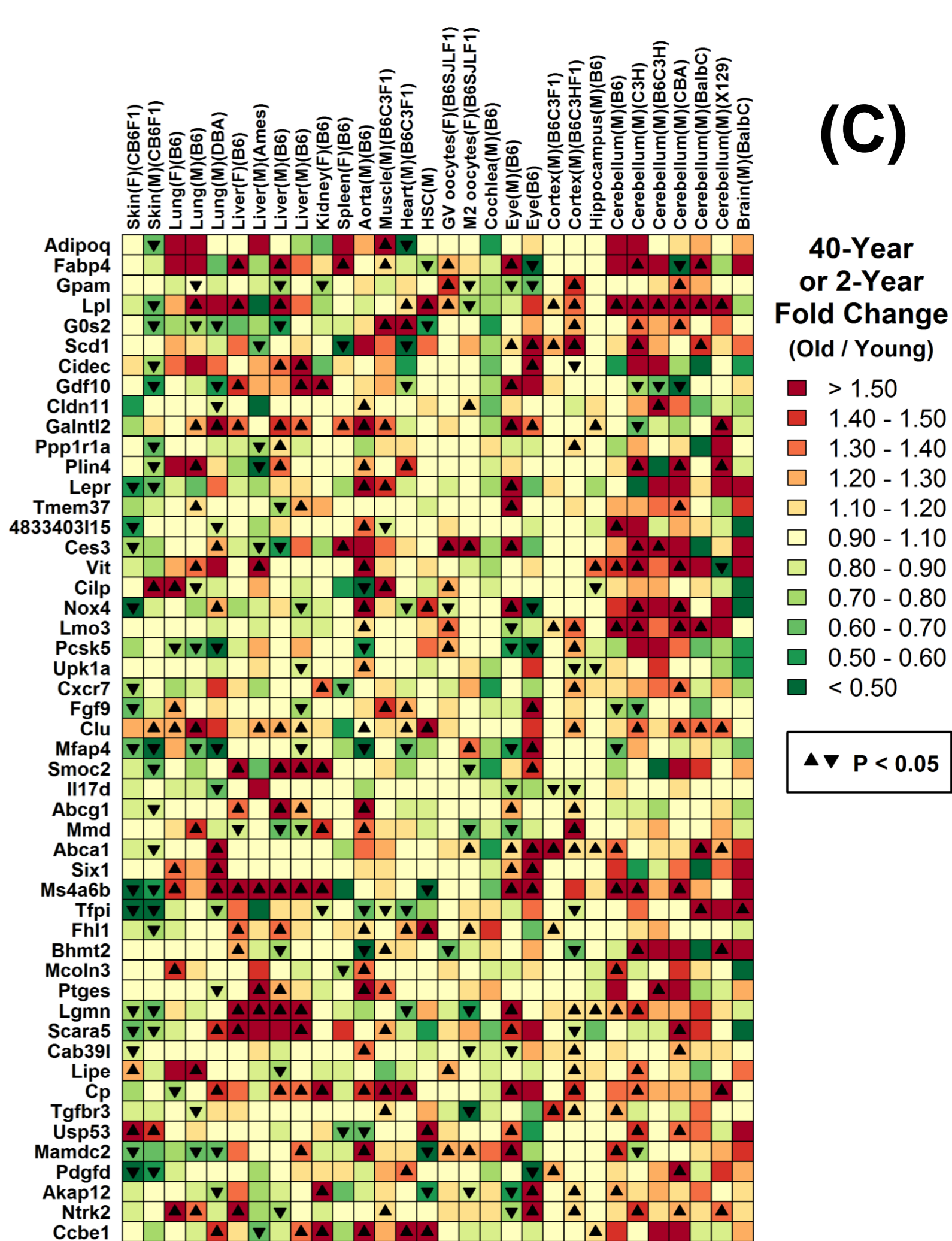

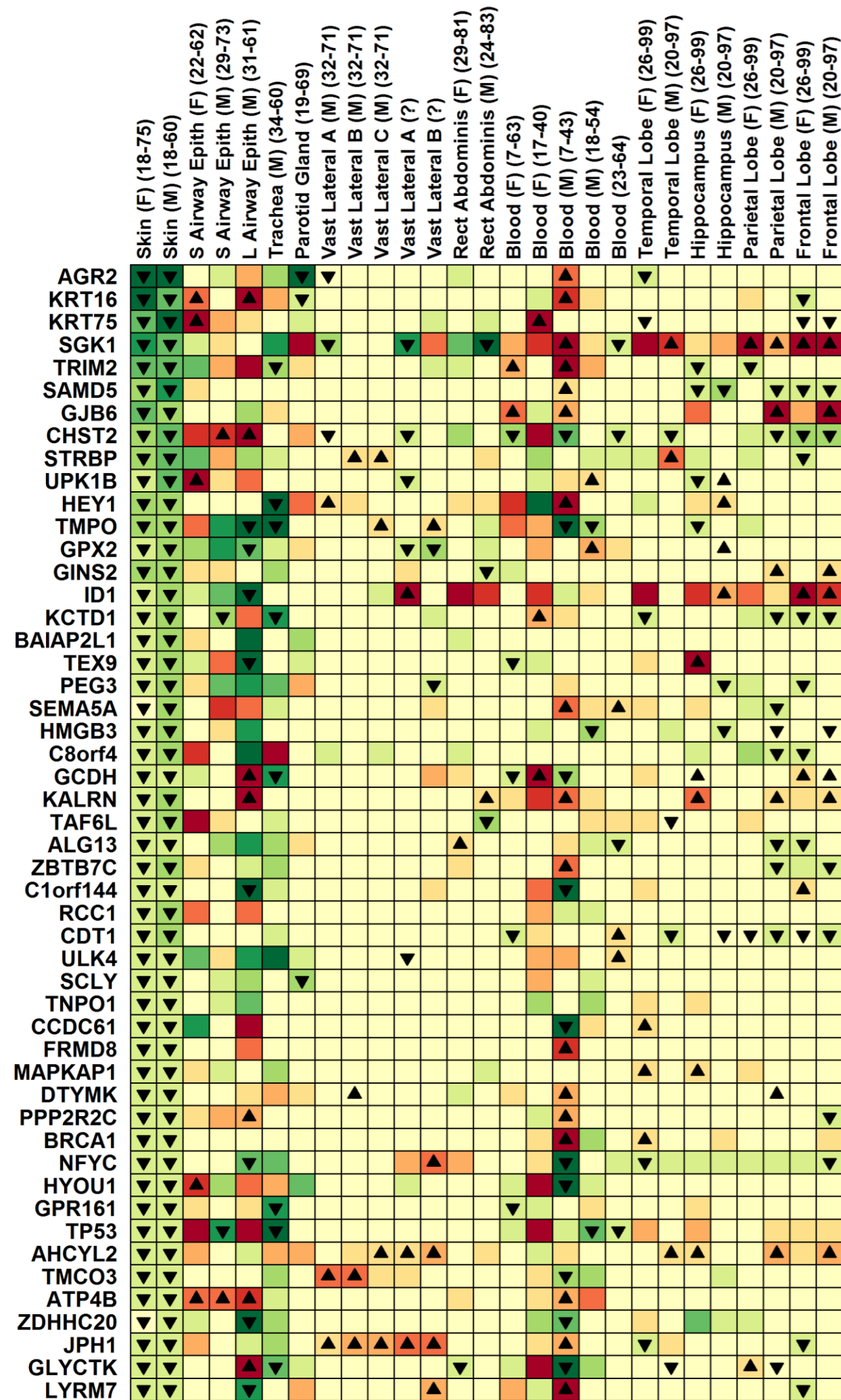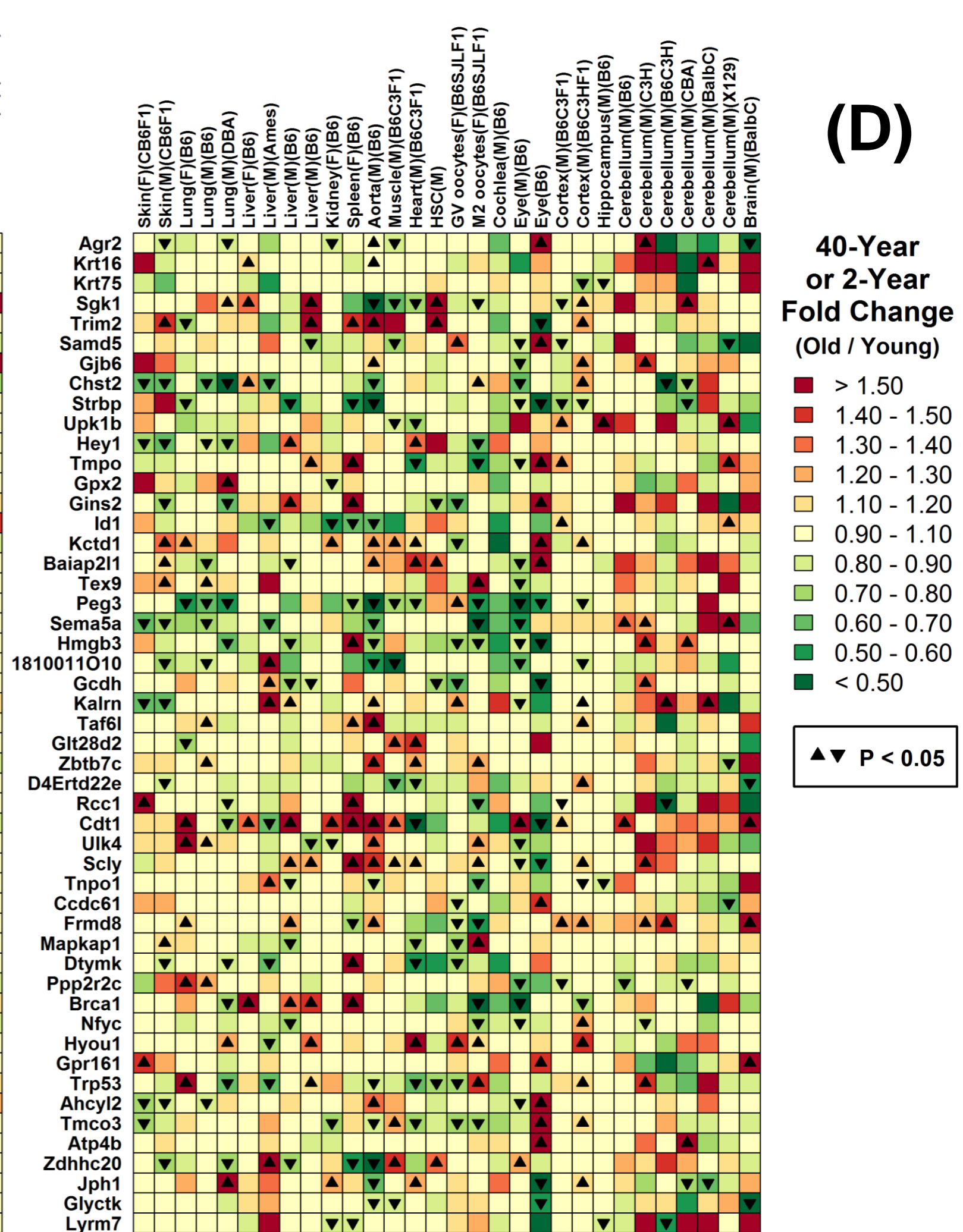

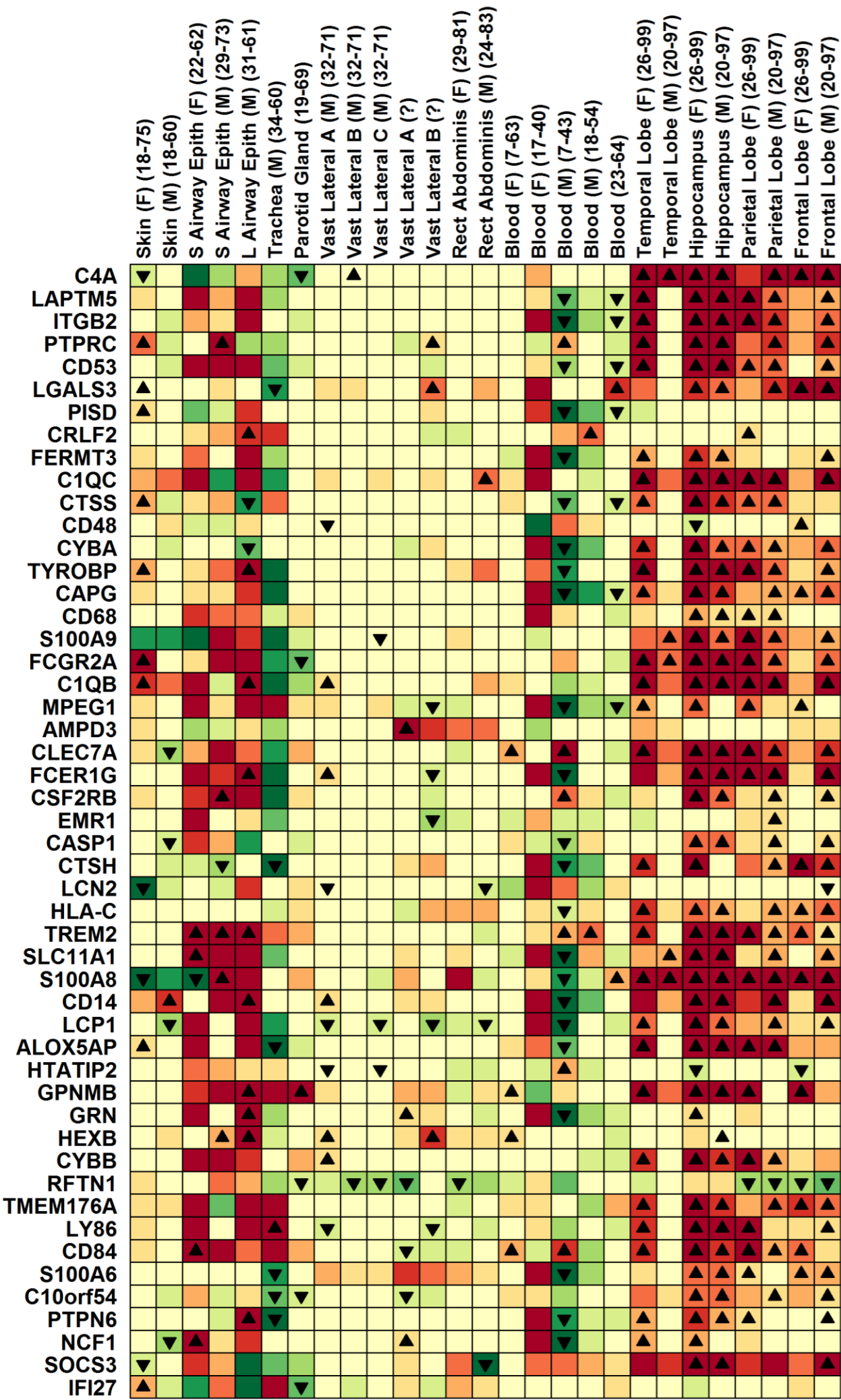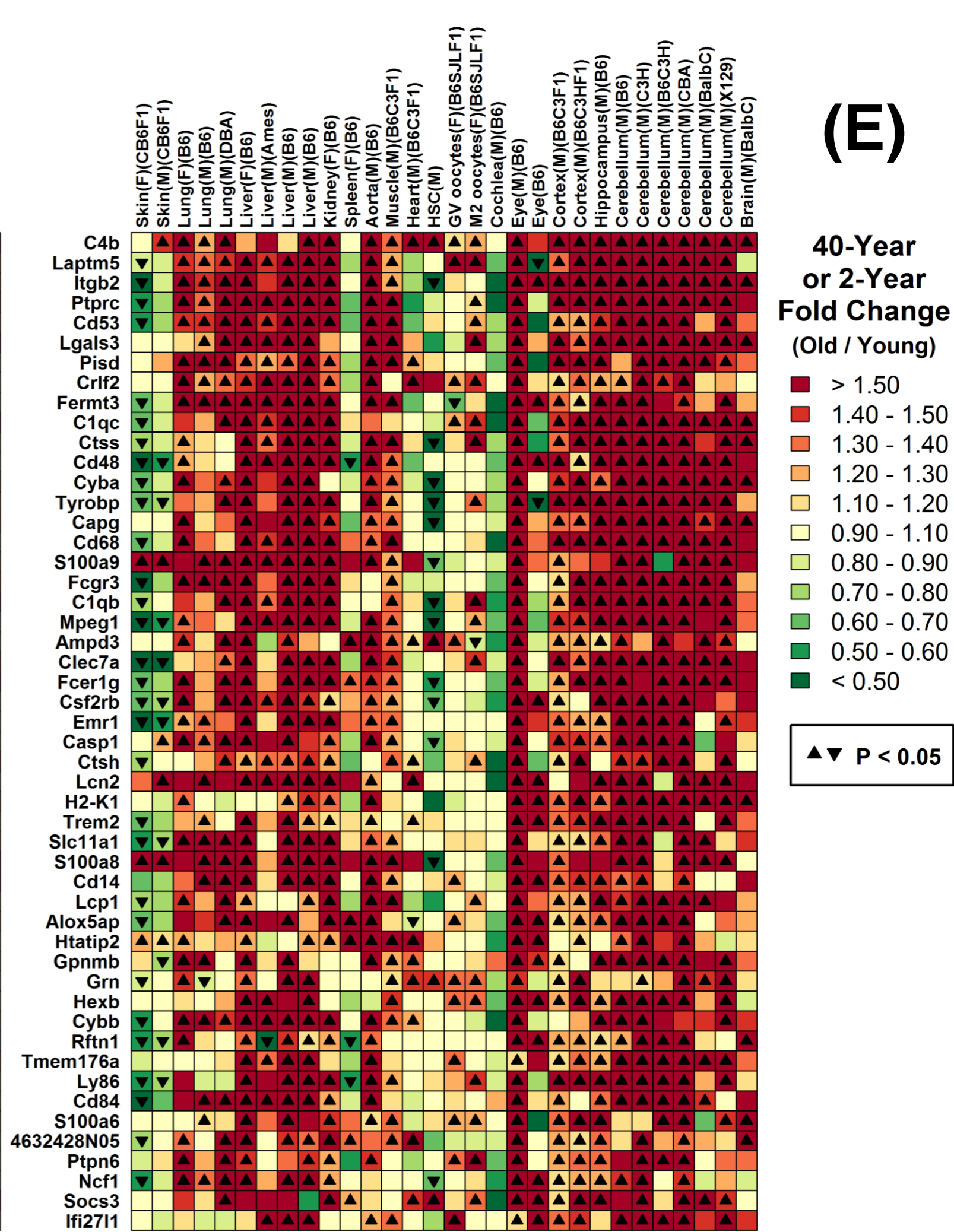

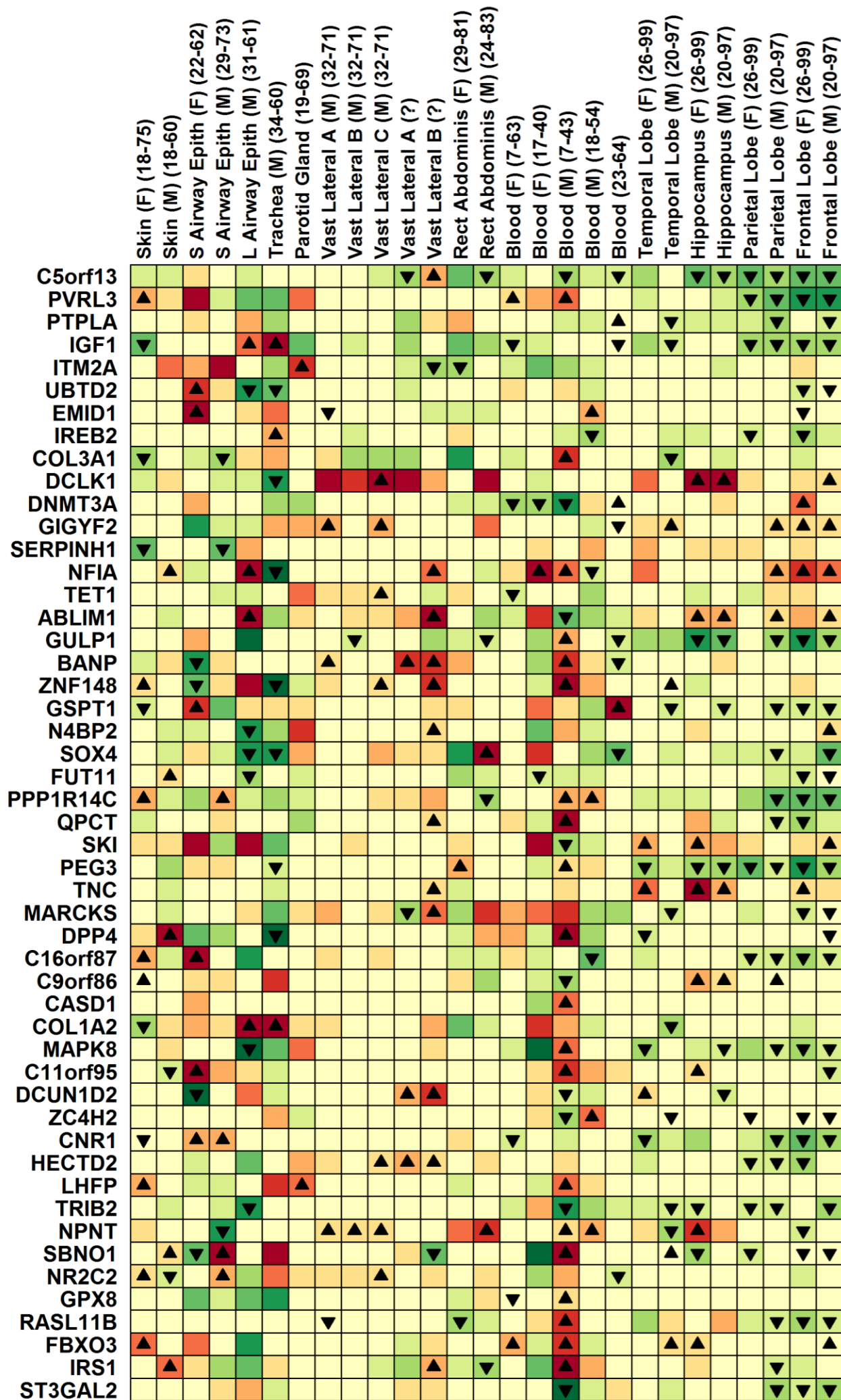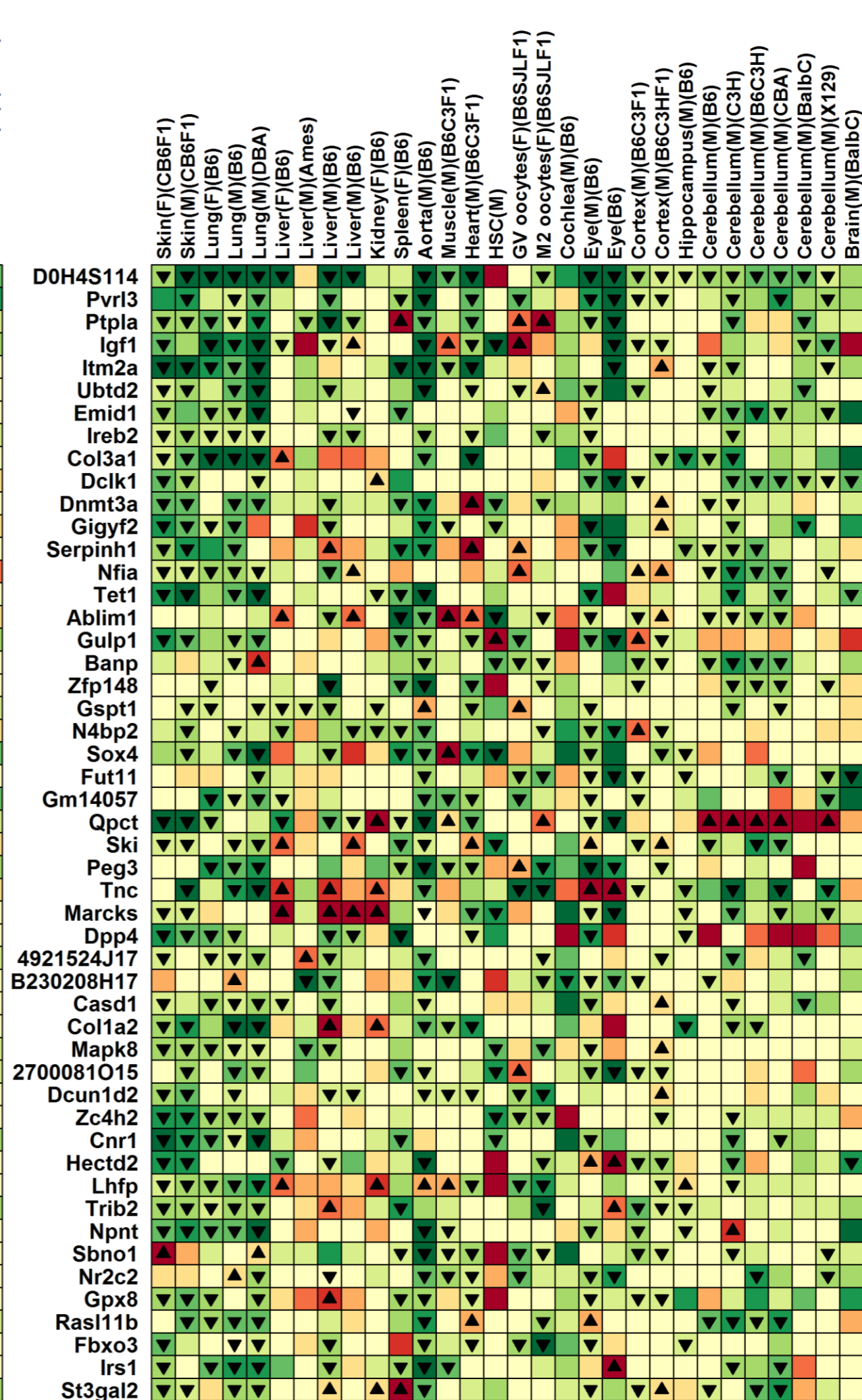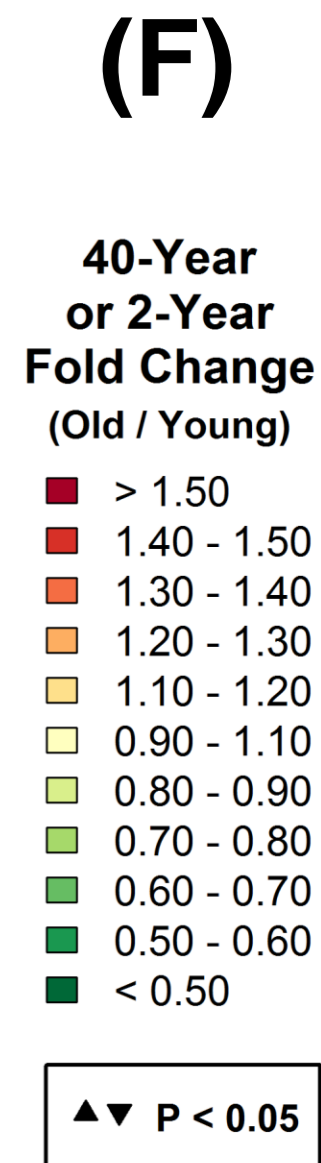

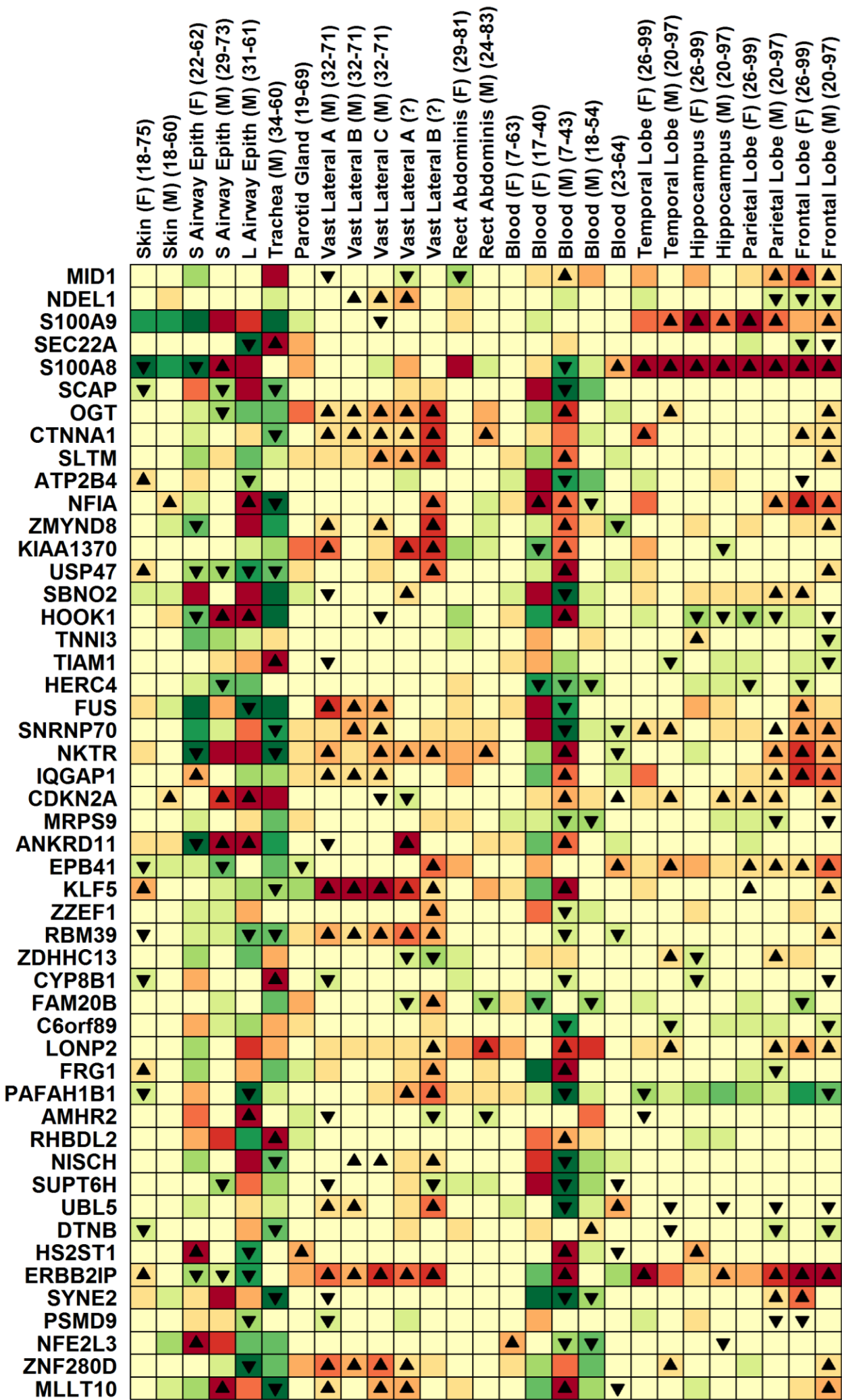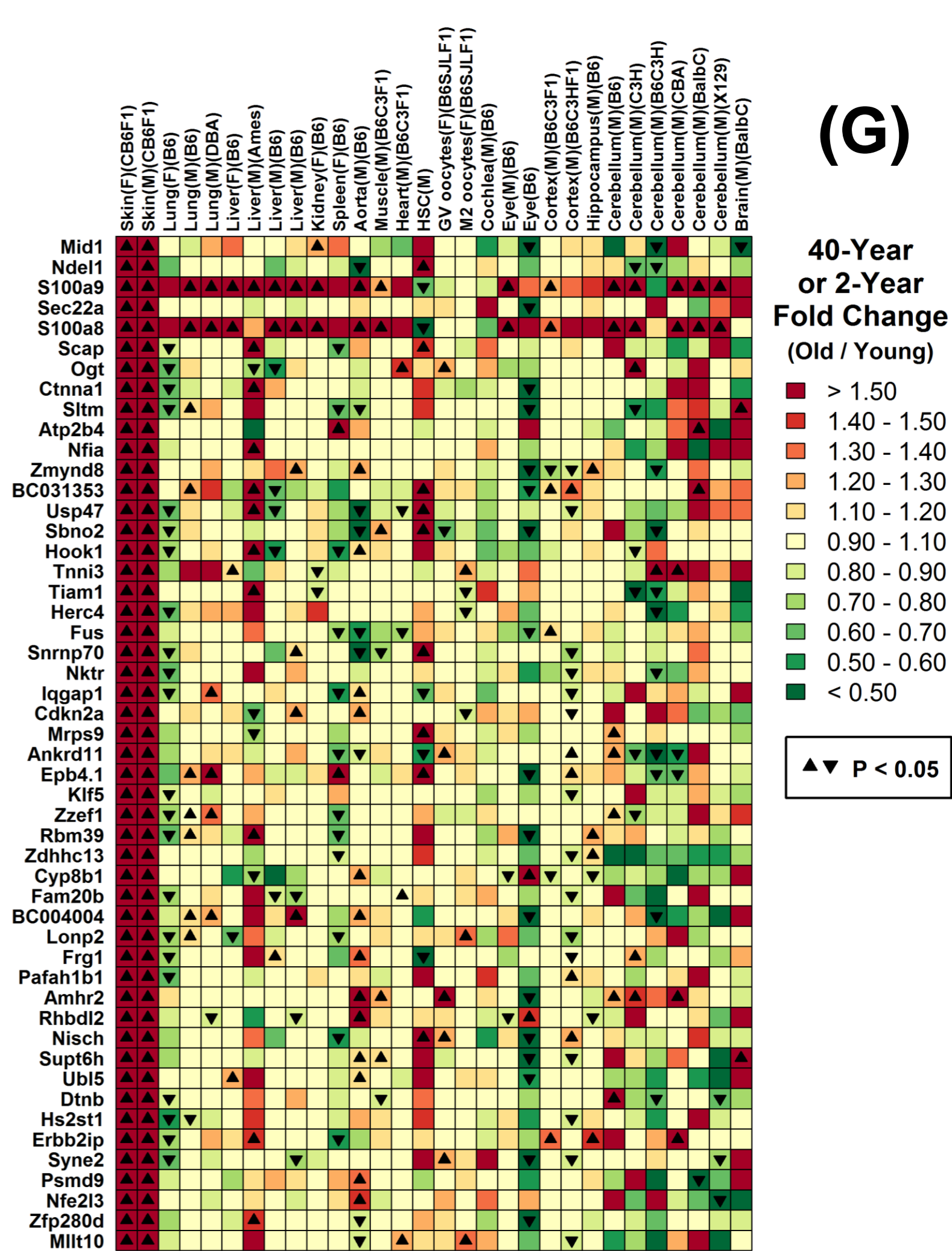

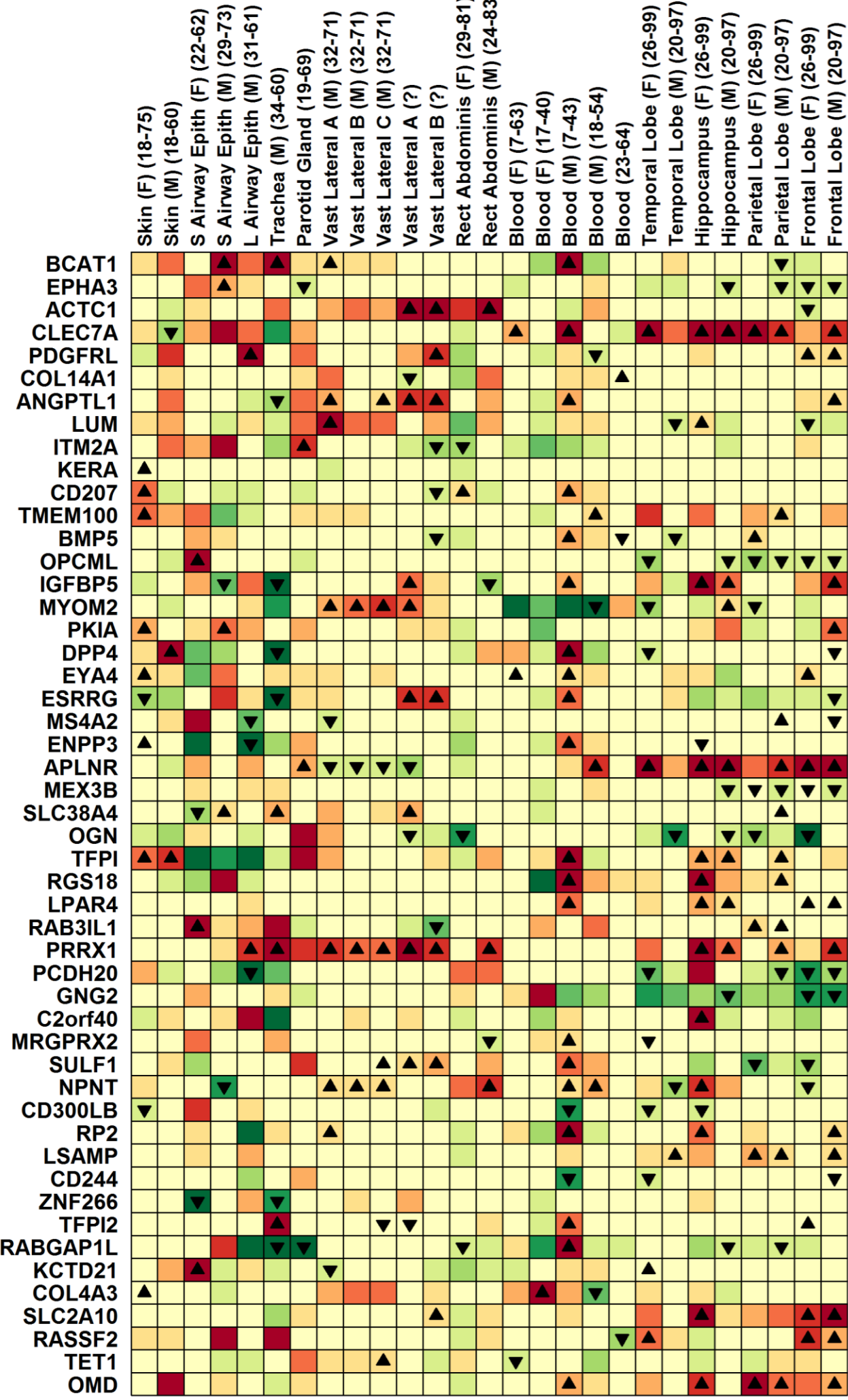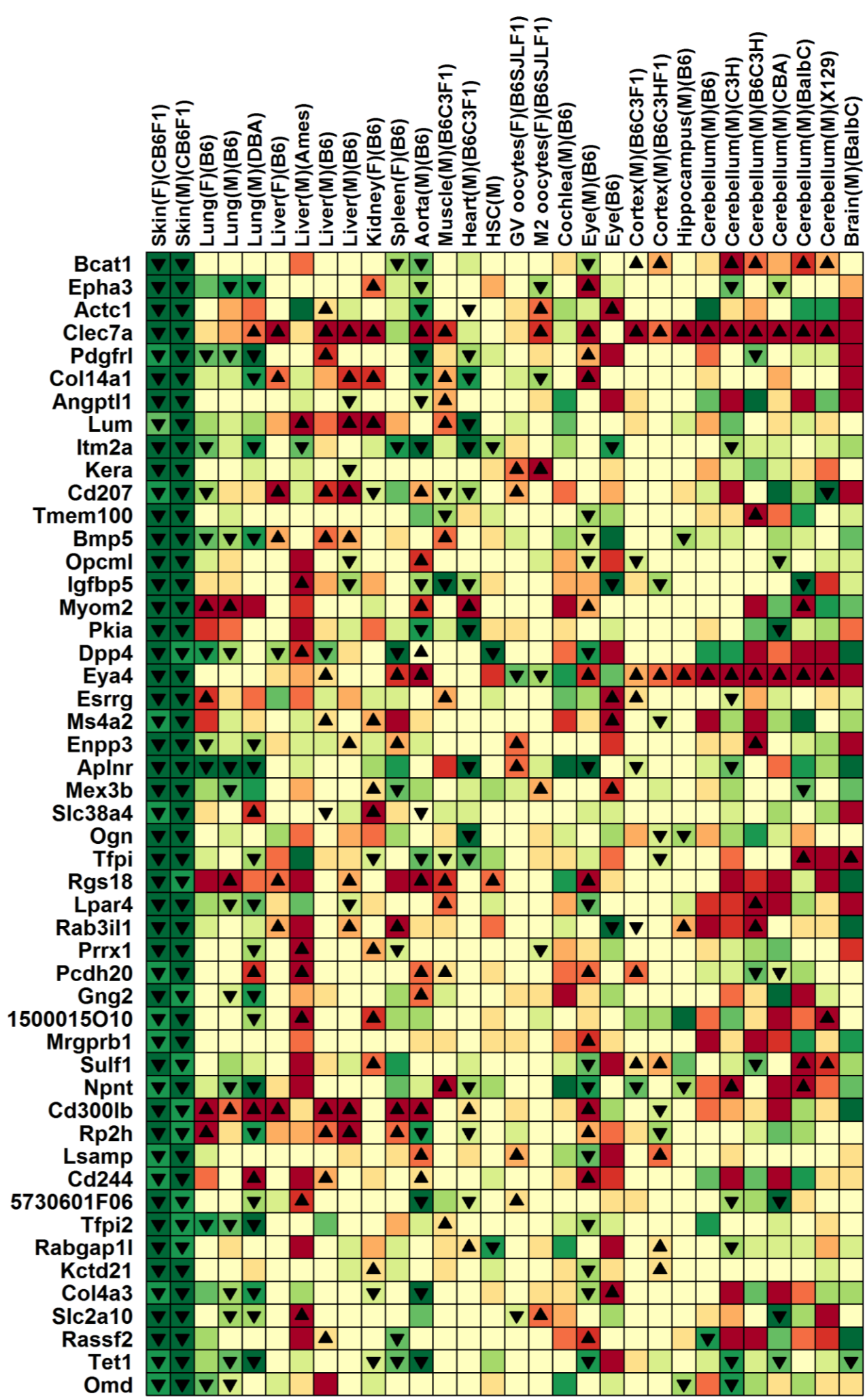

# (H)

## 40-Year or 2-Year Fold Change (Old / Young)

> 1.50

1.40 - 1.50

1.30 - 1.40

1.20 - 1.30

1.10 - 1.20

0.90 - 1.10

0.80 - 0.90

0.70 - 0.80

0.60 - 0.70

0.50 - 0.60

< 0.50

▲ ▼ P < 0.05

Supplement: Figure S18 — Ranked lists of genes altered by aging in both human and mouse tissues. Tables display age-associated expression patterns for ranked lists of the top 50 genes most strongly (A) increased by aging across all human tissues, (B) decreased by aging across all human tissues, (C) increased by aging in human skin (both sexes), (D) decreased by aging in human skin (both sexes), (E) increased by aging across all mouse tissues, (F) decreased by aging across all mouse tissues, (G) increased by aging in mouse skin (both sexes) and (H) decreased by aging in mouse skin (both sexes). In each figure (A)–(H), age-associated gene expression patterns in human tissues are displayed in the left panel, and age-associated gene expression patterns in mouse tissues are displayed in the right panel. Ranked lists include only orthologous genes that are shared between humans and mice (and thus may differ from those presented in Figures S1 and S10). In (A), (B), (E) and (F), genes have been ranked according to the total number of significant results observed for a given gene across human and mouse tissues (i.e., the total number of up-triangles per row or the total number of down-triangles per row). Tables (C), (D), (G) and (H) list genes most strongly regulated by aging in skin specifically, with genes first filtered to include only genes significantly altered by age in both sexes of a given species (P<0.05), and then ranked according to the estimated 40-year or 2-year fold-change (old/young; averaged between males and females). (PDF) [file pone.0033204.s018.pdf]

Percent Overlap (Age-regulated Genes)

# (A) Human

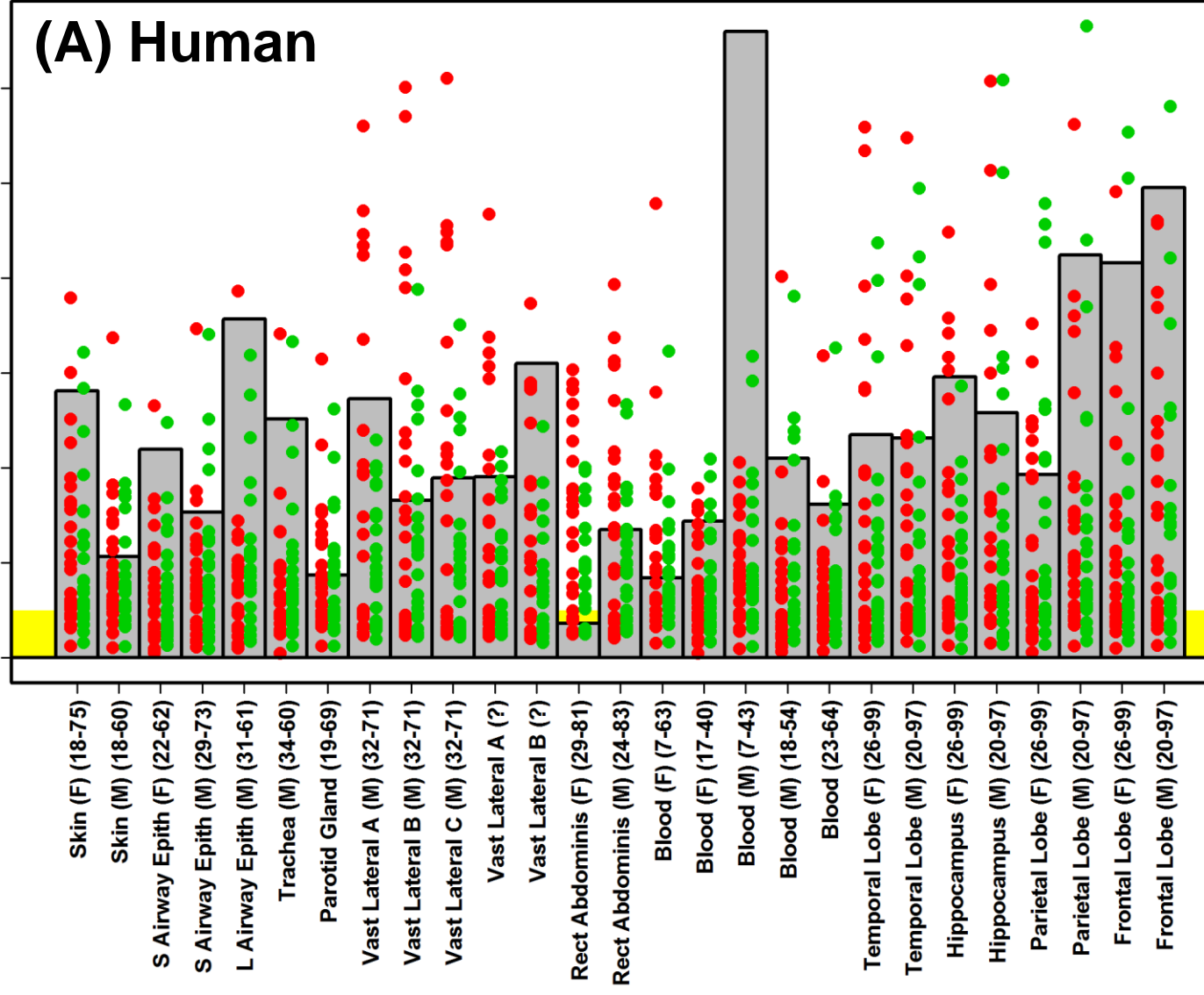

Percent Overlap (Age-regulated Genes)

(B) Mouse

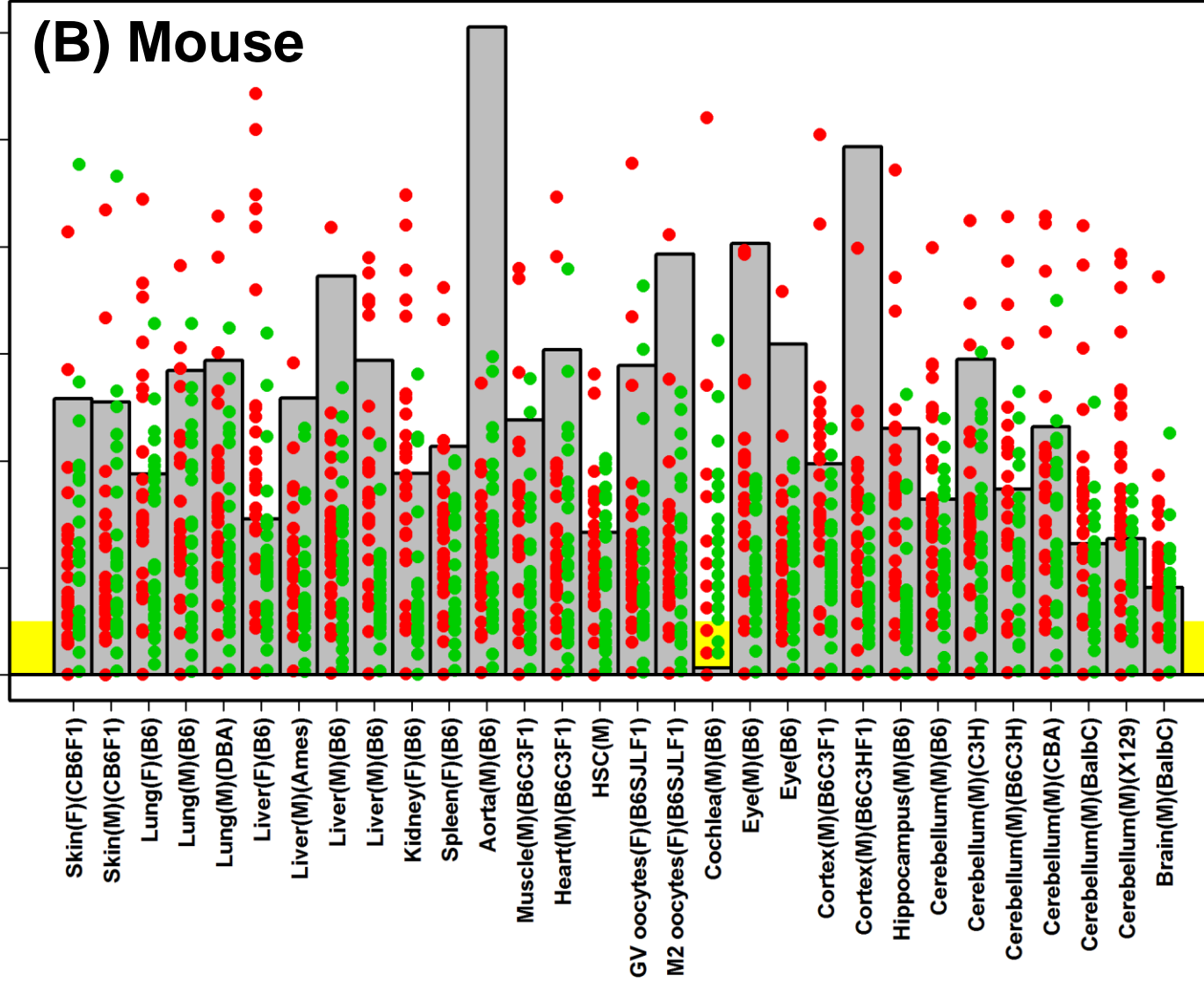

Supplement: Figure S20 — The overlap among sets of age-regulated genes derived from different datasets with young and old tissues is characteristically 10–15%. We calculated the pairwise overlap between sets of age-regulated genes derived from (A) 27 datasets that included young and old human tissue samples and (B) 30 datasets that included young and old mouse tissue samples. All human datasets considered in part (A) were generated from the same Affymetrix Human Genome U133 Plus 2.0 array platform (Table S1), and all mouse datasets considered in part (B) used the same Affymetrix Mouse Genome 430 2.0 Array platform (Table S2). Further details on each dataset included are provided in the Methods section. In both (A) and (B), datasets used to derive sets of age-regulated genes are listed along the horizontal axis. The height of each corresponding grey bar indicates the percentage of genes significantly altered by age (either increased or decreased), based upon a comparison-wise Type I error rate of P<0.05, such that approximately 5% of genes are expected to be age-regulated by chance (see yellow background behind grey bars). For each dataset, red and green dots indicate the percent overlap between the set of age-regulated genes identified from that dataset as compared to the others included in the same figure. Percent overlap is defined as 100× (x/y), where x is the number of unique genes significantly altered by age (in the same direction) in two separate datasets, and y is the number of unique genes significantly altered by age in the reference dataset listed along the horizontal axis. Red dots indicate overlap with respect to sets of age-increased genes, while green dots indicate overlap with respect to sets of age-decreased genes. In (A), we evaluated gene set overlap among all 351 pairwise combinations among the 27 human datasets, and on average, overlap between sets of age-regulated genes was 11.4%, with a minimum of 0.50% and a maximum of 66.5%. In (B), we evaluated gene set overlap amo [file pone.0033204.s020.pdf]
