# Supplementary material for: Meta-Profiles of Gene Expression during Aging: Limited Similarities between Mouse and Human and an Unexpectedly Decreased Inflammatory Signature
Source: PLoS One. 2012 Mar 7;7(3):e33204. doi: 10.1371/journal.pone.0033204 (PMC3296693; doi:10.1371/journal.pone.0033204)
Supplement: Figure S11 — Gene ontology biological process terms associated with mouse tail skin aging (CB6F1 strain). Genes regulated by aging in mouse tail skin were analyzed to identify significantly over-represented gene ontology (GO) biological process terms (P<0.05; Fisher's exact test). The analysis was performed based upon (A) a set of 2633 mouse genes significantly increased by aging in females (P<0.05), (B) a set of 3134 mouse genes significantly decreased by aging in females (P<0.05), (C) a set of 2509 mouse genes significantly increased by aging in males (P<0.05) and (D) a set of 3208 mouse genes significantly decreased by aging in males (P<0.05). The font color of each GO term in the left margin corresponds to the degree to which that term was overrepresented (P<0.05, black font; P<0.01, blue font; P<0.001, red font). For each GO term, colors within the chart denote the average 2-year fold change (old/young) among genes associated with that GO term. The number of genes associated with each GO term is indicated in brackets (this total only includes genes that are among the 2633, 3134, 2509 or 3208 genes analyzed in parts A, B, C and D, respectively). Filled triangles indicate whether average fold-change estimates among these genes are significantly high (up-triangle; P<0.05) or significantly low (down-triangle; P<0.05). Triangles are displayed only if significant p-values were obtained from each of three separate tests evaluating whether the average fold-change estimate (or distribution of estimates) is significantly high or low (i.e., one-sample t-test, exact binomial proportion test and Wilcoxon signed rank test; triangles denote P<0.05 in each test). GO terms have been clustered based upon a similarity metric that is proportional to the number of shared ancestral terms in the GO hierarchy. (PDF) [file pone.0033204.s011.pdf]

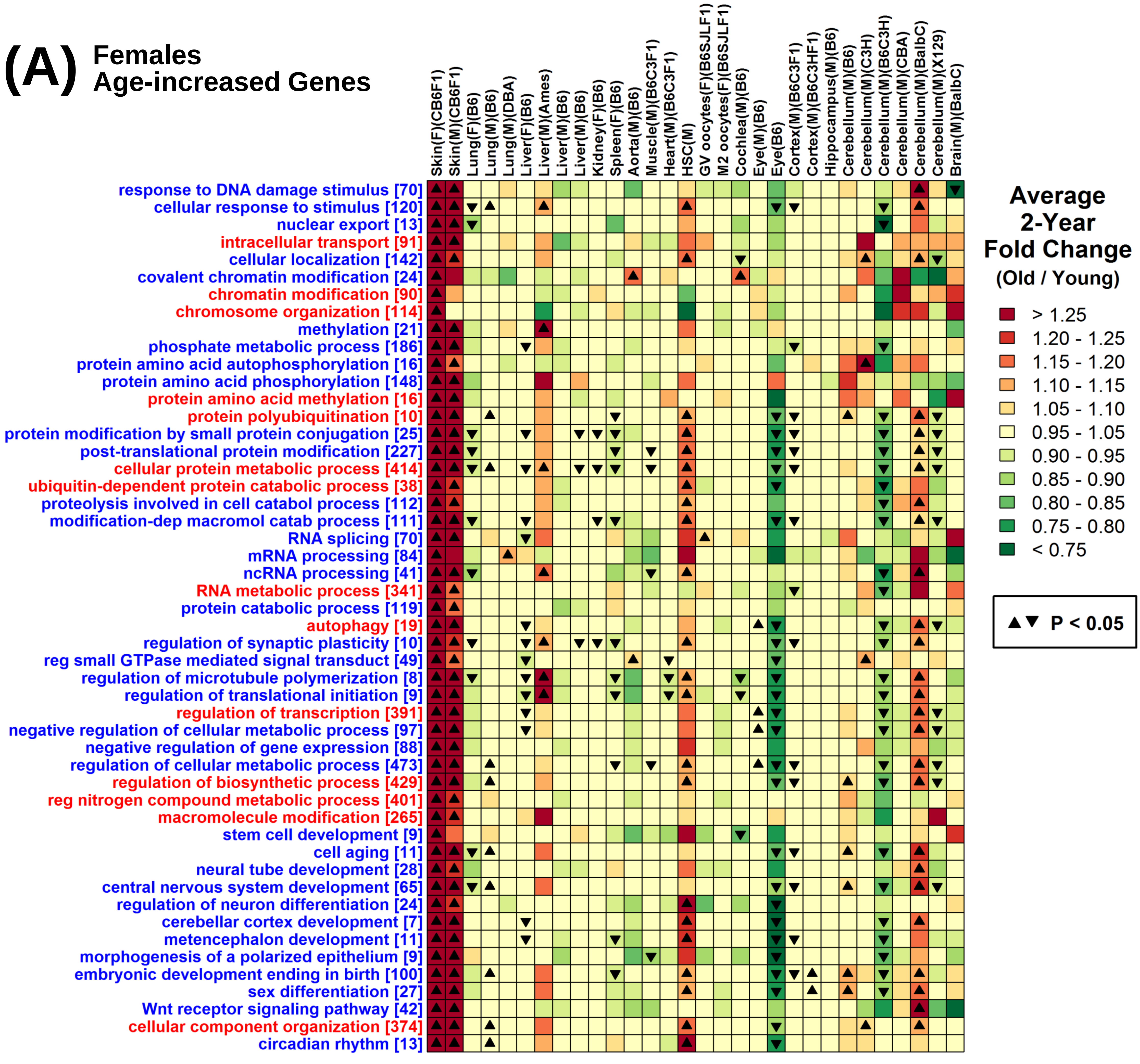
(A) Females
Age-increased Genes
Skin(F)(CB6F1)
Skin(M)(CB6F1)
Lung(F)(B6)
Lung(M)(B6)
Lung(M)(DBA)
Liver(F)(B6)
Liver(M)(Ames)
Liver(M)(B6)
Liver(M)(B6)
Kidney(F)(B6)
Spleen(F)(B6)
Aorta(M)(B6)
Muscle(M)(B6C3F1)
Heart(M)(B6C3F1)
HSC(M)
GV oocytes(F)(B6SJLF1)
M2 oocytes(F)(B6SJLF1)
Cochlea(M)(B6)
Eye(M)(B6)
Eye(B6)
Cortex(M)(B6C3F1)
Cortex(M)(B6C3HF1)
Hippocampus(M)(B6)
Cerebellum(M)(B6)
Cerebellum(M)(C3H)
Cerebellum(M)(B6C3H)
Cerebellum(M)(CBA)
Cerebellum(M)(BalbC)
Cerebellum(M)(X129)
Brain(M)(BalbC)
response to DNA damage stimulus [70]
cellular response to stimulus [120]
nuclear export [13]
intracellular transport [91]
cellular localization [142]
covalent chromatin modification [24]
chromatin modification [90]
chromosome organization [114]
methylation [21]
phosphate metabolic process [186]
protein amino acid autophosphorylation [16]
protein amino acid phosphorylation [148]
protein amino acid methylation [16]
protein polyubiquitination [10]
protein modification by small protein conjugation [25]
post-translational protein modification [227]
cellular protein metabolic process [414]
ubiquitin-dependent protein catabolic process [38]
proteolysis involved in cell catabol process [112]
modification-dep macromol catab process [111]
RNA splicing [70]
mRNA processing [84]
ncRNA processing [41]
RNA metabolic process [341]
protein catabolic process [119]
autophagy [19]
regulation of synaptic plasticity [10]
reg small GTPase mediated signal transduct [49]
regulation of microtubule polymerization [8]
regulation of translational initiation [9]
regulation of transcription [391]
negative regulation of cellular metabolic process [97]
negative regulation of gene expression [88]
regulation of cellular metabolic process [473]
regulation of biosynthetic process [429]
reg nitrogen compound metabolic process [401]
macromolecule modification [265]
stem cell development [9]
cell aging [11]
neural tube development [28]
central nervous system development [65]
regulation of neuron differentiation [24]
cerebellar cortex development [7]
metencephalon development [11]
morphogenesis of a polarized epithelium [9]
embryonic development ending in birth [100]
sex differentiation [27]
Wnt receptor signaling pathway [42]
cellular component organization [374]
circadian rhythm [13]
Average
2-Year
Fold Change
(Old / Young)
> 1.25
1.20 - 1.25
1.15 - 1.20
1.10 - 1.15
1.05 - 1.10
0.95 - 1.05
0.90 - 0.95
0.85 - 0.90
0.80 - 0.85
0.75 - 0.80
< 0.75
▲▼ P < 0.05

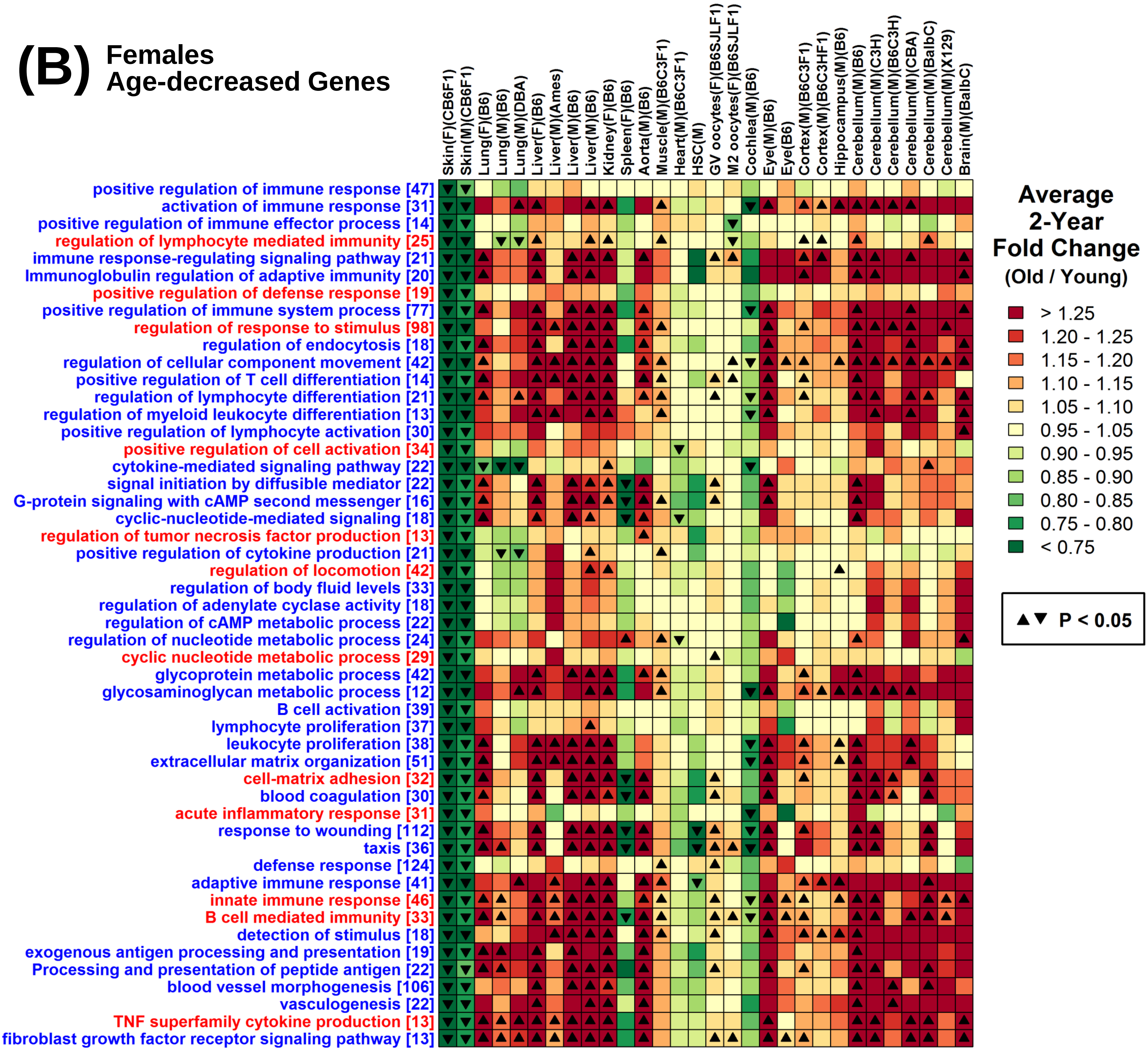
(B) Females
Age-decreased Genes
Skin(F)(CB6F1)
Skin(M)(CB6F1)
Lung(F)(B6)
Lung(M)(B6)
Lung(M)(DBA)
Liver(F)(B6)
Liver(M)(Ames)
Liver(M)(B6)
Liver(M)(B6)
Kidney(F)(B6)
Spleen(F)(B6)
Aorta(M)(B6)
Muscle(M)(B6C3F1)
Heart(M)(B6C3F1)
HSC(M)
GV oocytes(F)(B6SJLF1)
M2 oocytes(F)(B6SJLF1)
Cochlea(M)(B6)
Eye(M)(B6)
Eye(B6)
Cortex(M)(B6C3F1)
Cortex(M)(B6C3HF1)
Hippocampus(M)(B6)
Cerebellum(M)(B6)
Cerebellum(M)(C3H)
Cerebellum(M)(B6C3H)
Cerebellum(M)(CBA)
Cerebellum(M)(BalbC)
Cerebellum(M)(X129)
Brain(M)(BalbC)
positive regulation of immune response [47]
activation of immune response [31]
positive regulation of immune effector process [14]
regulation of lymphocyte mediated immunity [25]
immune response-regulating signaling pathway [21]
Immunoglobulin regulation of adaptive immunity [20]
positive regulation of defense response [19]
positive regulation of immune system process [77]
regulation of response to stimulus [98]
regulation of endocytosis [18]
regulation of cellular component movement [42]
positive regulation of T cell differentiation [14]
regulation of lymphocyte differentiation [21]
regulation of myeloid leukocyte differentiation [13]
positive regulation of lymphocyte activation [30]
positive regulation of cell activation [34]
cytokine-mediated signaling pathway [22]
signal initiation by diffusible mediator [22]
G-protein signaling with cAMP second messenger [16]
cyclic-nucleotide-mediated signaling [18]
regulation of tumor necrosis factor production [13]
positive regulation of cytokine production [21]
regulation of locomotion [42]
regulation of body fluid levels [33]
regulation of adenylate cyclase activity [18]
regulation of cAMP metabolic process [22]
regulation of nucleotide metabolic process [24]
cyclic nucleotide metabolic process [29]
glycoprotein metabolic process [42]
glycosaminoglycan metabolic process [12]
B cell activation [39]
lymphocyte proliferation [37]
leukocyte proliferation [38]
extracellular matrix organization [51]
cell-matrix adhesion [32]
blood coagulation [30]
acute inflammatory response [31]
response to wounding [112]
taxis [36]
defense response [124]
adaptive immune response [41]
innate immune response [46]
B cell mediated immunity [33]
detection of stimulus [18]
exogenous antigen processing and presentation [19]
Processing and presentation of peptide antigen [22]
blood vessel morphogenesis [106]
vasculogenesis [22]
TNF superfamily cytokine production [13]
fibroblast growth factor receptor signaling pathway [13]
Average
2-Year
Fold Change
(Old / Young)
> 1.25
1.20 - 1.25
1.15 - 1.20
1.10 - 1.15
1.05 - 1.10
0.95 - 1.05
0.90 - 0.95
0.85 - 0.90
0.80 - 0.85
0.75 - 0.80
< 0.75
▲▼ P < 0.05

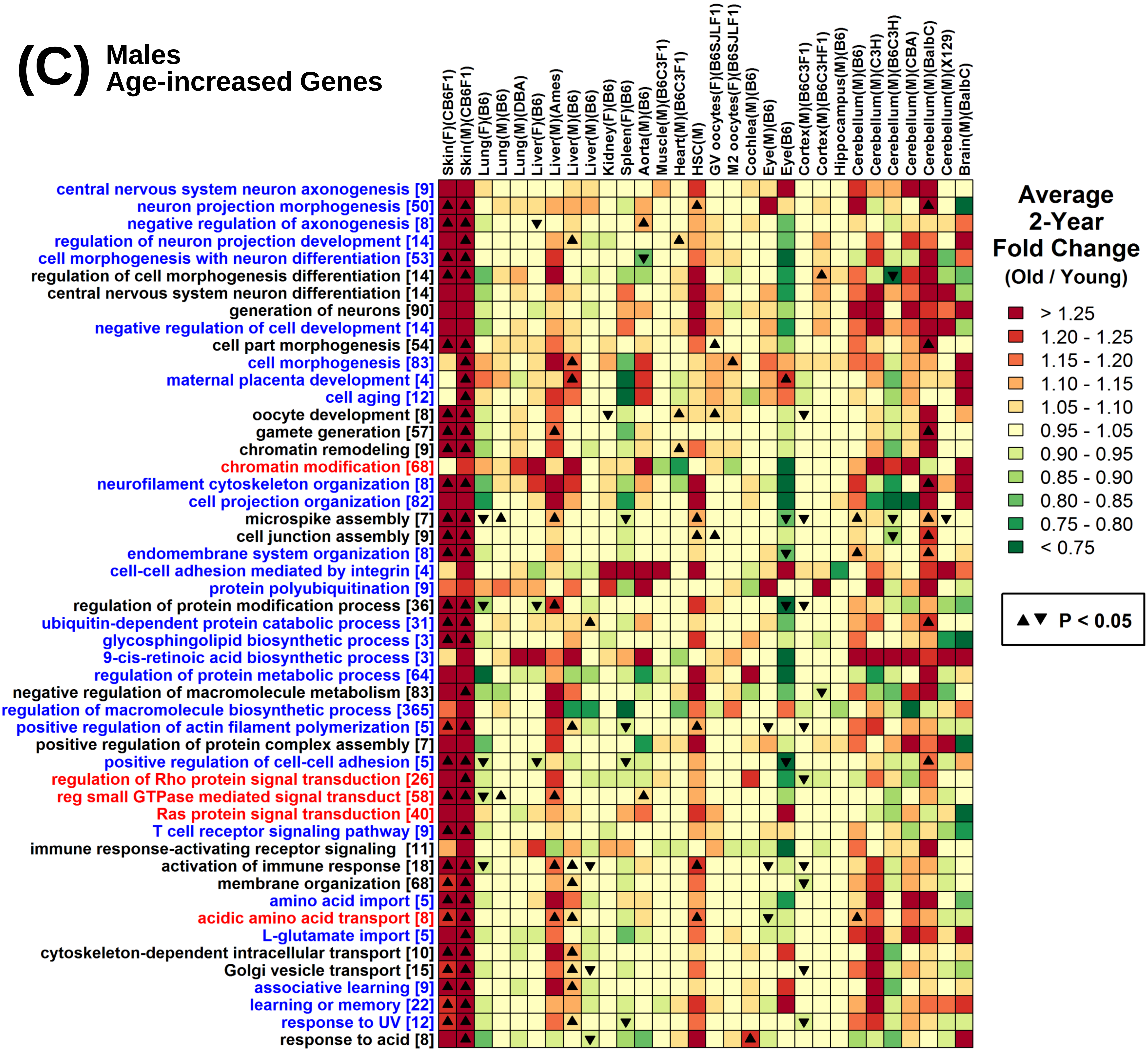
(C)
Males
Age-increased Genes
Skin(F)(CB6F1)
Skin(M)(CB6F1)
Lung(F)(B6)
Lung(M)(B6)
Lung(M)(DBA)
Liver(F)(B6)
Liver(M)(Ames)
Liver(M)(B6)
Liver(M)(B6)
Kidney(F)(B6)
Spleen(F)(B6)
Aorta(M)(B6)
Muscle(M)(B6C3F1)
Heart(M)(B6C3F1)
HSC(M)
GV oocytes(F)(B6SJLF1)
M2 oocytes(F)(B6SJLF1)
Cochlea(M)(B6)
Eye(M)(B6)
Eye(B6)
Cortex(M)(B6C3F1)
Cortex(M)(B6C3HF1)
Hippocampus(M)(B6)
Cerebellum(M)(B6)
Cerebellum(M)(C3H)
Cerebellum(M)(B6C3H)
Cerebellum(M)(CBA)
Cerebellum(M)(BalbC)
Cerebellum(M)(X129)
Brain(M)(BalbC)
central nervous system neuron axonogenesis [9]
neuron projection morphogenesis [50]
negative regulation of axonogenesis [8]
regulation of neuron projection development [14]
cell morphogenesis with neuron differentiation [53]
regulation of cell morphogenesis differentiation [14]
central nervous system neuron differentiation [14]
generation of neurons [90]
negative regulation of cell development [14]
cell part morphogenesis [54]
cell morphogenesis [83]
maternal placenta development [4]
cell aging [12]
oocyte development [8]
gamete generation [57]
chromatin remodeling [9]
chromatin modification [68]
neurofilament cytoskeleton organization [8]
cell projection organization [82]
microspike assembly [7]
cell junction assembly [9]
endomembrane system organization [8]
cell-cell adhesion mediated by integrin [4]
protein polyubiquitination [9]
regulation of protein modification process [36]
ubiquitin-dependent protein catabolic process [31]
glycosphingolipid biosynthetic process [3]
9-cis-retinoic acid biosynthetic process [3]
regulation of protein metabolic process [64]
negative regulation of macromolecule metabolism [83]
regulation of macromolecule biosynthetic process [365]
positive regulation of actin filament polymerization [5]
positive regulation of protein complex assembly [7]
positive regulation of cell-cell adhesion [5]
regulation of Rho protein signal transduction [26]
reg small GTPase mediated signal transduct [58]
Ras protein signal transduction [40]
T cell receptor signaling pathway [9]
immune response-activating receptor signaling [11]
activation of immune response [18]
membrane organization [68]
amino acid import [5]
acidic amino acid transport [8]
L-glutamate import [5]
cytoskeleton-dependent intracellular transport [10]
Golgi vesicle transport [15]
associative learning [9]
learning or memory [22]
response to UV [12]
response to acid [8]
Average
2-Year
Fold Change
(Old / Young)
> 1.25
1.20 - 1.25
1.15 - 1.20
1.10 - 1.15
1.05 - 1.10
0.95 - 1.05
0.90 - 0.95
0.85 - 0.90
0.80 - 0.85
0.75 - 0.80
< 0.75
▲▼ P < 0.05

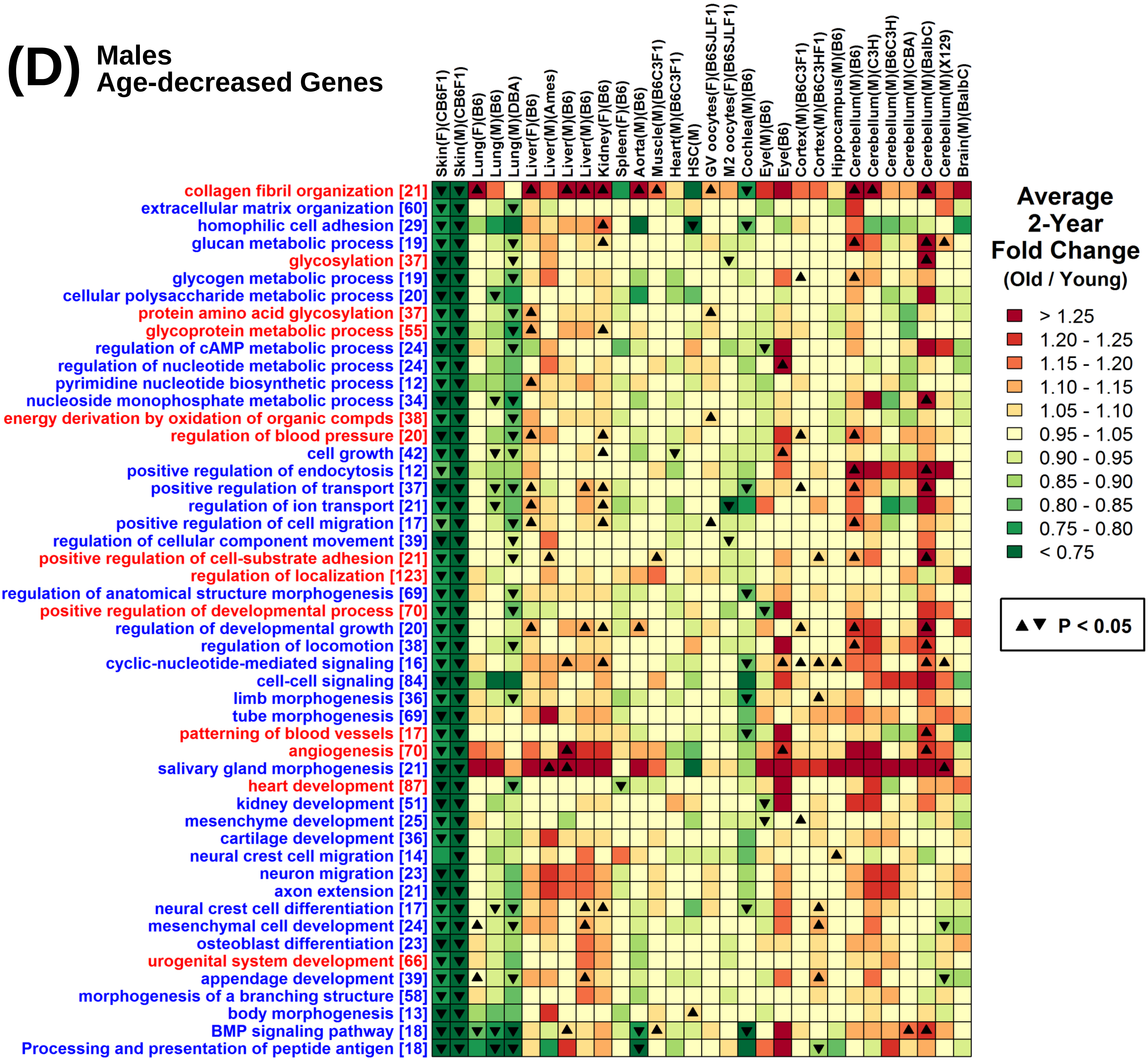
(D)
Males
Age-decreased Genes
Skin(F)(CB6F1)
Skin(M)(CB6F1)
Lung(F)(B6)
Lung(M)(B6)
Lung(M)(DBA)
Liver(F)(B6)
Liver(M)(Ames)
Liver(M)(B6)
Liver(M)(B6)
Kidney(F)(B6)
Spleen(F)(B6)
Aorta(M)(B6)
Muscle(M)(B6C3F1)
Heart(M)(B6C3F1)
HSC(M)
GV oocytes(F)(B6SJLF1)
M2 oocytes(F)(B6SJLF1)
Cochlea(M)(B6)
Eye(M)(B6)
Eye(B6)
Cortex(M)(B6C3F1)
Cortex(M)(B6C3HF1)
Hippocampus(M)(B6)
Cerebellum(M)(B6)
Cerebellum(M)(C3H)
Cerebellum(M)(B6C3H)
Cerebellum(M)(CBA)
Cerebellum(M)(BalbC)
Cerebellum(M)(X129)
Brain(M)(BalbC)
collagen fibril organization [21]
extracellular matrix organization [60]
homophilic cell adhesion [29]
glucan metabolic process [19]
glycosylation [37]
glycogen metabolic process [19]
cellular polysaccharide metabolic process [20]
protein amino acid glycosylation [37]
glycoprotein metabolic process [55]
regulation of cAMP metabolic process [24]
regulation of nucleotide metabolic process [24]
pyrimidine nucleotide biosynthetic process [12]
nucleoside monophosphate metabolic process [34]
energy derivation by oxidation of organic compds [38]
regulation of blood pressure [20]
cell growth [42]
positive regulation of endocytosis [12]
positive regulation of transport [37]
regulation of ion transport [21]
positive regulation of cell migration [17]
regulation of cellular component movement [39]
positive regulation of cell-substrate adhesion [21]
regulation of localization [123]
regulation of anatomical structure morphogenesis [69]
positive regulation of developmental process [70]
regulation of developmental growth [20]
regulation of locomotion [38]
cyclic-nucleotide-mediated signaling [16]
cell-cell signaling [84]
limb morphogenesis [36]
tube morphogenesis [69]
patterning of blood vessels [17]
angiogenesis [70]
salivary gland morphogenesis [21]
heart development [87]
kidney development [51]
mesenchyme development [25]
cartilage development [36]
neural crest cell migration [14]
neuron migration [23]
axon extension [21]
neural crest cell differentiation [17]
mesenchymal cell development [24]
osteoblast differentiation [23]
urogenital system development [66]
appendage development [39]
morphogenesis of a branching structure [58]
body morphogenesis [13]
BMP signaling pathway [18]
Processing and presentation of peptide antigen [18]
Average
2-Year
Fold Change
(Old / Young)
> 1.25
1.20 - 1.25
1.15 - 1.20
1.10 - 1.15
1.05 - 1.10
0.95 - 1.05
0.90 - 0.95
0.85 - 0.90
0.80 - 0.85
0.75 - 0.80
< 0.75
▲▼ P < 0.05
