# Supplementary material for: Meta-Profiles of Gene Expression during Aging: Limited Similarities between Mouse and Human and an Unexpectedly Decreased Inflammatory Signature
Source: PLoS One. 2012 Mar 7;7(3):e33204. doi: 10.1371/journal.pone.0033204 (PMC3296693; doi:10.1371/journal.pone.0033204)

(A) Similar Aging Effects in Humans and Mice

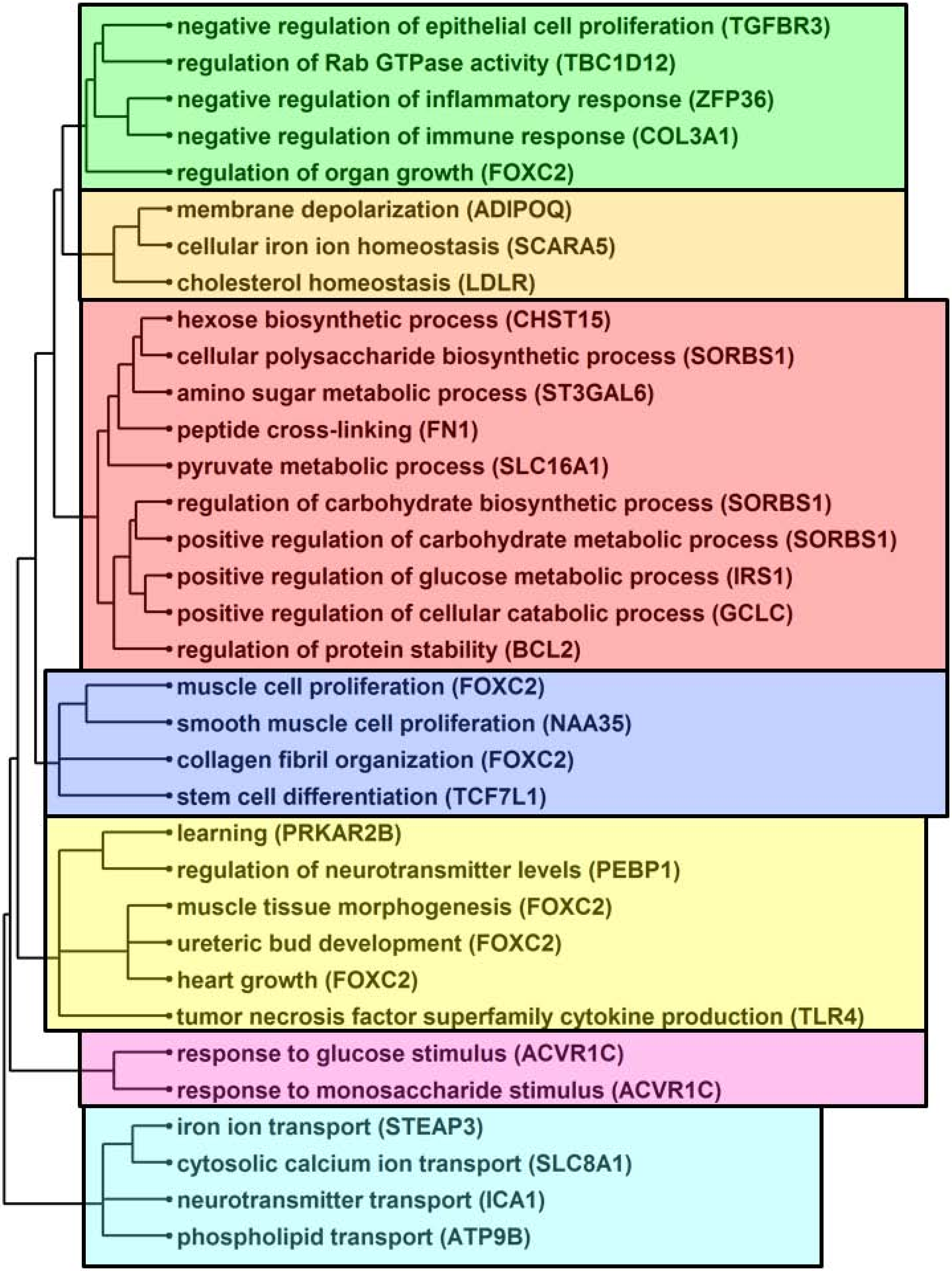

(B) Disimilar Aging Effects in Humans and Mice

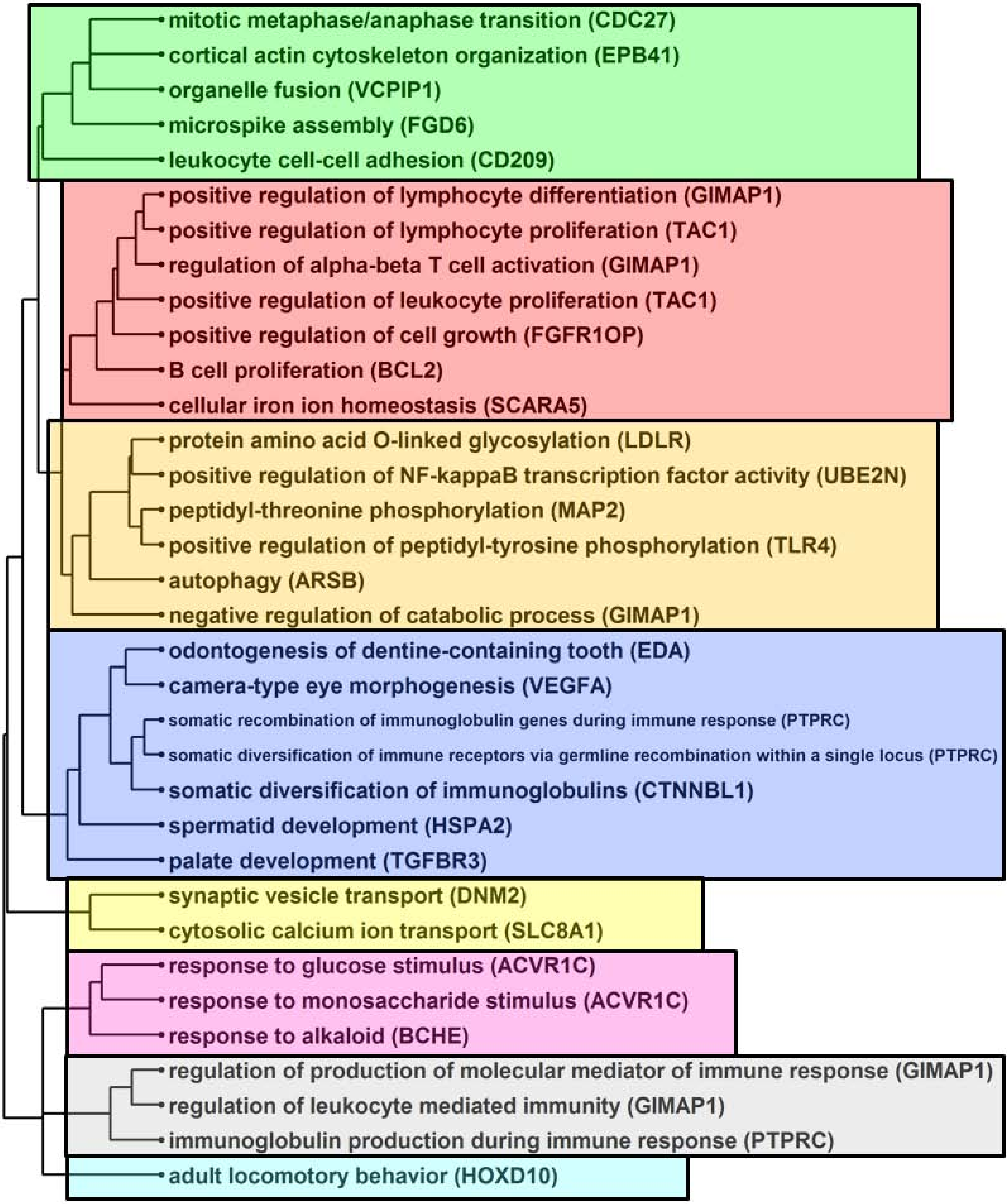

(C) Females – GO BP terms associated with similar aging effects

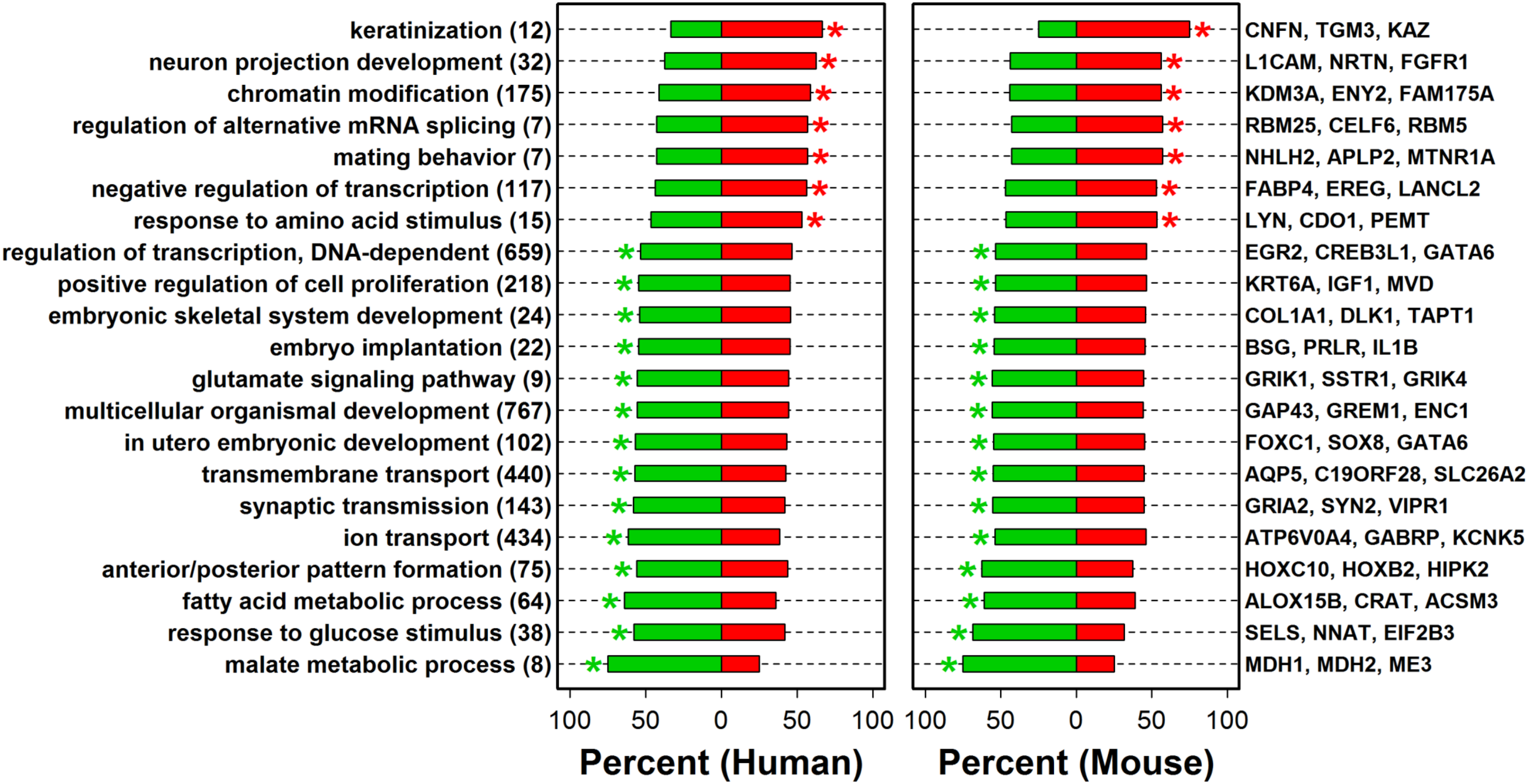

(D) Females – GO BP terms associated with dissimilar aging effects

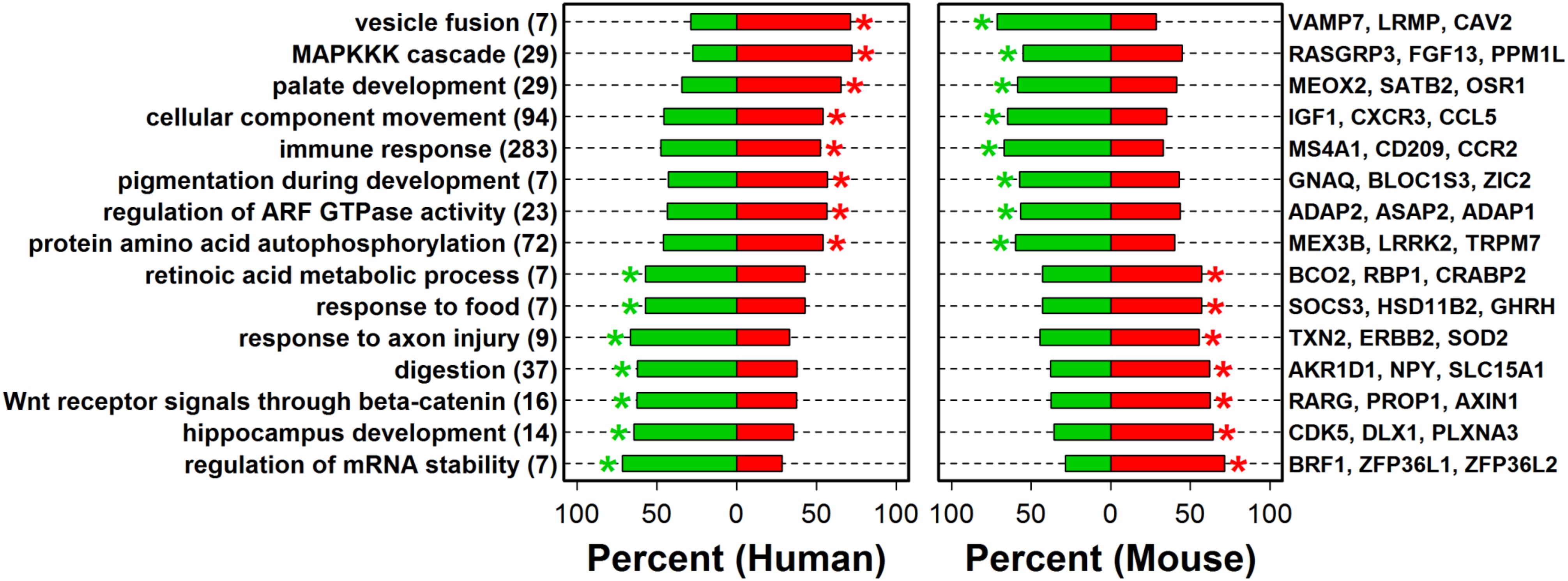

(E) Males – GO BP terms associated with similar aging effects

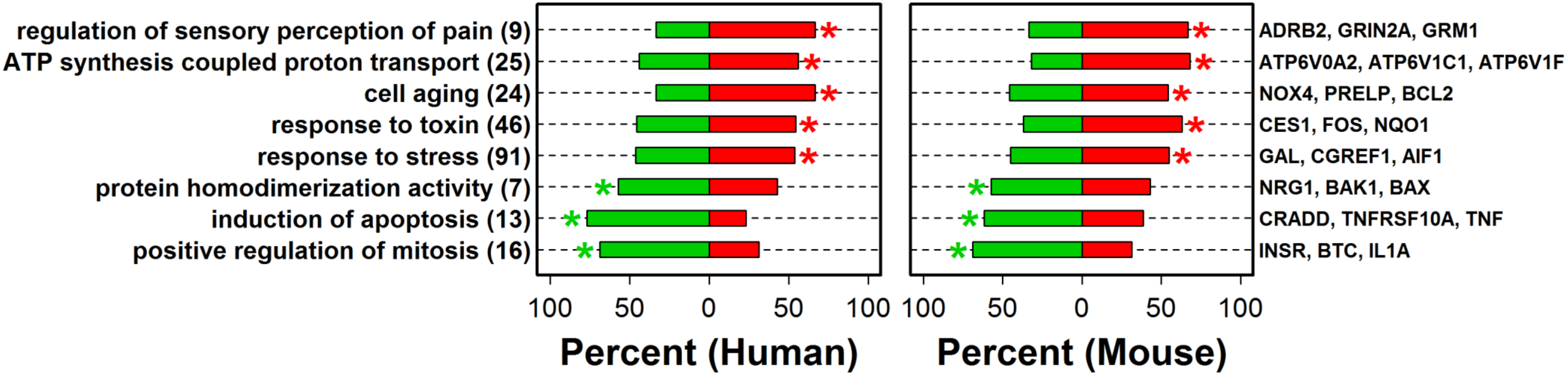

(F) Males – GO BP terms associated with dissimilar aging effects

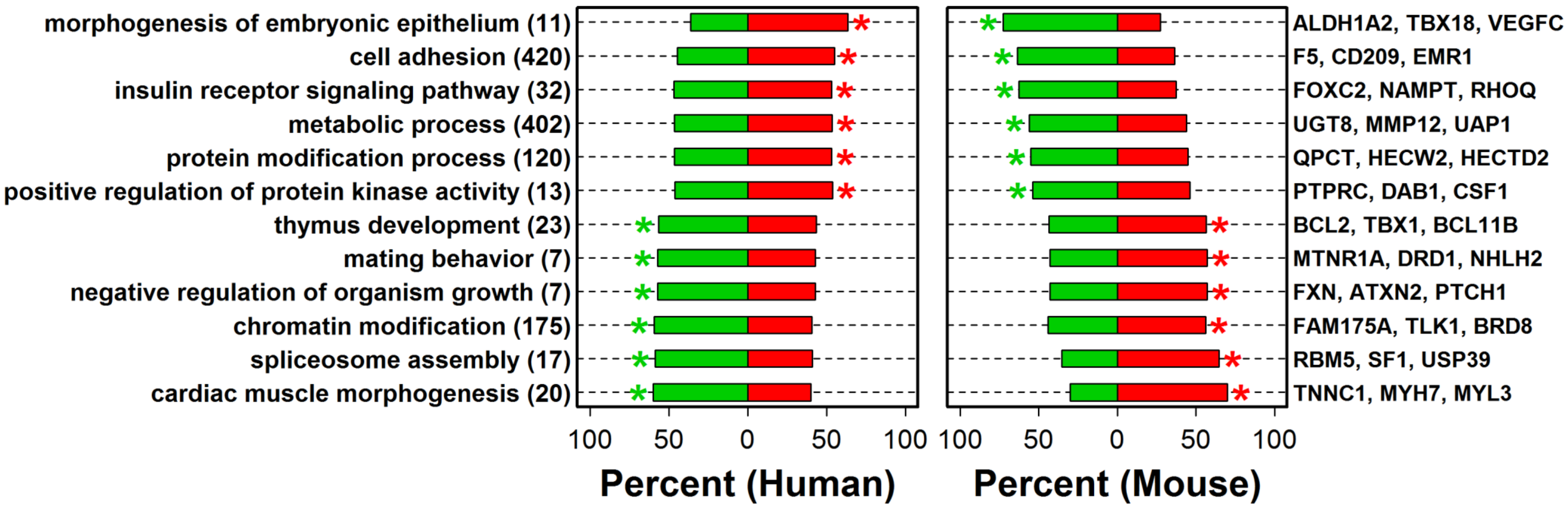

Supplement: Figure S19 — Biological processes associated with concordant and discordant age-associated expression patterns in human sun-protected skin and mouse tail skin. Figures (A) and (B) list gene ontology (GO) biological process terms significantly overrepresented (P<0.05) among genes with (A) concordant or (B) discordant age-associated expression patterns in humans and mice. The analysis was performed based upon (A) 2190 genes concordantly altered by aging in humans and mice (i.e., increased or decreased in the same sex of both species; P<0.05 for each species) and (B) 644 genes altered discordantly altered by aging in humans and mice (i.e., increased in one species and decreased in the other for the same sex; P<0.05 per species). GO terms in both (A) and (B) have been clustered based upon a similarity metric that is proportional to the number of shared ancestral terms in the GO hierarchy. For each term, an exemplar gene is listed in parentheses, which exhibits either a corresponding (in part A) or non-corresponding (in part B) expression pattern in humans and mice. As an alternative strategy (Figures C–F), we scored 5242 GO biological process terms (all GO terms associated with orthologous genes in humans and mice), and identified those for which the direction of aging effects (i.e., increase or decrease) was most similar or discordant in human and mouse skin (i.e., regardless of whether genes were significantly altered by aging in either species; see Figures C–F). For each GO term selected in this fashion, figures (C)–(F) display the proportion of age-increased and age-decreased genes in each species (red and green bars). The right margin of Figures (C)–(F) lists three exemplar genes associated with the selected terms. (PDF) [file pone.0033204.s019.pdf]
