# Supplementary material for: Meta-Profiles of Gene Expression during Aging: Limited Similarities between Mouse and Human and an Unexpectedly Decreased Inflammatory Signature
Source: PLoS One. 2012 Mar 7;7(3):e33204. doi: 10.1371/journal.pone.0033204 (PMC3296693; doi:10.1371/journal.pone.0033204)
Supplement: Table S1 — Human datasets (Affymetrix Human Genome U133 Plus 2.0 array). This table lists datasets used to evaluate the effects of aging on gene expression in skin and other human tissues. The table includes experimental details for each dataset along with information on the number of genes identified as significantly altered by aging (P<0.05 and FDR-adjusted P<0.05). (PDF) [file pone.0033204.s021.pdf]

Table S1. Human datasets (Affymetrix Human Genome U133 Plus 2.0 array). The table lists datasets used to evaluate the effects of aging on gene expression in skin and other human tissues. All data are available for download from Gene Expression Omnibus (see Database ID column). The cohort size lists the total number of individuals used for each dataset to evaluate the effects of aging on gene expression. Age-related gene expression patterns were identified using one of two statistical methods (A and B). For method A, a two-sample comparison was carried out between a group of younger subjects and a group of older subjects. For method B, a regression analysis was performed with gene expression (log_2-transformed) as the dependent variable and subject age as the independent variable. In each case, we identified the number of probe sets increased or decreased by aging (out of 54675 probe sets represented on the U133 Plus 2.0 array platform). The total number of age-altered probe sets was evaluated based upon a comparison-wise p-value of 0.05 (see 5th and 6th columns from the left). Additionally, we identified the number of probe sets increased or decreased by aging based upon an FDR-adjusted p-value of 0.05 (see last two columns on the right; P-values were adjusted using the Benjamini-Hochberg method). In each of the final four columns, the total number of age-increased or age-decreased probe sets is listed, with the number of unique genes associated with these probe sets listed in parentheses. Further experimental details and references for each dataset are listed below the table (see footnotes).

Experiment Label	Database ID	Cohort Size	Statistical Method	Age-Increased ($P < 0.05$)	Age-Decreased ($P < 0.05$)	Age-Increased (Adj $P < 0.05$)	Age-Decreased (Adj $P < 0.05$)
Skin (F) (18-75)[1]	GSE13355	31	B	4505 (2870)	4354 (2891)	713 (525)	991 (714)
Skin (M) (18-60)[1]	GSE13355	31	B	1519 (1064)	1556 (1113)	5 (4)	1 (1)
S Airway Epith (F) (22-62)[2]	GSE11906	8	B	3468 (2462)	3662 (2087)	0 (0)	0 (0)
S Airway Epith (M) (29-73)[2]	GSE11906	22	B	2713 (1791)	1849 (1365)	3 (3)	1 (1)
L Airway Epith (M) (31-61)[2]	GSE11906	11	B	6437 (4128)	5375 (3295)	10 (8)	32 (28)
Trachea (M) (34-60)[2]	GSE11906	13	B	2474 (1499)	5307 (3749)	0 (0)	15 (14)
Parotid Gland (19-69)[3]	GSE8764	8	B	1185 (856)	1269 (911)	0 (0)	0 (0)
Vast Lateral A (M) (32-71)[4]	GSE9419	22	A	4053 (2369)	5221 (3288)	112 (70)	26 (20)
Vast Lateral B (M) (32-71)[5]	GSE9419	22	A	2749 (1698)	2539 (1738)	30 (17)	2 (1)
Vast Lateral C (M) (32-71)[6]	GSE9419	22	A	3796 (2187)	2443 (1694)	231 (138)	22 (18)
Vast Lateral A (?)[7]	GSE9103	19	A	3388 (2365)	2395 (1539)	87 (71)	10 (8)
Vast Lateral B (?)[8]	GSE9103	18	A	5874 (3870)	4210 (2673)	207 (169)	57 (44)
Rect Abdominis (F) (29-81)[9]	GSE5086	25	B	376 (272)	653 (471)	0 (0)	0 (0)
Rect Abdominis (M) (24-83)[9]	GSE5086	37	B	1624 (1011)	2439 (1767)	4 (3)	11 (9)
Blood (F) (7-63)[10]	GSE19743	33	B	1411 (871)	1202 (806)	22 (18)	8 (4)
Blood (F) (17-40)[11]	GSE11375	9	B	1878 (1362)	2395 (1622)	1 (1)	3 (2)
Blood (M) (7-43)[10]	GSE19743	30	B	16867 (8653)	9774 (6319)	717 (511)	2182 (1724)
Blood (M) (18-54)[11]	GSE11375	17	B	4371 (2809)	2130 (1556)	3 (2)	4 (3)
Blood (23-64)[12]	GSE16028	22	B	2270 (1635)	2796 (1665)	5 (4)	14 (8)

Temporal Lobe (F) (26-99)[13]	GSE11882	18	B	3214 (2146)	4477 (2644)	49 (44)	57 (43)
Temporal Lobe (M) (20-97)[13]	GSE11882	21	B	3535 (1800)	4105 (3001)	210 (117)	211 (184)
Hippocampus (F) (26-99)[13]	GSE11882	20	B	4888 (3088)	5268 (3023)	1033 (747)	353 (238)
Hippocampus (M) (20-97)[13]	GSE11882	23	B	5276 (2801)	3445 (2513)	588 (373)	215 (177)
Parietal Lobe (F) (26-99)[13]	GSE11882	20	B	3618 (2264)	2514 (1690)	34 (26)	32 (28)
Parietal Lobe (M) (20-97)[13]	GSE11882	23	B	7456 (3769)	7683 (5046)	2438 (1404)	3184 (2411)
Frontal Lobe (F) (26-99)[13]	GSE11882	24	B	8075 (4288)	7135 (4410)	2074 (1289)	1960 (1408)
Frontal Lobe (M) (20-97)[13]	GSE11882	24	B	9033 (4579)	9347 (5787)	5035 (2789)	5392 (3656)

[1]Nair et al. 2009, Nat Genet 41:199-204. Punch biopsies were obtained from sun-protected regions (buttocks or upper thigh) of normal and apparently healthy individuals.

[2]Raman et al. 2009, BMC Genomics 10:493. Samples were obtained from healthy non-smokers using fiberoptic bronchoscopy.

[3]Srivastava et al. 2008, Arch Oral Biol 53:1058-70. Samples were obtained from healthy male and female subjects scheduled to have parotid surgery because their gland contained a benign tumor that required removal of all or a large portion of the gland. All samples were pathologically confirmed to be benign salivary gland tumors and RNA was extract from the benign tissue surrounding tumors.

[4]Thalacker-Mercer et al. 2007, Am J Clin Nutr 85:1344-52. Age-associated gene expression patterns were identiifed from a two-sample comparison between young (n = 12) and old (n = 10) subjects. These data were generated as part of a clinical study to evaluate the short-term effects of protein intake on the transcriptional profile of human muscle. Both young and old subjects in this comparison were provided a low-protein diet for 12 days prior to tissue biopsies (63% of the recommended daily allowance; 0.50 g/kg). Tissue biopsies were collected following an overnight fast.

[5]Thalacker-Mercer et al. 2007, Am J Clin Nutr 85:1344-52. Age-associated gene expression patterns were identified from a two-sample comparison between young (n = 12) and old (n = 10) subjects. These data were generated as part of a clinical study to evaluate the short-term effects of protein intake on the transcriptional profile of human muscle. Both young and old subjects in this comparison were provided an adequate-protein diet for 12 days prior to tissue biopsies (94% of the recommended daily allowance; 0.75 g/kg). Tissue biopsies were collected following an overnight fast.

[6]Thalacker-Mercer et al. 2007, Am J Clin Nutr 85:1344-52. Age-associated gene expression patterns were identified from a two-sample comparison between young (n = 12) and old (n = 10) subjects. These data were generated as part of a clinical study to evaluate the short-term effects of protein intake on the transcriptional profile of human muscle. Both young and old subjects in this comparison were provided a high-protein diet for 12 days prior to tissue biopsies (125% of the recommended daily allowance; 1.0 g/kg). Tissue biopsies were collected following an overnight fast.

[7]Age-associated gene expression patterns were identified from a two-sample comparison between young (n = 9) and old (n = 10) subjects. These data were generated as part of a study to evaluate the long-term effects of endurance training on the transcriptional profile of human muscle. Both young and old subjects from this group followed a sedentary lifestyle during the four years prior to tissue biopsies (exercised less than 30 minutes/day, twice per week).

[8]Age-associated gene expression patterns were identified from a two-sample comparison between young (n = 9) and old (n = 9) subjects. These data were generated as part of a study to evaluate the long-term effects of endurance training on the transcriptional profile of human muscle. Both young and old subjects from this group followed a vigorous exercise program during the four years prior to tissue biopsies (≥ 1 hour cycling or running 6 days/week).
[9]Zahn et al. 2006, PLoS Genet 2:e115. Muscle samples were obtained from patient biopsies collected either during surgery or in an outpatient procedure.
[10]Zhou et al. 2010, Proc Natl Acad Sci U S A 107:9923-8. Peripheral blood samples were obtained at a single time point from volunteers or from individuals undergoing elective operative interventions.
[11]Warren et al. 2009, Mol Med 15:220-7. Control subjects were recruited at Harborview Medical Center and the University of Texas Medical Branch-Galveston. Inclusion criteria consisted only of being in apparent good health.
[12]Karlovich et al. 2009, BMC Med Genomics 2:33. Subjects were enrolled in the study if they were at least 20 years of age and were healthy, as determined by medical history, physical examination, and standard laboratory test values. Exclusion criteria included abnormal laboratory test results, BMI > 32, hypertension, and a positive urine pregnancy test, or positive urine HCV, HBV or HIV test. Any subject who had a concomitant disease or condition that could interfere with clinical evaluations, was an active smoker, or was taking medication on a regular basis was also excluded.
[13]Bertold et al. 2008, Proc Natl Acad Sci U S A 105:15605-10. RNA was extracted from frozen unfixed tissue obtained from neuropathologically and neurologically normal controls aged 20–99 years.
