# Supplementary material for: Meta-Profiles of Gene Expression during Aging: Limited Similarities between Mouse and Human and an Unexpectedly Decreased Inflammatory Signature
Source: PLoS One. 2012 Mar 7;7(3):e33204. doi: 10.1371/journal.pone.0033204 (PMC3296693; doi:10.1371/journal.pone.0033204)
Supplement: Table S2 — Mouse Datasets (Affymetrix Mouse Genome 430 2.0 array). This table lists datasets used to evaluate the effects of aging on gene expression in skin and other mouse tissues. The table includes experimental details for each dataset along with information on the number of genes identified as significantly altered by aging (P<0.05 and FDR-adjusted P<0.05). (PDF) [file pone.0033204.s022.pdf]

Table S2. Mouse Datasets (Affymetrix Mouse Genome 430 2.0 array). The table lists datasets used to evaluate the effects of aging on gene expression in skin and other mouse tissues. Most datasets are available for download from Gene Expression Omnibus (see Database ID column). Age-related gene expression patterns were identified using one of two statistical methods (A and B). For method A, a two-sample comparison was carried out between a group of young mice and a group of older mice. For method B, a regression analysis was performed with gene expression ($\log_2$-transformed) as the dependent variable and mouse age as the independent variable. In each case, we identified the number of probe sets increased or decreased by aging (out of 45101 probe sets represented on the 430 2.0 array platform). The total number of age-altered probe sets was evaluated based upon a comparison-wise p-value of 0.05 (see 5th and 6th columns from the left). Additionally, we identified the number of probe sets increased or decreased by aging based upon an FDR-adjusted p-value of 0.05 (see last two columns on the right; P-values were adjusted using the Benjamini-Hochberg method). In each of the final four columns, the total number of age-increased or age-decreased probe sets is listed, with the number of unique genes associated with these probe sets listed in parentheses. Further experimental details and references for each dataset are listed below the table (see footnotes).

Experiment Label	Database ID	Age Range	Statistical Method	Age-Increased ($P < 0.05$)	Age-Decreased ($P < 0.05$)	Age-Increased (Adj $P < 0.05$)	Age-Decreased (Adj $P < 0.05$)
Skin(F)(CB6F1)[1]	N/A	5 - 30 months	A	4197 (2539)	4192 (2999)	3 (1)	16 (15)
Skin(M)(CB6F1)[1]	N/A	5 - 30 months	A	3946 (2412)	4244 (3092)	150 (101)	384 (332)
Lung(F)(B6)[2]	E-MEXP-1504	3 - 32 months	A	2580 (1971)	3513 (1936)	9 (5)	1 (0)
Lung(M)(B6)[3]	GSE6591	2 - 26 months	B	3775 (2728)	4671 (3286)	1012 (769)	1536 (1179)
Lung(M)(DBA)[3]	GSE6591	3 - 18 months	A	4059 (2795)	5031 (3330)	952 (703)	1071 (751)
Liver(F)(B6)[2]	E-MEXP-1504	3 - 32 months	A	2322 (1713)	1760 (1295)	103 (90)	10 (8)
Liver(M)(Ames)[4]	GSE3150	4 - 22 months	B	4980 (3400)	2682 (2078)	761 (563)	494 (390)
Liver(M)(B6)[5]	E-MEXP-839	2 - 32 months	B	6278 (4337)	5173 (3548)	2015 (1517)	1894 (1460)
Liver(M)(B6)[6]	GSE21716	6 - 24 months	B	4270 (2752)	4749 (3365)	1426 (994)	793 (601)
Kidney(F)(B6)[2]	E-MEXP-1504	3 - 32 months	A	2450 (1839)	2894 (2108)	119 (78)	7 (5)
Spleen(F)(B6)[2]	E-MEXP-1504	3 - 32 months	A	3144 (2133)	3668 (2277)	0 (0)	0 (0)
Aorta(M)(B6)[7]	GSE10000	2 - 20 months	B	14638 (9578)	6216 (3940)	10812 (7416)	4445 (2970)
Muscle(M)(B6C3F1)[8]	GSE11291	5 - 30 months	A	3883 (3015)	2847 (2149)	152 (131)	42 (31)
Heart(M)(B6C3F1)[8]	GSE11291	5 - 30 months	A	4906 (3622)	4170 (3080)	1657 (1326)	1665 (1338)
HSC(M)[9]	GSE4332	2.5 - 23 months	A	1714 (1213)	1986 (1598)	11 (10)	20 (18)
GV oocytes(F)(B6SJLF1)[10]	GSE11667	2 - 16 months	A	4476 (3355)	3594 (2889)	520 (427)	812 (705)
M2 oocytes(F)(B6SJLF1)[10]	GSE11667	2 - 16 months	A	6184 (4501)	5987 (4251)	2922 (2214)	3990 (2968)
Cochlea(M)(B6)[11]	GSE4786	4 - 15 months	A	53 (44)	103 (92)	0 (0)	0 (0)
Eye(M)(B6)[12]	GSE10965	4 - 26 months	A	5836 (3808)	7099 (4692)	3265 (2230)	2840 (2046)
Eye(B6)[13]	GSE22317	1.5 - 12 months	B	5087 (3642)	4659 (2949)	1236 (934)	1290 (870)
Cortex(M)(B6C3F1)[8]	GSE11291	5 - 30 months	A	3245 (2378)	2539 (1740)	222 (179)	82 (66)

Cortex(M)(B6C3HF1)[14]	GSE8150	5 - 30 months	A	9395 (6529)	6785 (4415)	6476 (4757)	3479 (2390)
Hippocampus(M)(B6)[15]	GSE13799	3 - 24 months	A	2941 (2261)	3632 (2638)	90 (74)	29 (23)
Cerebellum(M)(B6)[16]	N/A	5 - 25 months	A	2862 (2119)	1649 (1281)	19 (15)	4 (3)
Cerebellum(M)(C3H)[16]	N/A	5 - 25 months	A	5101 (3755)	3672 (2462)	986 (789)	510 (381)
Cerebellum(M)(B6C3H)[16]	N/A	5 - 25 months	A	2548 (2028)	2375 (1605)	7 (6)	1 (1)
Cerebellum(M)(CBA)[16]	N/A	5 - 25 months	A	3568 (2729)	3091 (2124)	189 (159)	88 (74)
Cerebellum(M)(BalbC)[16]	N/A	5 - 25 months	A	1945 (1488)	1402 (1075)	2 (1)	1 (1)
Cerebellum(M)(X129)[16]	N/A	5 - 25 months	A	1472 (1190)	1981 (1477)	23 (17)	1 (1)
Brain(M)(BalbC)[17]	GSE3253	3 - 22 months	A	1281 (1023)	934 (700)	1 (0)	0 (0)

[1]Age Groups: 5 months (*n* = 5); 30 months (*n* = 5). Data will be submitted to the Gene Expression Omnibus database prior to publication of this manuscript. Mice were fasted 5-6 hours prior to sacrifice and tissue collection and total RNA was isolated using standard methods.

[2]Schumacher et al. 2008, PLoS Genet 4:e1000161. Age Groups: 3 months (*n* = 3); 32 months (*n* =3). Naturally aged mice were kept on a regular diet and housed at the Animal Resource Center (Erasmus University Medical Center) and standard procedures were used to obtain total RNA.

[3]Misra et al. 2007, Physiol Genomics 31:429-40. Age Groups: 2 months (*n* = 3); 18 months (*n* = 3); 26 months (*n* = 3). Mice were procured from the National Institute on Aging (Bethesda, MD) or purchased from Jackson Laboratories (Bar Harbor, ME). Mice were housed in an antigen- and virus-free room and were provided with water and a pelleted stock diet ad libitum.

[4]DeFord et al. 2006, Age (Dordr) 28:125-44. Age Groups: 4 months (*n* = 10); 10 months (*n* = 10); 22 months (*n* = 11). Mice were purchased from the Jackson Laboratory and maintained as a colony at the University of Texas Medical Branch, Galveston, USA.

[5]Niedernhofer et al. 2006, Nature 444:1038-1043. Age Groups: 2 months (*n* = 2); 4 months (*n* = 4); 24 months (*n* = 4); 32 months (*n* = 4).

[6]Lee et al. 2011, PLoS One 6:e24381. Age Groups: 6 months (*n* = 4); 12 months (*n* = 4); 18 months (*n* = 4); 24 months (*n* = 4). Male C57BL/6 mice, at approximately 6, 12, 18 and 24 months of age, were obtained from Charles River Laboratory (Raleigh) and acclimated for 1 week. Mice were housed in polycarbonate cages on Alpha Dry bedding with a 12-hour light/dark cycle. Room temperature was 70 ± 2° F with a relative humidity of 50%. The basal diet was Ralston Purina 5001 and water was provided ad libitum. Animals were sacrificed using CO2 asphyxiation.

[7]Gräbner et al. 2009, J Exp Med 206:233-48. Age Groups: 2 months (*n* = 3); 8 months (*n* = 3); 20 months (*n* = 3). Wild-type mice on the C57BL/6J genetic background were maintained on a standard mouse chow. Total aortae were removed at the age of 2, 8, or 20 months and microarrays were prepared from total RNA extracts.

[8]Barger et al. 2008, PLoS One 3:e2264. Age Groups: 5 months (*n* = 5); 30 months (*n* = 5). Male C57BL/6×C3H/He F1 hybrid mice were purchased at six weeks of age (Harlan-Teklad), individually housed in a specific pathogen free facility and fed a control diet based on the AIN-93M formulation (Bio-Serv, Frenchtown, NJ).

[9]Rossi et al. 2005, Proc Natl Acad Sci U S A 102(26):9194-9. Age Groups: 2.5 months (*n* = 3); 23 months (*n* = 5). Old and middle-aged mice were obtained from the National Institute of Aging (Bethesda), and young mice were obtained from the Stanford University Laboratory Animal Facility. Long-term HSCs (LT-HSCs) were isolated by lineage depletion of whole BM using unconjugated antibodies (CD3, CD4, CD5, CD8, IL7Rα, B220, Ter119, Gr1, and Mac1) and Dynabeads M-450 beads (Dynal, Oslo).
[10]Pan et al. 2008, Dev Biol 316:397-407. Age Groups: 2 months (*n* = 4); 16 months (*n* = 4). B6SJLF1 female mice were purchased from Jackson Laboratory. Four sets of oocytes (25 oocytes/sample) and eggs (35 eggs/sample) were collected for microarray analysis from mice 6–8 weeks and 15 months old.
[11]Someya et al. 2007, Neurobiol Aging 28:1613-22. Age Groups: 4 months (*n* = 3); 15 months (*n* = 3). Four cochleae from two mice were pooled for each sample because the amount of mRNA from both cochleae of one mouse was insufficient.
[12]Chen et al. 2008, PLoS One 3:e2339. Age Groups: 4 months (*n* = 4); 26 months (*n* = 4). C57BL/6 male mice were obtained from NIA and housed under standard laboratory conditions. Mice neural retina and RPE/choroid were separated and placed in RNA lysis buffer and RNA was extracted by using the RNeasy Minikit (Qiagen).
[13]Parapuram et al. 2010, PLoS One 5:e13885. Age Groups: 1.5 months (*n* = 4); 5 months (*n* = 4); 12 months (*n* = 4). Five nanograms of total RNA from flow-sorted rod photoreceptors were used for microarray analyses.
[14]Reiter et al 2007, Mol Aspects Med 28:668-91. Age Groups: 5 months (*n* = 5); 30 months (*n* = 5).
[15]Pawlowski et al. 2009, Brain Res 1256:101-10. Age Groups: 3 months (*n* = 6); 24 months (*n* = 6). Male C57BL/6J Nia mice were housed individually in clear polypropylene cages in a temperature- and humidity-controlled room with an artificial 12 h:12 h light:dark cycle, lights on at 0700 h. Standard pelleted chow and water were freely available at all times. Older mice analyzed in this comparison include only those with good spatial memory status as determined from behavioral assessment.
[16]Park et al. 2009, Aging Cell 8: 484-95. Age Groups: 5 months (*n* = 5); 25 months (*n* = 5). Calculated expression values were obtained from the supplemental material provided by Park et al. (2009). Mice were purchased from Harlan Sprague-Dawley at 6–7 weeks of age. Mice were housed singly in a pathogen-free facility and provided acidified water ad libitum. Each mouse was fed 84 kcal per week of AIN-76A diet. Mice were euthanized by rapid cervical dislocation and tissues were immediately frozen in liquid nitrogen and stored at −80 °C.
[17]Godbout et al. 2005, FASEB J 19:1329-31. Age Groups: 3 months (*n* = 3); 22 months (*n* = 3). Male BALB/c mice from a specific pathogen free colony were used. Mice were housed in polypropylene cages and maintained at 21°C under a reverse phase 12 h light-dark cycle with ad libitum access to water and rodent chow. Mice used in this young-old comparison comprised the control treatment from a study designed to investigate effects of immune system activation. For these mice, treatment was limited to a single intraperitoneal (ip) saline injection prior to euthanasia by CO2 asphyxiation. Upon death, the brain was removed, separated in half at the longitudinal fissure, frozen in liquid nitrogen and stored at -80C prior to RNA extraction.
