# Supplementary material for: Meta-Profiles of Gene Expression during Aging: Limited Similarities between Mouse and Human and an Unexpectedly Decreased Inflammatory Signature
Source: PLoS One. 2012 Mar 7;7(3):e33204. doi: 10.1371/journal.pone.0033204 (PMC3296693; doi:10.1371/journal.pone.0033204)
Supplement: Table S3 — Empirical estimates of nucleotide frequency background distributions (human hg19 and mouse mm9). Human and mouse genomes were searched to identify matches with respect to position weight matrices representing motifs associated with known mammalian transcription factors. This table lists the region-specific estimates of the nucleotide frequency background distribution used for the calculation of position weight matrices. (PDF) [file pone.0033204.s023.pdf]

Table S3. Empirical estimates of nucleotide frequency background distributions (human hg19 and mouse mm9). Human and mouse genomes were searched to identify matches with respect to position weight matrices representing motifs associated with known mammalian transcription factors. Position weight matrices were calculated using region-specific estimates of the nucleotide frequency background distribution. For each region, the background frequency distribution was estimated genome-wide, using all identified sequences 2000 BP upstream / 200 BP downstream of transcription start sites (TSSs), non-coding intergenic sequences (repeat-masked), intronic sequences (repeat-masked), or conserved sequences 2000 BP upstream of TSSs (from UCSC multiple alignments of vertebrate genomes).

Species	Genome Region	A	C	G	T
Human	2000 BP upstream of TSS, 200 BP downstream of TSS[1]	0.247	0.251	0.254	0.248
	Non-coding intergenic sequence[2]	0.300	0.200	0.200	0.300
	Intronic sequence[2]	0.297	0.202	0.202	0.298
	Conserved 2000 BP upstream of TSS[2]	0.252	0.249	0.248	0.251
Mouse	2000 BP upstream of TSS, 200 BP downstream of TSS[3]	0.255	0.243	0.246	0.256
	Non-coding intergenic sequence[4]	0.298	0.202	0.202	0.298
	Intronic sequence[4]	0.289	0.211	0.211	0.289
	Conserved 2000 BP upstream of TSS[4]	0.264	0.237	0.237	0.262

[1]Background frequencies were used in Figure S3, S4 and S5 calculations
[2]Background frequencies were used in Figure S6 calculations
[3]Background frequencies were used in Figure 8, S15 and S16 calculations
[4]Background frequencies were used in Figure S17 calculations
